# Vacuolar iron transporters mediate resistance to triadimefon in plant pathogenic fungi

**DOI:** 10.1038/s41467-026-72157-6

**Published:** 2026-05-06

**Authors:** Fan Ji, Youwei Du, Yaning Liu, Bofan Liu, Xinpei Gao, Yaoxuan Feng, Xinyun Liu, Lili Huang, Jun Guo, Zhensheng Kang, Gangming Zhan

**Affiliations:** 1 College of Plant Protection, Northwest A & F University, Yangling, 712100 P. R. China; 2 State Key Laboratory for Crop Stress Resistance and High-Efficiency Production, Northwest A & F University, Yangling, 712100 P. R. China

**Keywords:** Fungal biology, Translation, Chemical genetics

## Abstract

Triadimefon resistance in *Puccinia striiformis* f. sp. *tritici* (*Pst*), the fungal pathogen of wheat stripe rust, is increasingly observed in China, but the mechanisms beyond cytochrome P450 14α-demethylase (*Cyp51*) mutations remain unknown. Through bulked segregant analysis and RNA-seq of a sexual *Pst* population, we identify three vacuolar iron transporter genes (*CCC1*), *PstCCC1.1*, *PstCCC1.2* and *PstCCC1.3*, as key triadimefon sensitivity determinants. Functional analyses show that RNAi-mediated silencing of these genes reduce fungicide resistance, while heterologous expression of natural mutants (*PstCCC1.2*^*R82C,S86P,E109K*^ and *PstCCC1.3*^*R306C,S310P,E333K*^) in yeast and *Fusarium graminearum* reduce cytosolic iron content and thereby increase resistance to triadimefon. Combined microscale thermophoresis (MST) and isothermal titration calorimetry (ITC) show that these two mutants exhibit increased iron affinities, linking specific amino acid changes to the detoxification ability. This iron-mediated resistance mechanism is conserved across fungal species and represents a triazole resistance pathway independent of Cyp51, with implications for managing fungal diseases, especially stripe rust.

## Abstract (web summary)

This study identifies vacuolar iron transporters as key drivers of triazole fungicide resistance across fungal species, thereby uncovering a fungicide resistance mechanism mediated by iron detoxification and independent of the traditional target *Cyp51*.

## Introduction

Wheat, one of the most important food crops in the world, feeds 40% of the world’s population and provides 20% of the energy and protein needed by humans^1^. Wheat stripe rust, caused by biotrophic fungus *Puccinia striiformis* f. sp. *tritici* (*Pst*), is prevalent across wheat-growing regions in the world, often resulting in substantial yield losses^2^. Effective prevention and management remain an ongoing international challenge^3^. The timely application of fungicides is crucial for controlling stripe rust, as it helps to mitigate yield losses and ensures stable production^4,5^. However, the repeated, large-scale use of single mode of action fungicides raises the risk of developing fungicide-resistant isolates^6^.

The most widely used fungicide to control stripe rust in China is triadimefon, the first demethylation inhibitor (DMI) fungicide developed and marketed by Bayer in 1976^7^. During the wheat-growing season, fungicides are typically applied once or twice in fields planted with cultivars without adequate resistance to stripe rust^8^. Due to the high efficacy, low toxicity and broad-spectrum action, DMI fungicides have been used to control stripe rust for nearly 50 years^9^. The prolonged use of such a single type of fungicide has increased the likelihood of resistance in *Pst*^3,10^.

The primary mechanism of DMI resistance in fungi often involve mutations in or overexpression of the target gene, *Cyp51*, which encodes the enzyme sterol 14α-demethylase^11,12^. In *Pst*, the Y134F mutation in Cyp51 has been correlated with triadimefon resistance in field populations^13^. However, heterologous expression assays suggested that this mutation alone may not fully explain resistant phenotypes^13^. Furthermore, a growing proportion of triadimefon-resistant *Pst* isolates lack any mutations in *Cyp51* or show alteration in its expression^8,14^, strongly implying the existence of alternative, non-target site resistance mechanisms. The cellular basis of fungal growth inhibition by DMI fungicides extends beyond ergosterol depletion. DMI-induced stress can disrupt the cellular environment such as pH and calcium homeostasis^15,16^, potentially triggering oxidative stress^17,18^. While adaptive responses to DMI fungicides, including the differential regulation of efflux pumps and metabolic genes, are well-documented in human fungal pathogens^19–21^, the resistance mechanisms in obligate biotrophs like *Pst* remain poorly understood.

Intriguingly, iron homeostasis has emerged as a potential player in fungal stress responses. Fungi lack ferritin and instead sequester excessive iron into vacuoles for storage and detoxification, a process primarily mediated by vacuolar iron transporters like CCC1^22^. Given that DMI fungicides target Cyp51, a heme-containing iron protein, it is plausible that their action disrupts cellular iron homeostasis, contributing to toxicity. Indeed, recent studies prove the link between iron metabolism and antifungal efficacy^23^. However, the role of iron transporters in fungicide resistance in plant pathogenic fungi has not been explored.

To test the hypothesis that vacuolar iron transporters are involved in DMI fungicide resistance, we developed an F_2_ uredinial population of *Pst* through crossing a low triadimefon-resistant isolate (EC_50_ = 1.56 μg/mL) with a sensitive isolate (EC_50_ = 0.07 μg/mL) on plants of alternate host *Berberis aggregata*. The low resistant isolate exhibited neither point mutations in the *Cyp51* gene nor upregulation of the gene expression following triadimefon treatment. The sensitive isolate has the identical *Cyp51* sequence as the low resistant isolate. After assessing the reactions of the F_1_ and F_2_ progeny isolates to triadimefon through a detached leaf assay, resistant and sensitive DNA bulks, each comprising 20 F_2_ isolates, were prepared with equal quantity of DNA samples. High-throughput sequencing analysis of these DNA pools identified multiple single-nucleotide polymorphisms (SNPs) on chromosome 6 (Chrom6) and chromosome 11 (Chrom11) of Pst using the Unified Genotype module of Genome Analysis Toolkit (GATK) software. The transcriptome sequencing to investigate the expression variations of the genes on Chrom6 and Chrom11 following triadimefon treatment further identified *PstAZ2B06G00574* (*PstCCC1.1*), *PstAZ2B06G00575* (*PstCCC1.2*) and *PstAZ2B06G00587* (*PstCCC1.3*) on Chrom6 as candidate genes for triadimefon resistance. Further assays were conducted to determine how these genes are involved in triadimefon resistance. The segregating population established through crossing a resistant isolate and a sensitive isolate offered advantages over analyzing heterogeneous field isolates. It reduced genetic background noise, enabled clear segregation of monogenic resistance traits for precise mapping of related genes, and provided a uniform background for subsequent functional validation. Importantly, the insights found from the segregating population could be subsequently validated using natural field isolates.

Here, we report a Cyp51-independent resistance mechanism to triadimefon in *Pst*, mediated by specific mutations in vacuolar iron transporter genes. Through genetic mapping and functional validation, we identified three *PstCCC1* genes as key determinants of triadimefon sensitivity. We demonstrated that gain-of-function mutations in PstCCC1.2 and PstCCC1.3 enhance vacuolar iron sequestration, which reduces cytosolic iron accumulation and thereby confers resistance by mitigating triadimefon-induced oxidative damage. This iron-mediated resistance mechanism is conserved across fungal species, revealing a mechanism of fungicide resistance and offering strategies for disease management.

## Results

### Mapping of the candidate genes for triadimefon resistance in *Pst* through bulked segregant analysis combined with sequencing (BSA-seq) and RNA sequencing (RNA-seq)

F_1_ and F_2_ isolates were obtained through crossing low triadimefon-resistant isolate YQ324 (EC_50_ = 1.56 μg/mL) and sensitive isolate Gui1-2 (EC_50_ = 0.07 μg/mL) on barberry plants and separating on susceptible wheat plants (cv. MX169). The parental, F_1_ and F_2_ isolates were tested for triadimefon sensitivity using the detached leaf method^8^. The 16 tested F_1_ isolates had EC_50_ levels that ranged from 1.02 to 3.18 μg/mL (Supplementary Fig. 1; Supplementary Data 1). The F_1_ isolate (Za 6-7) with the highest EC_50_ value (3.18 μg/mL) was selected to develop the F_2_ population. The 82 F_2_ isolates had a much wider range of EC_50_ values ranging from 0.14 to 6.93 μg/mL (Supplementary Fig. 2; Supplementary Data 2). The observed segregation of resistant (EC₅₀ > 1.00 μg/mL) and sensitive (EC₅₀ < 1.00 μg/mL) phenotypes in the F₂ population fitted a 3:1 ratio (χ² = 0.27, *P* = 0.60), supporting the hypothesis that a single locus controls triadimefon sensitivity (Supplementary Data 3).

**Fig. 1:**
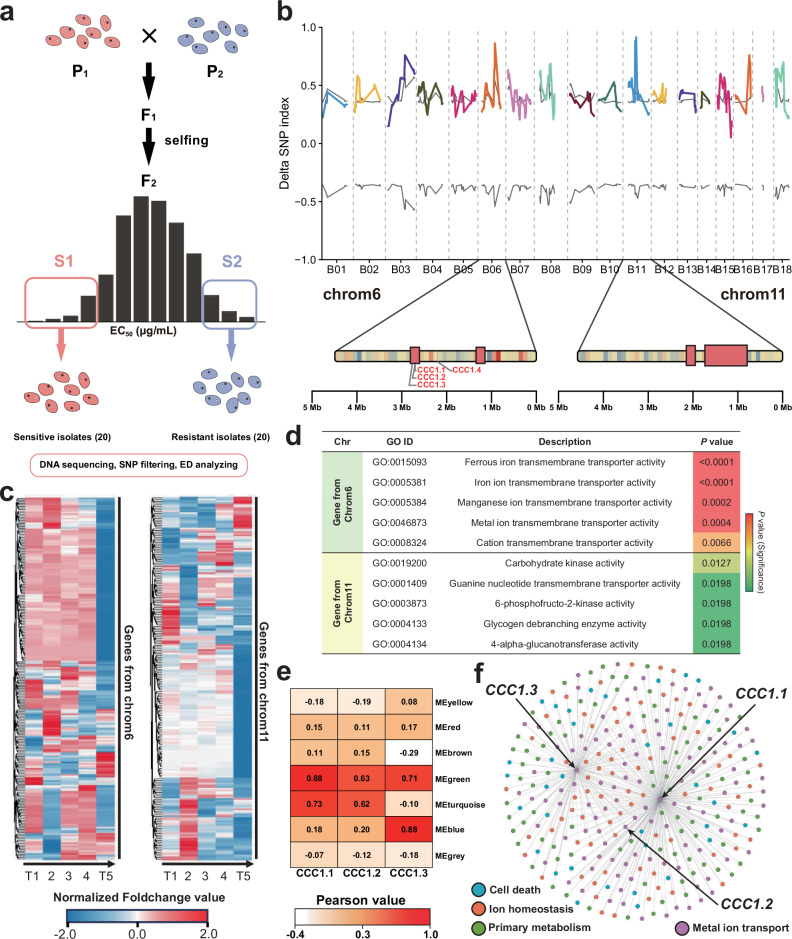
Identification of candidate genes controlling triadimefon resistance in *Puccinia striiformis* f. sp. *tritici* (*Pst*). **a** A simplified scheme for bulk segregant analysis sequencing (BSA-seq), single nucleotide polymorphism (SNP) filtering and expression data (ED) analysis for identifying genes associated with triadimefon resistance. **b** Identification of triadimefon resistance loci via BSA-seq in an F₂ population derived from a cross between the resistant isolate (YQ324) and the sensitive isolate (Gui1-2). The ΔSNP-index profile across chromosomes is shown with a gray line indicating the 99% confidence threshold (10,000 permutations). Significant quantitative trait loci (QTL) were localized to two genomic regions: a ~ 0.36 Mb interval on chromosome 6 and a ~ 1.1 Mb interval on chromosome 11. The identified QTL intervals are outlined by red rectangles, and the position of a candidate *CCC1* family gene is indicated in red text. **c** Heatmap showing expression abundances of genes on chromosomes 6 and 11 in response to triadimefon treatment. T1-T5 represent the time points at 0, 24, 48, 72, and 120 hours post-triadimefon treatment, respectively. The high and low expression abundances are shown in red and blue, respectively. **d** Gene ontology (GO) enrichment analysis on differentially expressed genes (DEGs) from Chrom6 and Chrom11. Histogram width and color represent the relative gene number and significance (*P* < 0.05) of related GO terms, respectively. DEGs from Chrom6 mainly functioned in iron transportation. DEGs from Chrom6 mainly functioned in iron transportation. The significance of each GO term was performed using two-sided Fisher’s exact test with Benjamini–Hochberg (BH) correction for multiple comparisons. **e** Heatmap displaying Module-trait relationships. Each row represents a module eigengene, and column represents a trait. Each cell contains the corresponding correlation and the *p* value. The table is color-coded for correlation based on the color legend. Statistical significance was calculated using a two-sided Pearson correlation test. **f** Weighted co**-**expression network illustrating the close relationships among *PstCCC1.1*, *PstCCC1.2*, *PstCCC1.3* and genes relevant to metal ion transport during triadimefon treatment. Genes associated with cell death, iron homeostasis, metal ion transport and primary metabolism are highlighted in blue, orange, purple and green, respectively.

To identify the genomic region contributing to triadimefon resistance in *Pst*, BSA mapping through next-generation sequencing and traditional map-based cloning were conducted. The DNA bulks were made for each of the two classes of phenotypes using 20 F_2_ isolates for each class with the lowest values and highest EC_50_ values for the sensitive and resistant classes, respectively. The two bulks and the DNA extracted from the two parents were subjected to whole-genome resequencing (Fig. 1a). A total of 39.89 Gb clean sequences were obtained after filtration, including 8.69 or 9.09 Gb reads for the parents and 10.32 or 11.79 Gb for the two bulks. After aligning the reads to the *Pst* AZ2 reference genome (ASM3951922v1) to identify variants, 8,328 SNPs were retained in each bulk. SNPs between the two bulks were identified and the number of isolates for each allele was counted for each SNP. The linkage probability of each SNP was plotted against its corresponding reference genome, which mapped possible candidate genes to a 0.36 Mb region (1.16–1.34 Mb; 2.62–2.80 Mb) on Chrom6 and 1.1 Mb region (0.80–1.72 Mb; 1.96–2.14 Mb) on Chrom11 (Fig. 1b).

To identify the major resistance-conferring gene(s), gene expression was analyzed using the RNA-seq dada from three biological replicates of low-resistant isolate YQ324 collected at different time points after 10 µg/mL triadimefon treatment. Totally, 15,050 transcripts were identified from all samples. Principal component analysis on the transcriptome profiles displayed distinct variations in transcription patterns at different time points, suggesting that transcription events involved in the response of *Pst* to triadimefon (Supplementary Fig. 3a). We categorized all expressed genes in two representative cluster modules according to their expression trend, and the expression of these genes continued to up- or down-regulated in *Pst* in response to the triadimefon treatment (Supplementary Fig. 3b). Function enrichment showed that these genes mainly exerted activities of metal ion transmembrane transporter, cation transmembrane transporter and ion transmembrane transporter (Supplementary Fig. 3c), suggesting the potential importance of metal ion homeostasis of *Pst* in response to the triadimefon treatment.

Subsequently, we analyzed the expression profiles of the genes from Chrom6 and Chrom11 of *Pst* following the triadimefon treatment. The expression levels of 52 genes on Chrom6 were significantly altered in *Pst* by the triadimefon treatment, and most of these genes were significantly activated to higher expression abundances (Fig. 1c), implying their involvement in triadimefon sensitivity. In contrast, the genes from Chrom11 did not significantly respond to the triadimefon treatment. Further gene ontology (GO) enrichment analysis on the differentially expressed genes (DEGs) from Chrom6 showed that numerous enriched GO terms are related to iron transport, especially ferrous iron transmembrane transporter and iron ion transmembrane transporter activities (Fig. 1d; Supplementary Data 4), suggesting the potential involvement of iron transporter in triadimefon sensitivity. However, no iron transport GO terms were significantly found on Chrom11 (Fig. 1d; Supplementary Data 5). Thus, only the region containing iron transporter genes on Chrom6 was found to be involved in triadimefon resistance, consistent with the single region for resistance from the genetic analysis.

On Chrom6, missense mutations were identified in 92 genes, and these genes were found to be involved in iron transportation. To identify the hub genes that conferred triadimefon resistance of *Pst*, we constructed a WGCNA network among genes related to iron transport and identified three genes with hub functions in the network, including *PstCCC1.1*, *PstCCC1.2* and *PstCCC1.3* from Chrom6 (Fig. 1e; 1f). Thus, we hypothesized that the missense mutations of *PstCCC1.1*, *PstCCC1.2* and *PstCCC1.3* confer resistance to triadimefon and focused on the three genes in the following experiments and analyses.

### Sequence analysis and expression patterns of candidate resistance genes

As shown in Supplementary Fig. 4a, the three candidate genes all contain the CCC1 domain, which is involved in ion transportation to vacuoles for maintaining iron homeostasis during cytoplasmic iron imbalance^22,24^. *PstCCC1.1* is 1,029 bp in length, encoding 343 amino acids with a molecular weight of 37.5 kDa. *PstCCC1.2* is 525 bp in length, encoding 174 amino acids with a molecular weight of 19.1 kDa. *PstCCC1.3* is 1,194 bp, encoding 398 amino acids with a molecular weight of 43 kDa. Phylogenetic analysis indicates that this domain is conserved in multiple fungal species (Supplementary Fig. 4b). As shown in Supplementary Fig 4c, PstCCC1.1 and PstCCC1.2 have 63.82% and 34.67% similarity to PstCCC1.3, respectively.

Expression analysis revealed distinct patterns among the three *PstCCC1* genes. Under triadimefon treatment, *PstCCC1.3* showed the highest expression level, while *PstCCC1.2* had a higher basal expression than *PstCCC1.1* (Supplementary Fig. 5a). The time-course expression profiles showed that all three genes were significantly upregulated at 12, 24, 48, and 72 hours after triadimefon treatment in both resistant and sensitive isolates (Supplementary Fig. 5b–d). Moreover, the upregulations of *PstCCC1.2* and *PstCCC1.3* exhibited stronger responsiveness to triadimefon treatment than *PstCCC1.1*. These results demonstrate that all three *PstCCC1* genes respond to triadimefon but with distinct expression profiles, with *PstCCC1.3* showing the highest early induction.

### Silencing *PstCCC1.1*, *PstCCC1.2* and *PstCCC1.3* reduced triazole resistance in a low-resistant *Pst* isolate via disrupting cytosolic iron content

To investigate the roles of *PstCCC1.1*, *PstCCC1.2* and *PstCCC1.3* in triadimefon sensitivity, we silenced them using a host-induced gene silencing (HIGS) approach^25^. After 10 days of the treatment with PDS (phytoene desaturase, a positive control for silencing efficiency), clear photobleaching symptoms were observed in wheat leaves, indicating that the HIGS system mediated by Barley Stripe Mosaic Virus (BSMV) was effective (Supplementary Fig. 6a). On this basis, *Pst* inoculation and triadimefon treatment experiments were conducted on the silenced plants of the candidate genes. To confirm the efficacy of the silencing system, we measured the transcript levels of the target genes, and found that the expression levels of *PstCCC1.1*, *PstCCC1.2*, and *PstCCC1.3* were significantly reduced by 46.9% to 67.9% compared to the control plants (Supplementary Fig. 6b). The RT-PCR analysis confirmed that the HIGS-mediated silencing of each of the *PstCCC1* genes did not affect the transcript levels of the other two genes (Supplementary Fig. 7a), supporting the specificity of the silencing system. Ten days after the triadimefon treatment, the urediniospore production of the silenced isolate was substantially lower than that of the controls (Supplementary Fig. 6c). To further confirm that silencing the three candidate genes can reduce the resistance of *Pst* to triadimefon, the mycelial development of silenced *PstCCC1.1*, *PstCCC1.2* and *PstCCC1.3* after the triadimefon treatment was observed under a microscope. Histological observations revealed that after 72 hours of triadimefon treatment, mycelial growth was significantly inhibited in the silenced isolates (Supplementary Fig. 6f, g). Although the triadimefon treatment did not induce significant changes in the total iron content of *Pst* (Supplementary Fig. 6d), the cytosolic iron content was significantly elevated in the silenced isolates under the treatment (Supplementary Fig. 6e).

**Fig. 2:**
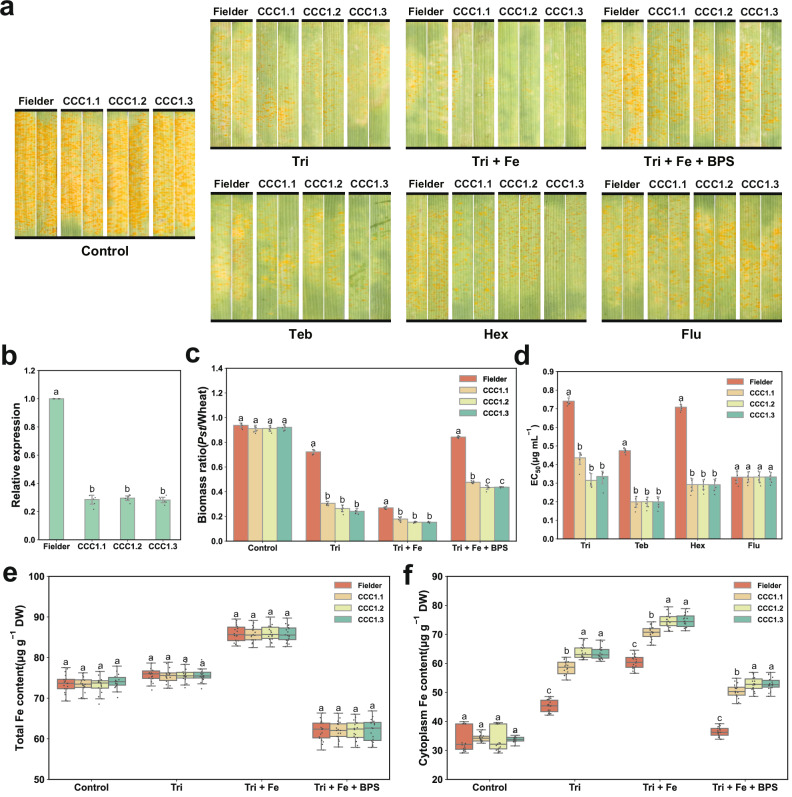
Silencing *PstCCC1.1*, *PstCCC1.2* and *PstCCC1.3* reduces triazole resistance in a low-resistant *Pst* isolate via disrupting cytosolic iron content. **a** Stripe rust phenotype of low resistant isolate YQ324 of *Puccinia striiformis* f. sp, *tritici* (*Pst*) on wheat plants (cv. Fielder) with RNAi-mediated silencing of *PstCCC1.1*, *PstCCC1.2* or *PstCCC1.3*. Detached leaves were treated for 7 days post-inoculation with 2.43 µg/mL triadimefon (Tri) alone, in combination with 1 mM FeSO₄ (Fe), or in combination with both Fe and 0.3 mM iron chelator bathophenanthroline disulfonate (BPS), as well as with other fungicides [2.43 µg/mL each of tebuconazole (Teb), hexaconazole (Hex) and flubeneteram (Flu)]. **b** Silencing efficiency of *PstCCC1.1*, *PstCCC1.2* or *PstCCC1.3* was measured at 48 hours post-inoculation with *Pst*, prior to the triadimefon treatment. Gene expression levels were normalized to the expression in *Pst*-inoculated Fielder plants. **c** Biomass quantification of *Pst* isolate YQ324 on RNAi plants under treatments with Tri, Tri + Fe, or Tri + Fe + BPS for 7 days. Elongation factor genes of wheat (*TaEF*) and *Pst* (*PstEF*) were used to normalize the RNA level of wheat leaves and *Pst*, respectively. **d** Comparison of the half-maximal effective concentration (EC₅₀) values of Tri, Teb, Hex, and Flu for *Pst* isolate YQ324 on the RNAi plants. Total iron (**e**) and cytosolic iron (**f**) content in *Pst* hyphae collected from RNAi plants treated with Tri, Tri + Fe, or Tri + Fe + BPS for 7 days, as determined using inductively coupled plasma mass spectrometry (ICP-MS). For box plots, boxes represent the interquartile range (25th to 75th percentiles), center lines indicate medians, and whiskers extend to 1.5 × IQR. Data in (**b**–**d**) are mean ± SD (*n* = 6 replicates). Data in (**e**, **f**) are mean ± SD (*n* = 18 replicates, each from 3 detached leaf segment). In (**b**–**f**), statistical significance was determined by one-way analysis of variance (ANOVA) with Duncan’s new multiple range test (two-sided). Different letters above the bars denote significant differences at *P* < 0.05. Source data are provided as a Source Data file.

To further confirm the involvement of the *PstCCC1* genes in triadimefon resistance, wheat lines were generated from variety Fielder transformed with the RNAi constructs targeting at the genes. After PCR screening for positive T₀ plants, we advanced them to produce homozygous T₃ seeds. The expression levels of *PstCCC1.1*, *PstCCC1.2* and *PstCCC1.3* in the low-resistance isolate YQ324 were reduced by 66.1–78.4% when the transgenic plants were inoculated with the isolate (Fig. 2b), and silencing each of the genes did not alter the expression of the other two genes (Supplementary Fig. 7b). As shown in Fig. 2a, the resistance of YQ324 to triazole fungicides [triadimefon (Tri), tebuconazole (Teb) and hexaconazole (Hex)] was significantly reduced on the transgenic RNAi wheat plants compared to the non-transgenic controls. After 7 days of the treatment with Tri, Tri + Fe or Tri + Fe + BPS, the *Pst* biomass on the RNAi transgenic lines was significantly lower than that on the untransformed control (Fig. 2c). Notably, the fungicide sensitivity was enhanced by the addition of exogenous iron (Fe) but was rescued by the iron chelator BPS. Additionally, EC_50_ measurements showed that gene silencing increased the sensitivity of the resistant isolate to the triazole fungicides triadimefon, tebuconazole and hexaconazole. In contrast, silencing did not affect the sensitivity to flubeneteram, which has a different mode of action, as shown by unchanged EC_50_ values (Fig. 2d).

To further determine whether the iron-dependent sensitivity was linked to alterations in cellular iron homeostasis, the total iron and cytosolic iron contents were determined using inductively coupled plasma mass spectrometry (ICP-MS). While the total iron content in the silenced isolates remained unchanged across all treatments (Fig. 2e), the cytosolic iron content was significantly elevated under the triadimefon stress. Analysis of key iron homeostasis genes revealed a coordinated transcriptional response to triadimefon treatment. The expression of iron acquisition genes (e.g., *sidA*, siderophore uptake components, and *ftrA* homologs) was generally suppressed. In contrast, genes involved in iron detoxification, particularly the vacuolar iron exporter *PstCCC1*, showed a marked upregulation (Supplementary Fig. 8). This pattern, characterized by reduced uptake coupled with enhanced detoxification capacity, indicates a state of cellular iron excess during triadimefon treatment. It aligns with the observed cytosolic iron overload despite unchanged total iron content and underscores a central role for *PstCCC1* in mediating this adaptive response. The cytosolic iron accumulation was further intensified by iron supplementation but reversed by the BPS chelation (Fig. 2f). These results confirmed that silencing *PstCCC1.1*, *PstCCC1.2* and *PstCCC1.3* through RNAi in wheat plants significantly reduced *Pst* resistance to the triazole fungicide, consistent with the transient silencing results, supporting the importance of these genes in mediating resistance to triazole fungicides. The results indicated that disrupted iron compartment, reflected by altered cytosolic iron levels rather than total content, drives triazole sensitivity. This mechanism is not applicable to fungicides with different modes of action, such as flubeneteram, a succinate dehydrogenase inhibitor (SDHI) fungicide.

### Moderate resistance to triadimefon is more dependent on ergosterol than low resistance

**Fig. 3:**
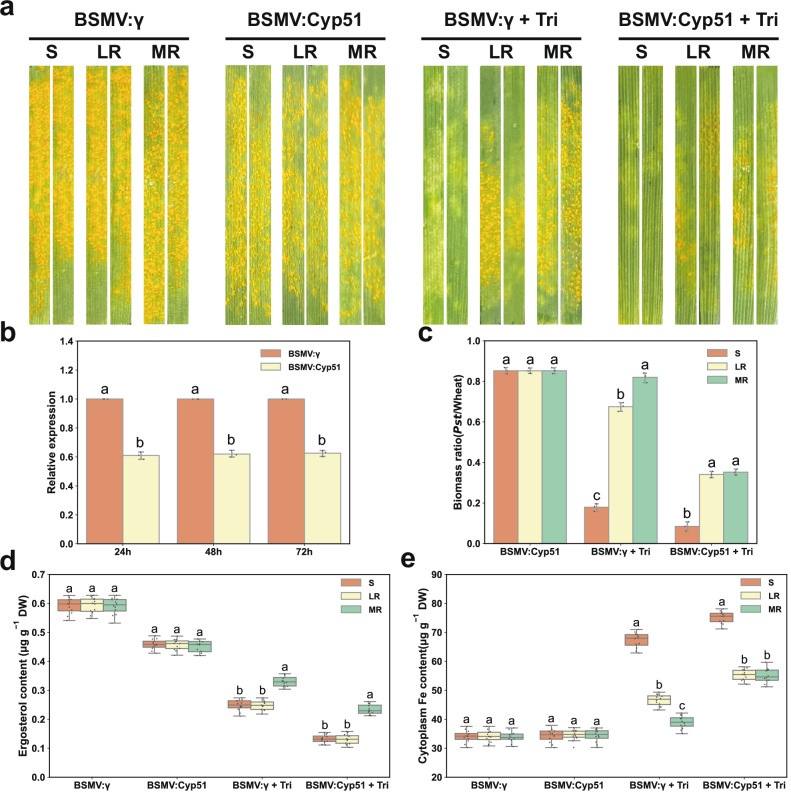
Cytosolic iron quantification and ergosterol-dependent response profiling in triadimefon-sensitive and resistant isolates of *Pst* post-treatment. **a** Phenotypic characterization of triadimefon (Tri) sensitivity in the sensitive (S), low resistant (LR), and moderately resistant (MR) isolates following silencing of *Cyp51*. **b** Silencing efficiency assay of *Cyp51* was measured in host-induced gene silencing (HIGS) plants at 24, 48, and 72 hours after *Pst* inoculation, prior to triadimefon treatment. The data were normalized to the expression level of wheat leaves inoculated with BSMV: γ after the inoculation with *Pst* sensitive isolate. **c** The biomass of sensitive (Gui1-2), low-resistant (YQ324) and moderately resistant (TS-7) isolates was assessed following inoculation onto non-silenced (BSMV: γ) or *Cyp51*-silenced (BSMV:*Cyp51*) wheat plants, with or without the subsequent Tri treatment (10 µg/mL). The biomass values were normalized to the respective non-silenced (BSMV: γ) and non-Tri control for each isolate. Concurrent analysis of ergosterol biosynthesis (**d**) and cytosolic iron content (**e**) in the *Pst* isolates following *Cyp51* silencing and triadimefon treatment. The experiment was conducted with sensitive (Gui1-2), low-resistant (YQ324) and moderately resistant (TS-7) isolates inoculated onto non-silenced (BSMV: γ) or *Cyp51*-silenced (BSMV:*Cyp51*) plants, with or without the Tri treatment. Ergosterol content and cytosolic iron levels in the hyphae were measured using high-performance liquid chromatography (HPLC) and inductively coupled plasma mass spectrometry (ICP-MS), respectively. For box plots, boxes represent the interquartile range (25th to 75th percentiles), center lines indicate medians, and whiskers extend to 1.5 × IQR. Data in (**b**, **c**) are mean ± SD (*n* = 3 replicates). Data in (**d**, **e**) are mean ± SD (*n* = 12 replicates, each from 3 detached leaf segment). In (**b**–**e**), statistical significance was determined by one-way analysis of variance (ANOVA) with Duncan’s new multiple range test (two-sided). Different letters above the bars denote significant differences at *P* < 0.05. Source data are provided as a Source Data file.

To elucidate the differences in cytoplasmic ferrous ion (Fe²⁺) content and ergosterol dependence among *Pst* isolates with different levels of resistant to triadimefon, we employed HIGS to transiently silence the target gene (*Cyp51*) of the DMI fungicide. Three isolates were used in this experiment, including a sensitive isolate (Gui 1-2, without mutations in the target gene *Cyp51* or the three *PstCCC1* genes), a low resistant isolate (YQ324, with mutations present in the three candidate genes), and a moderately resistant isolate (TS-7, collected from a wheat field in 2019 and carried the Y134F mutation in Cyp51). The isolates were used to inoculate susceptible wheat plants (cv. MX169), and their sensitivity was assessed following the triadimefon treatment (Fig. 3a). The transcription levels of *Cyp51* were reduced by 38.8–42.6% in the silenced plants (Fig. 3b). As shown in Fig. 3c, under non-silenced conditions without triadimefon treatment, the fungal biomass did not differ significantly among the three isolates. In contrast, biomass exhibited a gradient among the isolates under the triadimefon treatment in non-silenced plants, with the moderately resistant isolate showing the highest value, followed by the low-resistant and sensitive isolates. However, when *Cyp51* was partially silenced and triadimefon was applied, the biomass of both the moderately resistant and low-resistant isolates decreased to a similar level by the triadimefon treatment. The results indicate that the *Cyp51* expression contributes more substantially to triadimefon resistance in the moderately resistant isolate, while the dependence on *Cyp51* in the low resistant isolate was reduced.

Since *Cyp51* is a key gene in the fungal sterol biosynthesis pathway, we further quantified the ergosterol and cytosolic iron levels in the sensitive, low-resistant and moderately resistant isolates under the four combinatorial conditions of *Cyp51* silencing (non-silenced vs. silenced) and triadimefon treatment (non-treated vs. treated). As shown in Fig. 3d, ergosterol quantification revealed distinct dependencies on sterol biosynthesis among the isolates. In non-silenced *Cyp51* plants without triadimefon, the ergosterol levels of the three isolates were significantly different. However, following the triadimefon treatment in both non-silenced *Cyp51* and *Cyp51*-silenced isolates, a clear hierarchy in the ergosterol level was observed: moderately resistant > low-resistant > sensitive isolate. This graded response showed that ergosterol biosynthesis remained better protected in the moderately resistant isolate than the low-resistant and sensitive isolates under the triadimefon treatment. Combined with the biomass data, these results indicate that the low-resistant isolate possesses a significant ergosterol-independent resistance component, whereas the moderately resistant isolate relies more heavily on maintaining ergosterol biosynthesis for its resistance. In parallel, the analysis of cytosolic iron content (Fig. 3e) revealed a perfect inverse correlation with the ergosterol pattern. In the non-silenced *Cyp51* plants with the triadimefon treatment, cytosolic iron accumulation showed the progression: sensitive > low-resistant > moderately resistant isolates. This pattern was maintained under the *Cyp51*-silenced plus triadimefon conditions, where both moderately resistant and low-resistant isolates accumulated equally lower iron levels than the sensitive isolate. The consistent inverse relationship between cytosolic iron accumulation and fungicide resistance across all treatment conditions strongly suggests that intracellular iron compartmentalization serves as a key determinant of triadimefon sensitivity in *Pst*. These results indicate that the triadimefon treatment markedly elevated cytoplasmic iron levels in *Pst*. Notably, the resistance mechanism in the moderately resistant isolate exhibited greater dependence on ergosterol biosynthesis than the low-resistant isolate.

### Physiological and biochemical functions of *PstCCC1* genes and their mutants on triadimefon sensitivity

**Fig. 4:**
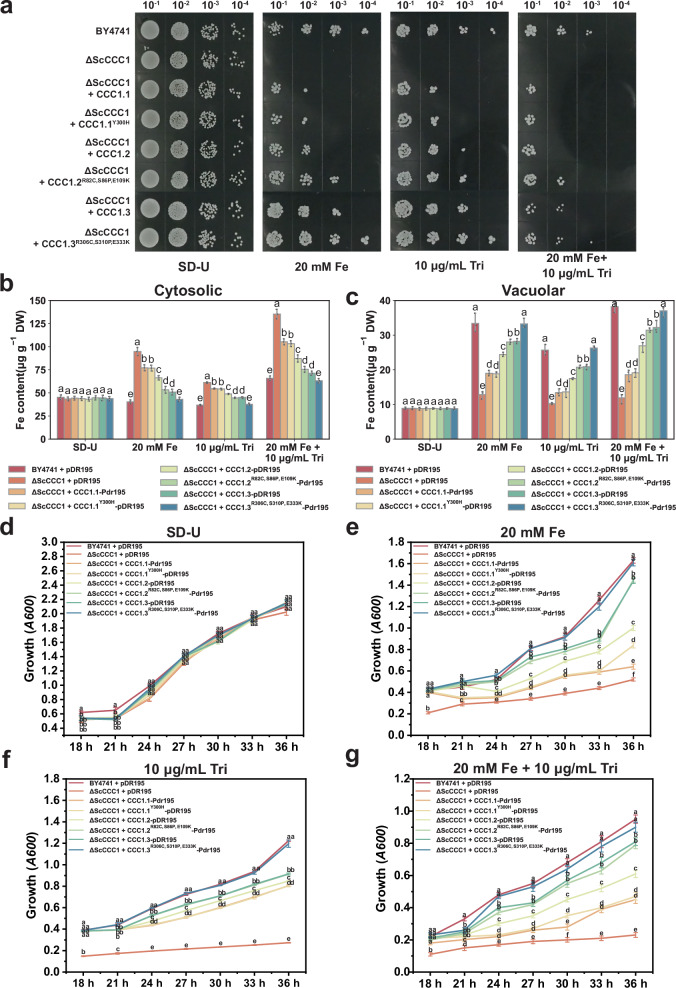
Functional analysis of candidate genes in *Pst* for triadimefon resistance and their effects of their mutants on Fe transport. **a** Functional analysis of candidate genes and their mutants in yeast. Growth of yeast cultures on the normal medium (SD-U, lacking Ura) and treatment media (SD-U + 20 mM FeSO_4_, SD-U + 10 μg/mg triadimefon and SD-U + 20 mM FeSO_4_ + 10 μg/mg triadimefon) for 2-3 days. Four 10-fold series of dilutions were made under sterile conditions. Analysis of Fe content in cytosolic (**b**) and vacuolar (**c**) on the SD-U and treatment media (SD-U + 20 mM FeSO_4_, SD-U + 10 μg/mg triadimefon and SD-U + 20 mM FeSO_4_ + 10 μg/mg triadimefon). Yeast growth curves monitored by OD₆₀₀ measurements under the following conditions: (**d**) SD-U medium from 18 to 36 hours post inoculation (hpi); (**e**) SD-U with 20 μM FeSO₄ from 18 to 36 hpi; (**f**) SD-U with 10 μg/mL triadimefon from 18 to 36 hpi; (**g**) SD-U with 20 μM FeSO₄ and 10 μg/mL triadimefon from 18 to 36 hpi. Data in (**b**–**g**) are mean ± SD (*n* = 3 replicates). In (**b**–**g**), statistical significance was determined by one-way analysis of variance (ANOVA) with Duncan’s new multiple range test (two-sided). Different letters above the bars denote significant differences at *P* < 0.05. Source data are provided as a Source Data file.

To assess the potential functional changes in the candidate genes *PstCCC1.1*, *PstCCC1.2*, *PstCCC1.3* and their mutants, a heterologous yeast complementation assay was conducted. The expression of the candidate genes and their green fluorescence protein (GFP) fusion mutants was analyzed in the yeast *CCC1* knockout mutant (ΔScCCC1), and fluorescence co-localization with the membrane-specific dye FM4-64 was used to visualize the vacuolar membrane (Supplementary Fig. 9). The coding sequences of the candidate genes and their mutants were cloned into the pDR195 vector and expressed in the ΔScCCC1 yeast mutant. The successfully transformed yeast cultures were spotted onto SD-U (Ura-deficient) medium and SD-U medium supplemented with 20 mM FeSO_4_ and 10 μg/mL triadimefon to evaluate the differences in iron transport and sensitivity to triadimefon. As shown in Fig. 4a, yeast expressing *PstCCC1.1*, *PstCCC1.2* and *PstCCC1.3*, along with their mutants, grew well and complemented the function of the ΔScCCC1 mutant. A hierarchy in iron stress tolerance was observed among the transformants: the *PstCCC1.3* showed the highest tolerance, followed by *PstCCC1.2* and *PstCCC1.1*. Notably, the mutants *PstCCC1.3*^*R306C,S310P,E333K*^ and *PstCCC1.2*^*R82C,S86P,E109K*^ showed greater tolerance to iron stress than their non-mutated counterparts, while the empty vector failed to complement this function. Under the triadimefon treatment (10 μg/mL) alone, the *PstCCC1.3* showed the highest triadimefon resistance, followed by that with *PstCCC1.2*, and the culture with *PstCCC1.1* showed the lowest resistance. The mutants *PstCCC1.3*^*R306C,S310P,E333K*^ and *PstCCC1.2*^*R82C,S86P,E109K*^ consistently exhibited higher tolerance than their non-mutated counterparts across all treatments, including triadimefon alone and the combination of both triadimefon and FeSO_4_.

As shown in Fig. 4b, the ΔScCCC1 mutant exhibited significantly higher iron content in cytosolic compared to other isolates. In the SD-U medium without supplements, no significant difference in iron content was observed between the non-mutated and mutant isolates. However, in the medium supplemented with 20 mM FeSO_4_, the iron content in the non-mutated and mutant isolates was lower than in that of ΔScCCC1. The iron content was ranked from the lowest to highest in the isolates with *PstCCC1.3*, *PstCCC1.2* and *PstCCC1.1*, and the isolates with *PstCCC1.3*^*R306C,S310P,E333K*^ and *PstCCC1.2*^*R82C,S86P,E109K*^ had lower iron contents than their non-mutated counterparts. Under the treatment of triadimefon (10 μg/mL) alone, the iron content in all transformed isolates was elevated compared to the SD-U control, with the *PstCCC1.3* and its mutant showing the lowest iron accumulation, followed by those of *PstCCC1.2* and *PstCCC1.1*. The same hierarchy was observed under FeSO₄ or combined FeSO₄ and triadimefon stresses, confirming that the mutants retained a superior capacity to limit cytosolic iron accumulation. Analysis of vacuolar iron content (Fig. 4c) revealed an inverse pattern relative to cytosolic iron across treatments. Exception for the SD-U control, all transformants showed higher vacuolar iron under the treatment of triadimefon, FeSO₄, or both. The mutants *PstCCC1.3*^*R306C,S310P,E333K*^ and *PstCCC1.2*^*R82C,S86P,E109K*^ exhibited greater vacuolar iron sequestration than their non-mutated versions, reinforcing their role in iron compartmentalization.

Yeast growth curves under non-stress conditions (Fig. 4d) showed no observable differences among isolates. In contrast, under 20 mM FeSO₄ (Fig. 4e), 10 μg/mL triadimefon alone (Fig. 4f), or both (Fig. 4g), growth inhibition was remarkably more severe in the ΔScCCC1 mutant. The inhibition of the transformants expressing *PstCCC1.3* and its mutant was the lowest, followed by those of *PstCCC1.2* and *PstCCC1.1*. The same tolerance hierarchy was observed across all treatments, with the combined FeSO₄ + triadimefon condition inflicting the highest inhibition. Collectively, these results demonstrate that the *PstCCC1* genes contribute to triadimefon resistance even in the absence of iron supplementation, with the mutant alleles conferring enhanced tolerance under both triadimefon-alone and triadimefon-iron combined stresses. This functional superiority is linked to the ability to reduce cytosolic iron via enhanced vacuolar sequestration.

### Triadimefon treatment induces iron-amplified oxidative damage in fungal pathogens through reactive oxygen species (ROS) accumulation and lipid peroxidation

The observed accumulation of cytoplasmic Fe²⁺ suggested that triadimefon disrupts fungal iron homeostasis, potentially contributing to its fungicidal activity through Fenton chemistry-mediated oxidative stress. To investigate the effects of triadimefon treatment on iron homeostasis and oxidative stress in *Pst*, we analyzed the expression patterns of genes related to iron-dependent cell death and lipid peroxidation. Totally, 175 genes involved in cell death were identified in *Pst* based on their protein homology and conserved domain annotation (Supplementary Fig. 10a). As shown in Supplementary Fig. 10a, most genes promoting cell death were upregulated in *Pst* following the triadimefon treatment, especially *CAMK2B*, *Hog1* and *SLC7A11*^26,27^, whereas most cell death inhibitory genes represented by *FET* and *MBOAT1*^28,29^, were down-regulated by triadimefon. Thus, these results suggested that the triadimefon treatment might promote the cell death of *Pst* to exert its inhibitory effects. Furthermore, the topological coexpression results showed that expressions of *PstCCC1.1*, *PstCCC1.1* and *PstCCC1.3* were negatively correlated with plenty of cell death promoting genes (Supplementary Fig. 10b), implying its function in alleviating cell death associated with Fe accumulation in *Pst* caused by the triadimefon treatment.

**Fig. 5:**
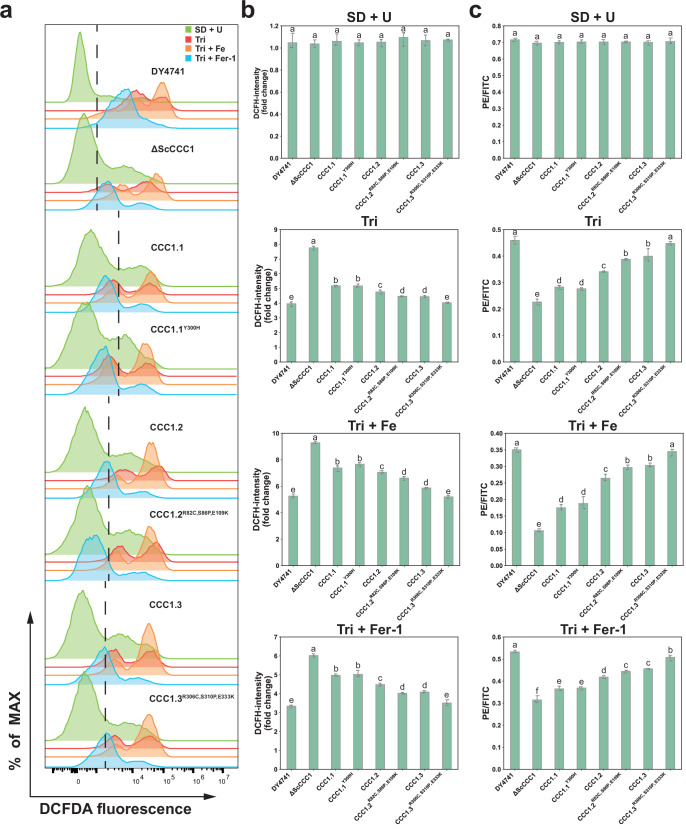
Reactive oxygen species (ROS) and lipid peroxidation assay in the yeast transformed with candidate genes for triadimefon resistance under triadimefon/Fe²⁺/Ferrostatin-1 (Fer-1) treatments. **a**–**b** ROS levels in transgenic yeast cells with candidate genes under triadimefon/Fe²⁺/Fer-1 treatments detected through flow cytometry. The DCFH fluorescence intensity is positively correlated with the intracellular ROS levels. **c** The levels of lipid peroxide were detected through staining with C11-BODIPY probe after different treatments. Phycoerythrin (PE, excitation/emission: 480/578 nm) and fluorescein isothiocyanate (FITC, excitation/emission: 494/518 nm) exhibit red and green fluorescence, respectively. The progressive shift from PE (red) to FITC (green) fluorescence signals lipid peroxidation progression, with decreased PE/FITC ratios quantitatively reflecting increased peroxidation levels. Data in (**b**) and (**c**) are presented as mean ± standard deviation (*n* = 3 replicates). Different letters indicate significant differences as determined by one-way analysis of variance (ANOVA) with Duncan’s new multiple range test (two-sided) (*P* < 0.05) in (**b**) and (**c**). Source data are provided as a Source Data file.

As we know, an iron- and triadimefon-dependent form of oxidative cell death is characterized by production of ROS and subsequent lipid peroxidation^30,31^. Consistently, transcriptome results showed that the triadimefon treatment triggered the expression of genes encoding antioxidant enzymes, supporting that triadimefon could induce oxidative stress in *Pst* (Supplementary Fig. 11). To assess the differential responses to the triadimefon treatment among yeast heterologous transformants of *PstCCC1.1*, *PstCCC1.2*, *PstCCC1.3* and their mutants, we investigated ROS generation and lipid peroxidation under triadimefon treatment. As shown in Fig. 5a, compared to the control, triadimefon significantly increased ROS accumulation in all yeast strains. This effect was significantly alleviated by the addition of Ferrostatin-1 (Fer-1), an inhibitor of iron-dependent oxidative death. However, the co-treatment with triadimefon and ferrous iron (Fe²⁺) synergistically exacerbated ROS accumulation. Notably, the yeast mutant ΔScCCC1 exhibited the most pronounced ROS accumulation among all tested isolates. Furthermore, in heterologous transformants of *PstCCC1.1*, *PstCCC1.2* and *PstCCC1.3* and their corresponding mutants, the *PstCCC1.2*^*R82C,S86P,E109K*^ and *PstCCC1.3*^*R306C,S310P,E333K*^ mutants showed significantly reduced ROS accumulation compared their non-mutated gene counterparts, as demonstrated by markedly decreased DCFH fluorescence intensity, while *PstCCC1.1* transformants maintained similar ROS levels to the BY4741 (Fig. 5b). C11-BODIPY staining showed that the triadimefon treatment significantly enhanced lipid peroxidation levels in the BY4741 and all yeast transformant isolates. This enhancement was effectively mitigated by the Fer-1 treatment, whereas co-application of triadimefon with Fe²⁺ synergistically exacerbated lipid peroxidation. Notably, the ΔScCCC1 mutant exhibited the most severe lipid peroxidation among all tested cultures. Furthermore, in heterologous transformants of *PstCCC1.2* and *PstCCC1.3*, the mutant alleles showed significantly reduced lipid peroxidation compared to their non-mutated gene counterparts, as evidenced by markedly increased PE/FITC ratios, while the *PstCCC1.1* transformants maintained similar peroxidation levels to the BY4741 (Fig. 5c). These findings demonstrated that the triadimefon treatment induced an iron-dependent oxidative cell death in *Pst* through iron-mediated mechanisms. Notably, when the iron transport activity of resistance-associated mutant alleles (*PstCCC1.2*^*R82C,S86P,E109K*^ and *PstCCC1.3*^*R306C,S310P,E333K*^) were associated with reduced cytosolic iron content and the lower levels of iron-mediated oxidative damage, thereby contributing to their reduced sensitivity to triadimefon.

### Heterologous overexpression of *PstCCC1* genes and their mutants in *Fusarium graminearum* ΔCCC1 mutant (ΔFgCCC1) for determining triadimefon sensitivity

To further elucidate the function of the *PstCCC1* genes and the impact of their mutations on triadimefon sensitivity and iron homeostasis, we conducted a heterologous expression experiment in the ΔFgCCC1 mutant. The coding sequences of *PstCCC1.1*, *PstCCC1.2*, *PstCCC1.3* and their mutants were expressed in ΔFgCCC1, and then the transformants were subjected to phenotypic, sensitivity, and ion content analyses. The GFP fusion proteins of these candidate genes and their mutants co-localized with the vacuole-specific dye CMAC in the vacuoles (Supplementary Fig. 12). As shown in Supplementary Fig. 13a, overexpression of all three non-mutant *PstCCC1* genes partially restored the growth of the ΔFgCCC1 mutant on triadimefon-containing media. The recovery efficacy varied, ranking from the highest to lowest in the order of *PstCCC1.3*, *PstCCC1.2* and *PstCCC1.1*. Notably, the mutant alleles *PstCCC1.3*^*R306C,S310P,E333K*^ and *PstCCC1.2*^*R82C,S86P,E109K*^ conferred more robust restoration of growth under the triadimefon treatment than their non-mutant counterparts, suggesting a gain-of-function phenotype. The simultaneous application of iron (FeSO₄) and triadimefon exacerbated the growth inhibition across all cultures, leading to more severe suppression than the triadimefon treatment alone. Despite this enhanced toxicity, the rank order of recovery efficacy among the overexpressed genes (*PstCCC1.3* > *PstCCC1.2* > *PstCCC1.1*) was maintained, with the mutant alleles of *PstCCC1.3* and *PstCCC1.2* consistently conferring higher recovery than their non-mutant counterparts.

Consistent with the growth assays, quantification of the inhibition rate revealed that the cytoprotective effect of *PstCCC1* overexpression was dependent on both the gene and the presence of iron. While the addition of FeSO₄ alone had a little effect, it exacerbated the growth inhibition caused by triadimefon in all cultures. Conversely, the iron chelator BPS significantly alleviated triadimefon-induced inhibition. Across all these treatment conditions, cultures overexpressing the mutant alleles of *PstCCC1.3* and *PstCCC1*.2 consistently exhibited the lowest inhibition rate, outperforming their non-mutant counterparts and confirming the enhanced function provided by these mutations (Supplementary Fig. 13b).

We expanded the sensitivity profiling to include other fungicides. The determination of EC₅₀ values revealed that the *PstCCC1* genes and their mutants specifically affected sensitivity to DMI fungicides, namely triadimefon, tebuconazole and hexaconazole. The sensitivity trend mirrored the phenotypic recovery: cultures expressing *PstCCC1.3* and its mutant were the least sensitive, followed by *PstCCC1.2* and *PstCCC1.1*, with the mutants consistently exhibiting lower sensitivity than the non-mutant genes (Supplementary Fig. 13c). In contrast, the EC₅₀ of flubeneteram, an SDHI fungicide with a different mode of action, remained unchanged across all cultures. This indicates that the resistance mechanism conferred by the *PstCCC1* mutations, mediated through altering cytosolic iron homeostasis, is specific to DMI fungicides.

To directly investigate the iron homeostasis mechanism, we measured intracellular iron content. While the total iron content remained largely unchanged across cultures under different conditions (Supplementary Fig. 13d), profound differences were observed in the cytosolic iron fraction, especially the ΔFgCCC1 mutant accumulated the highest level of cytosolic iron (Supplementary Fig. 13e). All *PstCCC1*-overexpressing isolates showed a reduced cytosolic iron pool compared to ΔFgCCC1, with the levels ranking from lowest to highest as *PstCCC1.3*, *PstCCC1.2* and *PstCCC1.1*. Importantly, the mutant alleles *PstCCC1.3*^*R306C,S310P,E333K*^ and *PstCCC1.2*^*R82C,S86P,E109K*^ led to even lower cytosolic iron than their non-mutated counterparts. This inverse correlation between cytosolic iron content and triadimefon sensitivity firmly established a link between iron partitioning and DMI fungicide resistance. The iron accumulation phenotype was independently confirmed using the Fe-selective fluorescent dye FeRhoNox-1. The fluorescence microscopy analysis (Supplementary Fig. 13f) visually demonstrated that the ΔFgCCC1 mutant had the highest whole-cell iron signal, which was significantly reduced in the *PstCCC1*-overexpressing cultures, validating the quantitative data from ICP-MS. In summary, these results demonstrate that the *PstCCC1* genes mediate vacuolar iron sequestration, thereby influencing DMI fungicide sensitivity. The resistance-conferring mutations enhance this process, resulting in a more pronounced reduction of the cytosolic iron pool and a corresponding decrease in sensitivity to DMI fungicides.

### The proteins of *PstCCC1* mutants had enhanced iron-ion binding affinity

**Fig. 6:**
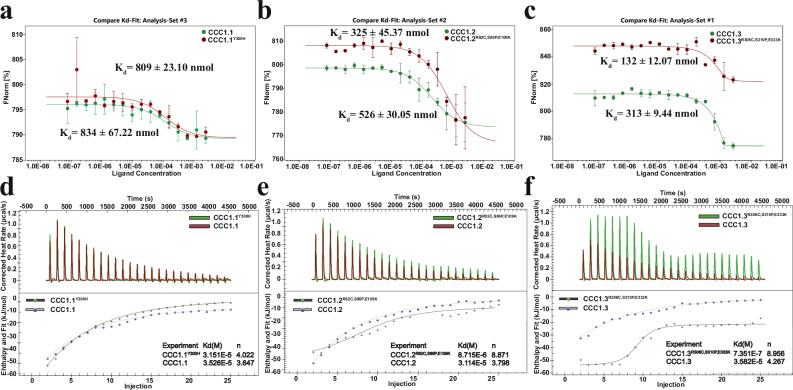
Microscale thermophoresis (MST) and isothermal titration calorimetry (ITC) analyses for binding interactions of PstCCC1 and their mutants with FeSO_4_. **a** PstCCC1.1 and PstCCC1.1^Y300H^, (**b**) PstCCC1.2 and PstCCC1.2^R82C,S86P,E109K^ and **c** PstCCC1.3 and PstCCC1.3^R306C,S310P,E333K^ binding with FeSO_4_ were assessed through MST. The binding curves fit the data points to the standard K_d_-fit function. Mean and standard deviations were calculated with the data from three independent replicates. **d** PstCCC1.1 and PstCCC1.1^Y300H^, (**e**) PstCCC1.2 and PstCCC1.2^R82C,S86P,E109K^, (**f**) PstCCC1.3 and PstCCC1.3^R306C,S310P,E333K^ binding with FeSO_4_ were assessed through ITC. K_d_, the dissociation constant. n, stoichiometric ratio.

To assess the iron-ion binding and affinities of the PstCCC1 proteins, molecular docking analysis was conducted. PstCCC1.1 and PstCCC1.1^Y300H^ primarily bound iron ions via hydrogen bonds with ASP141, with the ΔiG values of −2.4 kcal/mol and −3.3 kcal/mol, respectively. PstCCC1.2 and PstCCC1.2^R82C,S86P,E109K^ bound iron ions via hydrogen bonds with TYR94, with the ΔiG values of −4.5 kcal/mol and −7.8 kcal/mol, respectively. PstCCC1.3 formed a single hydrogen bond with ASP141, yielding a binding energy of −6.5 kcal/mol, while the mutant PstCCC1.3^R306C,S310P,E333K^ formed two hydrogen bonds (with ASP141 and MET177), exhibiting the highest binding energy of -8.3 kcal/mol (Supplementary Fig. 14). To further validate these results, we measured the binding affinities of these proteins for iron ions using Microscale Thermophoresis (MST) and Isothermal Titration Calorimetry (ITC). The purified proteins with the target band sizes confirmed through SDS-PAGE were used for further affinity analysis (Supplementary Fig. 15). The MST results revealed that PstCCC1.1 and PstCCC1.1^Y300H^ bound iron ions, with dissociation constants (K_d_) of 809 nM and 834 nM, respectively (Fig. 6a). PstCCC1.2 and PstCCC1.2^R82C,S86P,E109K^ also bound iron ions, with K_d_ values of 526 nM and 325 nM, respectively (Fig. 6b). Notably, PstCCC1.3^R306C,S310P,E333K^ showed a significantly higher dissociation constant for iron ions than PstCCC1.3, with the K_d_ values of 313 nM and 132 nM, respectively (Fig. 6c). Additionally, ITC measurements confirmed these findings, showing that the proteins of all three candidate genes and their mutants can bind iron ions, with varying dissociation constants. Except for PstCCC1.1^Y300H^, the binding affinities of PstCCC1.2^R82C,S86P,E109K^ and PstCCC1.3^R306C,S310P,E333K^ were significantly higher than those of their non-mutated counterparts. The ITC analysis also provided the stoichiometric ratio (n), which reflects the number of iron ions bound per protein molecule. As shown in Fig. 6d, PstCCC1.1 and PstCCC1.1^Y300H^ bound iron ions with similar dissociation constants and stoichiometric ratios. However, PstCCC1.2^R82C,S86P,E109K^ exhibited a significantly higher binding affinity (K_d_ = 8.7 μM, *n* = 8.9) and stoichiometric ratio than PstCCC1.2 (K_d_ = 31.1 μM, *n* = 3.8) (Fig. 6e). Similarly, PstCCC1.3^R306C,S310P,E333K^ showed a significantly higher affinity for iron ions (K_d_ = 0.7 μM, *n* = 9.0) than PstCCC1.3 (K_d_ = 35.8 μM, *n* = 4.2) (Fig. 6f).

We then predicted the structures of the vacuolar iron transporters of the PstCCC1 and their mutant proteins. Overall, the structure of the PstCCC1.1 was similar to that of the PstCCC1.1^Y300H^ (rmsd: 2.3 Å) (Supplementary Fig. 16a), whereas the structures of the PstCCC1.2^R82C,S86P,E109K^ and PstCCC1.3^R306C,S310P,E333K^ had notable changes from PstCCC1.2 and PstCCC1.3 with rmsd=3.2 Å and 4.3, respectively) (Supplementary Fig. 16b–c). Furthermore, yeast two-hybrid and split luciferase complementation assays identified an interaction between PstCCC1.1 and PstCCC1.2 (Supplementary Fig. 17a–b). Molecular docking simulations revealed that their heterodimer exhibits a stronger ferrous iron (Fe²⁺) binding affinity than either protein alone (Supplementary Fig. 17c). These results suggest that the original proteins and their mutant versions of the three PstCCC1 can bind iron ions, albeit with notable differences in binding affinity and kinetics. The mutants PstCCC1.2^R82C,S86P,E109K^ and PstCCC1.3^R306C,S310P,E333K^ showed significantly higher dissociation constants and stoichiometric ratios than their corresponding wild types. Consistent with the functional phenotypes observed in vivo, these biophysical data support the notion that the mutations may enhance iron binding affinity, potentially contributing to triadimefon resistance.

### The triadimefon resistance mechanism mediated by specific amino acid mutations in the PstCCC1 proteins of *Pst* is conserved in *Fg*

**Fig. 7:**
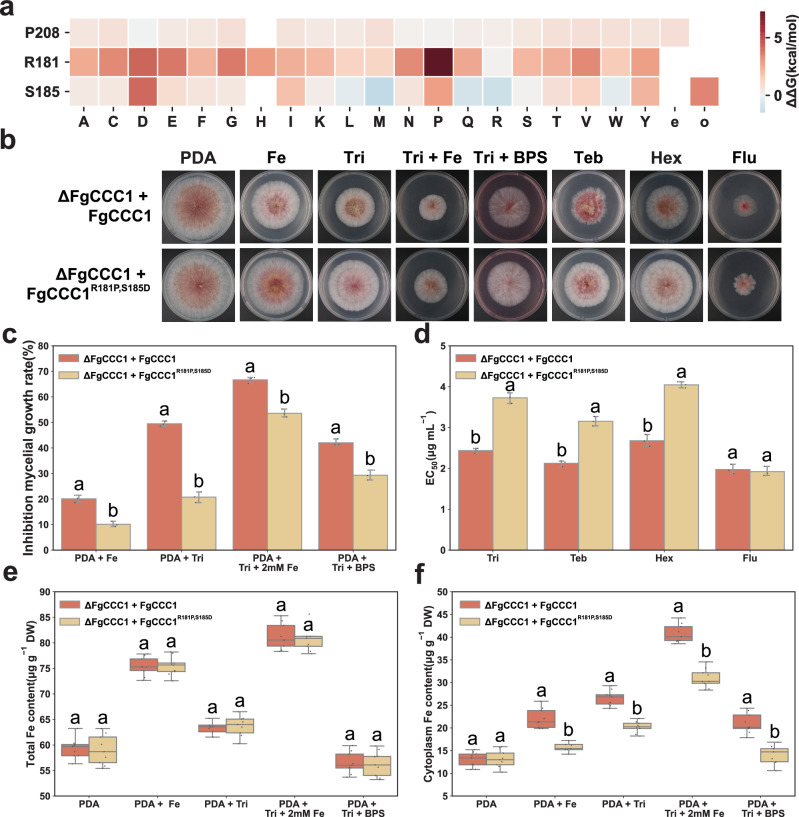
Conservation validation of the CCC1-mediated triadimefon resistance mechanism from *Pst* in *Fusarium graminearum* (Fg). **a** Prediction of protein structural impacts via virtual saturation mutagenesis (VSM) of three resistance-related mutation sites identified via homologous alignment between PstCCC1.3 and FgCCC1. The table is color-coded by correlation based on the color legend. **b** Phenotypic analysis of *Fg* cultures grown for 3 days on potato dextrose agar (PDA) under different conditions: normal PDA, PDA supplemented with 2 mM FeSO₄, PDA with 2.5 µg/mL triadimefon (Tri), PDA with 2 mM FeSO₄ and 2.5 µg/mL Tri, PDA with 2.5 µg/mL Tri and 0.1 mM BPS, PDA with 2.5 µg/mL Teb, PDA with 2.5 µg/mL Hex, and PDA with 2.5 µg/mL Flu. Strains include the complemented wild-type control (ΔFgCCC1 + FgCCC1) and the mutant (ΔFgCCC1 + FgCCC1^R181P,S185D^). **c** Inhibition mycelial growth rates (%) of mycelial growth under four treatment conditions: PDA + 2 mM FeSO₄, PDA + 2.5 µg/mL Tri, PDA + 2 mM FeSO₄ + 2.5 µg/mL Tri and PDA + 2.5 µg/mL Tri + 0.1 mM BPS. Inhibition rates were calculated in relative to the mycelial diameter of the untreated control for each strain. **d** Comparison of EC₅₀ values for Tri, Teb, Hex, and Flu between the control (ΔFgCCC1 + FgCCC1) and mutant (ΔFgCCC1 + FgCCC1^R181P,S185D^) cultures. Total iron (**e**) and cytosolic iron (**f**) content measured by inductively coupled plasma mass spectrometry (ICP-MS) in mycelia grown under four conditions: normal PDA, PDA + 2 mM FeSO₄, PDA + 2.5 μg/mL Tri, and PDA + 2 mM FeSO₄+2.5 μg/mL Tri. For box plots, boxes represent the interquartile range (25th to 75th percentiles), center lines indicate medians, and whiskers extend to 1.5 × IQR. Data in (**c**), (**d**) are mean ± SD (*n* = 3 replicates). Data in (**e**), (**f**) are mean ± SD (*n* = 9 replicates, each with mycelia pooled from 3 petri dishes). In (**c**–**f**), statistical significance was determined by one-way analysis of variance (ANOVA) with Duncan’s new multiple range test (two-sided). Different letters above the bars denote significant differences at *P* < 0.05. Source data are provided as a Source Data file.

To further determine whether the triadimefon resistance mechanism mediated by specific amino acid mutations in the PstCCC1.1 protein is conserved between the *Basidiomycete* fungus (*Pst*) and the *Ascomycete* fungus (*Fg*), we initially determined whether the amino acid mutation sites in PstCCC1.1, PstCCC1.2 and PstCCC1.3 are conserved in *Fg* and yeast fungus *Saccharomyces cerevisiae* (*Sc*) through the multiple sequence alignment. The results demonstrated that, besides PstCCC1.1, the key resistance-associated mutation sites R82 and S86 in PstCCC1.2, as well as R306 and S310 in PstCCC1.3, were also conserved in both *Fg* and *Sc* (Supplementary Fig. 18). Furthermore, saturation mutagenesis of the Vina-predicted binding sites in FgCCC1 was conducted using FoldX to evaluate the effects of mutations on protein stability and binding affinity. As shown in Fig. 7a, the triadimefon resistance mechanism mediated by specific point mutations in PstCCC1 of *Pst* was conserved, as confirmed by functional validation at corresponding amino acid sites in *Fg*. The substitution of amino acid residue 208 with any other amino acid contributed minimally to protein structural changes. In contrast, the R181P and S185D mutations induced the most significant conformational alterations in the protein structure. We therefore generated a mutant allele, FgCCC1^R181P,S185D^, and introduced it into the ΔFgCCC1 knockout mutant for functional comparison with the non-mutant gene complemented (ΔFgCCC1 + FgCCC1). Phenotypic analysis on various media revealed that the ΔFgCCC1 + FgCCC1^R181P,S185D^ exhibited significantly reduced sensitivity to triadimefon compared to the control culture under all tested conditions, including on media supplemented with triadimefon alone or in combination with FeSO₄ or the iron chelator BPS (Fig. 7b). Quantification of the growth inhibition confirmed that the mutant was significantly less inhibited than the control under all treatments (Fig. 7c). We further assessed the sensitivity spectrum by determining the EC₅₀ values for several fungicides. As shown in Fig. 7d, the FgCCC1^R181P,S185D^ mutant showed significantly higher EC₅₀ values to the demethylation inhibitor (DMI) fungicides triadimefon, tebuconazole and hexaconazole than to the control culture. In contrast to the elevated EC₅₀ values for DMI fungicides, the EC₅₀ for the SDHI fungicide flubeneteram was unaltered in the mutant, thereby demonstrating the specificity of the FgCCC1-mediated resistance. To investigate the biochemical basis of the resistance, we measured intracellular iron content. While the total iron content remained largely unchanged between the mutant and control isolates under various conditions (Fig. 7e), profound differences were observed in the cytosolic iron content (Fig. 7f). The mutant isolate ΔFgCCC1 + FgCCC1^R181P,S185D^ consistently maintained a significantly lower level of cytosolic iron than the control isolate ΔFgCCC1 + FgCCC1 across all treatments, including Tri, Tri+Fe, and Tri+Fe+BPS.

Collectively, these results confirm that the point mutations in FgCCC1, homologous to those conferring resistance in *Pst*, enhance DMI fungicide resistance without altering total cellular iron. This is achieved by modulating intracellular iron partitioning, specifically by reducing iron accumulation in the cytosolic pool, thereby mitigating the iron-mediated toxicity potentially produced by DMI fungicides. This mechanism represents a conserved adaptive strategy in fungi.

### Detection of PstCCC1 mutation sites in triadimefon-resistant and sensitive isolates

To further investigate the correlation between mutation sites in the *PstCCC1* genes and the triadimefon sensitivity of *Pst*, we conducted sequencing analysis on 33 isolates selected from 486 *Pst* isolates used in the previous study^8^. The 33 resistant isolates were classified as 17 moderately resistant isolates (EC_50_: 1.97–6.65 μg/mL), 16 low-resistant isolates (EC_50_ = 1.01–1.82 μg/mL) and 5 sensitive isolates (EC_50_ = 0.08–0.40 μg/mL). As shown in Table 1, no mutations of the three *PstCCC1* genes were detected in the 5 sensitive isolates. However, mutations in different regions of *PstCCC1.2* and *PstCCC1.3* were found in 6 of the 16 low resistant isolates, suggesting that these point mutations may contribute to the low resistance of *Pst* to triadimefon. No Y300H mutation was observed in PstCCC1.1, indicating that this mutation is not associated with triadimefon resistance in *Pst*. Additionally, the mutation sites R82C, S86P, E109K, R306C, S310P and E333K located in PstCCC1.2 or PstCCC1.3 were frequently found in the low resistant isolates. Notably, the E333K mutation was present in all six low resistant isolates with mutations, strongly linking this mutation to triadimefon resistance. Ten low resistant isolates did not show any mutations in the three *PstCCC1* genes or the previously tested *Cyp51* gene, suggesting the involvement of a third resistance mechanism.

**Table 1:** Presence of the demethylation inhibitor target enzyme Cyp51 mutation and mutations in ion transport proteins (PstCCC1.1, PstCCC1.2 and PstCCC1.3) identified from the sexual segregating population of *Puccinia striiformis* f. sp *tritici* (*Pst*) in 38 isolates selected from the 2019 collection in China and their ranges of half maximal effective concentration (EC50), resistance index (RI) and classes of triadimefon sensitivity

Mutation	No. (isolates)^a^	EC_50_ (μg/mL)	RI	Sensitivity^b^
No mutation	5 (SaX-17, HeB-6, HN-9, SC-24, GS-5)	0.08–0.40	0.41–2.09	S
Cyp51^Y134F^	17 (QH-42, QH-32, GS-44, GS-8, HuB-23, HuB-11, SC-69, SC-65, SC-58, SC-47, SaX-84, SaX-76, SaX-67, SaX-65, SaX-28, SaX-22, SaX-7)	10.35–34.99	1.97–6.65	MR
PstCCC1.1^Y300H^	0	NA^c^	NA	NA
PstCCC1.2 ^R82C^	3 (SaX-73, HeB-3, GS-28)	1.05–1.46	5.51–7.70	LR
PstCCC1.2 ^S86P^	0	NA	NA	NA
PstCCC1.2^E109K^	4 (SaX-73, HeB-3, SC-12, GS-28)	1.05–1.65	5.51–8.68	LR
PstCCC1.3^R306C^	5 (SaX-73, HeB-3, SC-34, GS-28, QH-38)	1.05–1.66	5.51–8.73	LR
PstCCC1.3^S310P^	1 (SC-12)	1.65	8.68	LR
PstCCC1.3^E333K^	6 (SaX-73, HeB-3, SC-12, SC-34, GS-28, QH-38)	1.05–1.66	5.51–8.73	LR

^c^*NA* = not applicable as none of the isolates having the mutation.

^a^The 38 isolates were selected from the natural collection in 2019^8^, including 5 sensitive and 33 resistant (LR and MR) isolates to triadimefon.

^b^S = sensitive, MR = moderately resistant and LR = low resistant.

## Discussion

The limited availability of drug targets for antifungal design makes fungal diseases in humans, animals and plants particularly difficult to control. Compounding this challenge is the growing issue of drug resistance, which further exacerbates the problem. While mostly known resistance mechanisms in plant pathogens to DMI fungicides are closely linked to their target gene *Cyp51*, our understanding of how plant pathogens respond to DMI-induced stress remains limited. For example, Kang et al. observed that after triadimefon treatment, *Pst* protoplasts displayed an increase in both the number and volume of vacuoles, which nearly occupied most of the haustorial space^32,33^. Yet, the content and regulatory mechanism underlying the vacuolar expansion remained to be elucidated. Given that DMI fungicides target key components of cell membranes, their effects are broad and complex. In the present study, we identified a previously unknown mechanism of DMI fungicide resistance in a major plant pathogen. We provide evidence that specific amino acid substitutions (PstCCC1.2^R82C,S86P,E109K^ and PstCCC1.3^R306C,S310P,E333K^) in vacuolar iron transporters confer triadimefon resistance in *Pst* by enhancing iron detoxification. This represents a clear case of non-target site resistance that is genetically distinct from the well-characterized *Cyp51*-based mechanism.

The genomic architecture of the *PstCCC1* region reveals key evolutionary and functional adaptations. The clustered organization of *PstCCC1.1*, *PstCCC1.2* and *PstCCC1.3* within a narrow region on Chrom6 points to a local gene amplification event, in contrast to the distant location of the fourth homolog. Functional specialization of this region is further evidenced by significant enrichment of iron transport-related GO terms. We propose that this amplification originated as an evolutionary response to iron stress, likely triggered by iron-withholding defense mechanisms in wheat and other *Poaceae* hosts, rather than by modern fungicide exposure. This pre-existing iron detoxification system appears to have been co-opted for triadimefon resistance. Typically, the function of transporters was mainly determined by the transmembrane and conformation structure^34^. We noted that the natural variations in key residues of PstCCC1 also altered the conformation structure, which might cause variations in function and transmembrane domains among the PstCCC1 variants, particularly in the number of transmembrane domains and mutation-induced extracellular peptide extensions (Supplementary Fig. 19). Notably, the elongated extracellular segment in the PstCCC1.3^R306C,S310P,E333K^ mutant correlates with its superior iron-binding and transport efficiency, suggesting that conformational optimization through specific amino acid substitutions enhances protein function. Moreover, the region responsible for binding iron ions mainly distributed in the elongated extracellular segment of the PstCCC1.3^R306C,S310P,E333K^ mutant, implying that the PstCCC1.3^R306C,S310P,E333K^ mutant could capture iron ions more efficiently in *Pst* under the triadimefon treatment. This mutation-associated changes in extracellular segment of PstCCC1.3^R306C,S310P,E333K^ effectively explained the enhanced functions of PstCCC1.3^R306C,S310P,E333K^ in transporting iron ions in *Pst* following the triadimefon treatment. Moreover, this structural refinement, consistent with established mechanisms where point mutations alter protein conformation and activity^34^, positions PstCCC1.3^R306C,S310P,E333K^ as the most functionally optimized variant in the triadimefon resistance mechanism.

The resistant isolates treated with both triadimefon and iron ions exhibited significantly increased sensitivity compared to the triadimefon treatment alone, supporting the hypothesis that the *Pst* sensitivity to triadimefon correlates with the intracellular iron ion level. We propose three mechanisms for the observed increase in intracellular iron ions under triadimefon stress: (1) DMI fungicides, including triadimefon, inhibit key enzymes in the ergosterol synthesis pathway, altering cell membrane fluidity and permeability. Studies have shown that similar DMI fungicides, such as fluconazole, affect vacuolar hydrogen ion ATPase in *Candida albicans*, leading to cytoplasmic calcium imbalance and severe growth inhibition, which can be restored with exogenous ergosterol^15^. DMI fungicides that affect calcium homeostasis, like amiodarone, are used with triazole agents to treat resistant strains in clinical settings^16,35^. Based on this finding, we hypothesize that triadimefon increases intracellular iron ion content by disrupting vacuolar hydrogen ion ATPase, thus enhancing sensitivity to combined triadimefon and iron ion stress. (2) DMI fungicides bind to the heme iron active center of 14-α demethylase (Cyp51), interrupting ergosterol synthesis. The sequestration of this central iron atom and the ensuing metabolic disruption are intrinsically linked to a broader dysregulation of cellular iron homeostasis^36^. (3) Studies have also shown that transcription factors, such as HapX in *Aspergillus*, can regulate both iron transporters like CCC1 and DMI fungicide target enzymes like Cyp51, positively mediating these genes’ expression under fungicide stress^37,38^. This dual regulatory role suggests that fungal iron homeostasis maintenance necessitates concomitant adjustment of ergosterol production. In summary, DMI fungicides may disrupt iron homeostasis in fungal pathogens, with vacuolar iron transporters’ efficiency directly influencing the detoxification capacity.

Our current study reveals that mutations in the vacuolar iron transporter genes *PstCCC1.2* and *PstCCC1.3* confer resistance to the DMI fungicide triadimefon in *Pst* through a Cyp51-independent mechanism centered on iron detoxification. We propose that inhibition of the heme-containing Cyp51 enzyme not only disrupts ergosterol biosynthesis but also triggers cytoplasmic iron dyshomeostasis, potentially via impaired function of membrane-associated proteins (e.g., V-ATPase) or the release of iron from perturbed heme pools. The ensuing accumulation of ferrous iron (Fe²⁺) amplifies oxidative stress through the Fenton reaction, triggering a lethal cascade of reactive oxygen species and lipid peroxidation that ultimately leads to cell death^39^. Resistance arises because the mutant PstCCC1.2 and PstCCC1.3 proteins exhibit enhanced iron-binding affinity and transport capacity, as also reflected in the biophysical assays, which are consistent with the functional data. By more efficiently sequestering cytotoxic Fe²⁺ into vacuoles, a detoxification process conserved in fungi, these mutants lower the cytoplasmic iron pool, thereby preventing iron-dependent lethal oxidative damage and enabling fungal survival under triadimefon stress.

This iron-mediated resistance represents a conserved adaptive strategy in fungi, evidenced by analogous mutations in the *Fg FgCCC1* genes conferring similar tolerance. While our findings underscore a critical link between iron homeostasis and antifungal efficacy in plant pathogenic fungi, they appear to contrast with a recent report indicating that iron availability does not alter DMI resistance in the human pathogen *C. albicans*^36^. This distinction may reflect fundamental differences in iron storage or regulatory networks in the fungal kingdom, or it may highlight that the gain-of-function mutations identified here unlock a resistance route not typically prominent under physiological iron manipulations. Consequently, targeting fungal iron homeostasis, for instance, by co-applying DMIs with iron chelators, can be a promising, pathogen-tailored strategy for managing fungal diseases of the crops and overcoming resistance to DMI fungicides.

The identification of *PstCCC1* mutations as a cause of low-level triadimefon resistance, independent of the moderate resistance conferred by Cyp51 Y134F, highlights two priorities for future research. First, field monitoring should assess whether isolates carrying both mutations emerge. Since the two mechanisms are independent, their combination could theoretically lead to additive or synergistic resistance, potentially resulting in the high-level resistance not yet observed. Proactive genetic crosses and population surveillance are needed to evaluate this risk. Second, although this study focused on Chinese *Pst* populations, the uncovered iron‑detoxification mechanism is evolutionarily conserved and likely relevant globally. The widespread occurrence of Cyp51 Y134F in *Pst* worldwide indicates similar selection pressures elsewhere. Screening global *Pst* genomes for *PstCCC1* polymorphisms should therefore be a priority to determine whether this resistance pathway is evolving beyond China. Tracking such mutational combinations and expanding geographic surveillance will be crucial for developing durable strategies to manage triazole resistance in wheat stripe rust and other fungal pathogens.

**Fig. 8:**
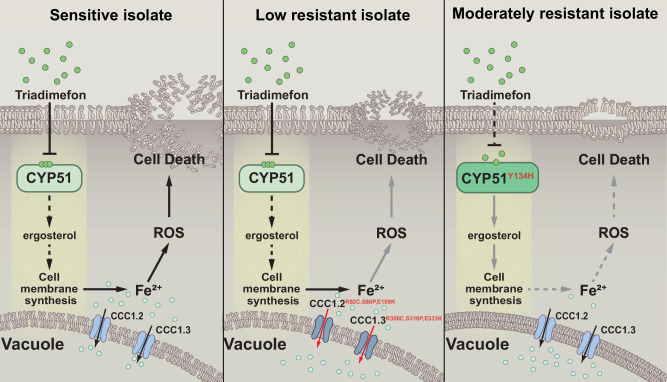
The model of the CCC1 proteins regulating triadimefon resistance in *Pst*. During the treatment of *Pst* with triadimefon, the decrease in ergosterol content disrupts iron homeostasis. The resulting iron overload promotes toxic reactive oxygen species (ROS) production via the Fenton reaction, leading to iron-dependent lipid peroxidation and cell death. Compared to the sensitive isolates, moderately resistant isolates carrying the Y134 mutation in the target gene *Cyp51*, which causes a change in protein conformation and a decrease in the binding affinity between triadimefon and the target protein, leading to a normal synthesis of ergosterol. This reduces damage to the pathogen cell membrane, and the level of iron-induced oxidative damage caused by ferrous ion overload inside the cell is greatly reduced compared to the sensitive isolates, resulting in a higher level of triadimefon resistance. Compared to the moderately resistant isolates, the low resistant isolates do not have amino acid mutations in the target gene *Cyp51*. Triazole fungicides like triadimefon can break through the first line of defense and cause large amounts of ferrous ions to accumulate in the cytoplasm. At this point, CCC1, which is responsible for transporting cytoplasmic iron ions to vacuoles to maintain iron homeostasis and detoxify, begins to function. When the iron transport functions of the resistance proteins PstCCC1.2^R82C,S86P,E109K^ and PstCCC1.3^R306C,S310P,E333K^ are stronger than that of the non-mutated genes, more cytoplasmic iron can be transported to vacuoles to more efficiently maintain iron homeostasis and detoxify to ensure the normal growth of the pathogen, thereby causing their resistance to triadimefon.

In summary, through genetic, bulked DNA sequencing, transcriptomic, protein affinity, and transformation analyses, we demonstrated that the *Pst* sensitivity to DMI fungicide triadimefon is positively regulated by *PstCCC1* genes. We propose a model wherein triadimefon inhibition of the heme-containing Cyp51 enzyme perturbs cellular iron homeostasis, leading to cytotoxic accumulation of Fe²⁺ in the cytoplasm. The excessive Fe²⁺ drives the generation of reactive oxygen species via the Fenton reaction, culminating in lipid peroxidation and cell death. The CCC1 mutant proteins counteract this by more effectively sequestering Fe²⁺, thereby suppressing the iron-dependent oxidative cell death cascade and enabling survival under fungicide stress (Fig. 8). Collectively, our results demonstrated that *Pst* regulates triadimefon resistance through vacuolar iron transporter-mediated iron homeostasis, providing a theoretical foundation for developing resistance management strategies to retard resistant population evolution and for discovering target-based fungicides against *Pst* and other fungal pathogens. We further suggest that combination of iron homeostasis disruptors with iron-chelating agents or alternate use of the different chemicals may serve as effective strategies to enhance control efficacy of fungicides. The development of inhibitor-targeting iron homeostasis maintenance proteins (particularly PstCCC1.3) still requires further studies, including the synthesis or screening of novel bioactive compounds, which can be either natural or synthetic.

## Methods

### Fungal isolates, wheat cultivars and fungicides

Isolates YQ324 and Qui1-2 of *Puccinia striiformis* f. sp. *tritici* (*Pst*) were the parental isolates used to develop a segregating population as described below. YQ324 and Gui1-2 were collected in 2017 from Qinghai and Guizhou provinces, China, respectively. YQ324 is a triadimefon low resistant isolate but does not carry any mutation in the DMI fungicide target gene *Cyp51*. Gui1-2 was the most triadimefon-sensitive isolate collected that year (unpublished data). To determine the presence of the resistance candidate gene mutants identified in the present study in the natural *Pst* population, 38 *Pst* isolates were selected from 486 isolates collected throughout China and used in a previous study^8^, including all 33 resistant isolates and 5 sensitive isolates. In addition, *Fg* strain PH-1 obtained from Professor Cong Jiang at Northwest A & F University was used for overexpression of resistance candidate genes and yeast (*S. cerevisiae*) strains BY4741 and ΔScCCC1(P47818) obtained from the Biovector Science Laboratory (Beijing, China) were used to validate the physiological and biochemical functions of the resistance candidate genes.

Wheat cultivars Mingxian169 (MX169), Suwon 11 (Su11) and Fielder were used in different experiments. *Pst* urediniospores were maintained and propagated on seedlings of MX169. Suwon11 was used for HIGS assays according to the previous study^40^. Fielder was selected for generating *PstCCC1*-RNAi transgenic plants due to its demonstrated high transformation efficiency^41,42^. Wheat seedlings were grown before inoculation, inoculated with *Pst* urediniospores, and grown after inoculation under the same conditions and using the same procedures as previously described^43^.

The following five fungicides were used in this study. 97.5% triadimefon, 97.5% tebuconazole, 97% pyraclostrobin and 97.5% hexaconazole were purchased from Xi’an Hytech Agrochemicals Co., Ltd. (Xi’an, Shaanxi, China). 97% flubeneteram was provided by Prof. Guangfu Yang’s team at Central China Normal University.

### Developing a *Pst* population segregating for triadimefon resistance

To determine the mechanisms of resistance to triadimefon in *Pst*, a uredinial population segregating for resistance was developed through crossing a resistant isolate (YQ324) with a sensitive isolate (Gui1-2) on *B. aggregata* plants as previously described^15^. Briefly, wheat leaves bearing telia were harvested from plants infected with the isolates in the greenhouse and soaked in sterile water for 24 h. After rinsing with sterile water, the leaves were cut into square pieces of about 1 mm in length using a blade and placed into plates containing 1% water agar. The plates were kept at 10 °C for 6–8 hours to allow teliospores to germinate and produce basidiospores. After dewaxing *B. aggregata* leaves with gloved figures, the leaves of barberry seedlings surrounded with a plastic cylinder were inoculated with basidiospores by hanging over an inverted plate containing germinating teliospores with the lid removed and kept in a dew chamber at 10 °C for 24 hours, and then move to a growth chamber at 16 °C and 16 hours of daily light for *Pst* to produce pycnia. When pycniospores appearing like honeydew on barberry leaves about 7 days after inoculation, pycniospores produced from isolate YQ324 were transferred onto pycnia produced by isolate Gui1-2 using sterile toothpicks, and the hybridized pycnia were marked in circles with a marker pen. After 8 to 10 days of hybridization, aecia containing aeciospores formed on the back side of leaves bearing the hybridized pycinia were cut from the barberry leaves, and aeciospores were spread onto a slide. Single aeciospores were picked using a fine glass needle under a microscope and transferred onto wheat leaves (cv. MX169), with one aeciospore on one leaf. When the wheat leaves started showing chlorotic patches about 5–7 days after inoculation, plants and leaves were cut off to keep only a single plant having a single inoculated leaf in each pot, and each pot was covered with a plastic cylinder to prevent cross contamination. The plants were kept under the same conditions to produce urediniospores. As *Pst* is dikaryotic and the F_1_ generation may consist of different genotypes, urediniospores from each wheat leaf were collected and kept separately. After testing the F_1_ isolates for triadimefon sensitivity, a single F_1_ isolate (Za 6-7) showing the highest level of triadimefon resistance (EC_50_ = 3.18 μg/mL) was selected to produce a F_2_ population on *B. aggregata* plants, and MX169 plants were inoculated with aeciospores, using the same procedures and conditions as described above, except by selfing.

### Fungicide sensitivity assay and EC_50_ determination

The sensitivity to fungicides of F_1_ and F_2_ isolates and isolates growing on HIGS-silenced plants or RNAi transgenic wheat lines was determined using an improved detached leaf method as previously described ^44^. Briefly, the first leaves of 10-day-old wheat cultivar Suwon 11 (for HIGS) and Fielder (for RNAi lines) seedlings were inoculated with a urediniospore suspension (2.5 mL, ~3 × 10⁵ spores/mL in Novec 7100). The suspension was pipetted onto a 5-cm marked segment of the first leaf. Detached leaf segments (5 cm) were excised six days post-inoculation and placed in Pedri dishes containing 0.5% water agar supplemented with 75 μg/mL 6-benzylaminopurine (6-BA) and varying concentrations of triadimefon, tebuconazole, hexaconazole, or flubeneteram (0.03–7.29 μg/mL). Petri dishes without triadimefon were used as control. Five leaf segments per dish were fixed at the ends with rigid plastic film and incubated in a growth chamber (16 °C/16 hours light; 12 °C/8 hours dark). After seven days, the leaf segments were photographed using the Canon EOS 90D (Canon Inc., Tokyo, Japan) and the sporulation area were quantified using software Photoshop (Adobe, San Jose, CA, USA). The inhibition percentage of sporulation relative to the control was calculated. The EC_50_ values, representing the fungicide concentration that caused a 50% reduction in sporulation area, were calculated by probit analysis using the Data Processing System (DPS) software version 7.05.

The sensitivity of the *Fg* heterologously expressing isolates to fungicides were assessed using the inhibition of mycelial growth method. Technical-grade fungicides were dissolved in methanol and added to the autoclaved, molten Potato Dextrose Agar (PDA) medium. The final concentrations were 0, 0.5, 1.0, 2.5, 5.0 and 10.0 µg/mL for each of the fungicides (triadimefon, tebuconazole, hexaconazole and flubeneteram). A mycelial plug taken from the actively growing margin of a 3-day-old colony was placed upside down in the center of each PDA plate. The inoculated plates were incubated at 25 °C in darkness for 3 days. The colony diameter was measured in two perpendicular directions, and the average diameter was calculated after subtracting the diameter of the original inoculum plug. The percentage of mycelial growth inhibition for each concentration was calculated relative to the non-fungicide control. The experiment was conducted with three replicates per concentration and repeated three times independently. The EC_50_ values were determined by probit analysis using software DPS v7.05^45^.

### DNA extraction and BSA-seq analysis

Genomic DNA was isolated from the urediniospores using the CTAB method^46^. After evaluating the F_2_ isolates (*n* = 83) for triadimefon sensitivity, the resistant and sensitive DNA bulks were made by pooling equal amount DNA from 20 isolates in each group with the highest and lowest EC_50_ values, respectively (Fig. 1a). The two bulks and the two parental isolates of DNA samples were sequenced by Novogene (Beijing, China). The quality of the raw sequencing data was evaluated and filtered using software Illumina Casava 1.8 version 1.2.3 (https://support-docs.illumina.com/SW/ClarityLIMS/ClarityINT/Content/SW/ClarityLIMS/Integrations/CASAVA182SampleSheet.htm). After completing the quality assessment of sequencing data, the clean sequencing reads were mapped using the *Pst* AZ2 genome as the reference (10.6084/m9.figshare.24265198.v5)^47^ and software BWA (parameter: mem-t4-k32-M), and the alignment results were deduplicated using SAMTOOLS (parameter: rmdup)^48^. The raw SNP calls were filtered using the GATK’s VariantFiltration module with stringent thresholds: QD < 4.0, FS > 60.0, MQ < 40.0 and genotype quality (GQ) < 20. ΔSNP-index and ED values were calculated using 200,000-bp windows and 20,000-bp step sizes, retaining windows with SNP counts ≥ 20. Quantitative trait locus (QTL) analysis was conducted with the SNP and the EC_50_ data of the 20 isolates in each of the sensitive and resistant bulks referring to the two parental isolates using the easyQTLseq package (https://github.com/laowang1992/easyQTLseq/releases/tag/v1.0.0). Chromosomes were integrated to visualize QTL regions^49^.

### Cloning triadimefon sensitivity genes and sequence analysis

Based on the QTL analysis and gene annotation, three candidate genes for triadimefon sensitivity, *PstCCC1.1*, *PstCCC1.2* and *PstCCC1.3*, were identified. To clone these genes, genomic DNA was isolated from urediniospores of the isolates using the CTAB method^46^, and total RNA was extracted from *Pst*-infected wheat leaves samples 72 hours after inoculation using the Quick RNA isolation Kit (Huayueyang Biotechnology, China, Beijing, 0416-50GK). These genes were amplified from the *ASM3951922v1* (https://www.ncbi.nlm.nih.gov/datasets/genome/GCA_039519225.1/) genomic cDNA samples (reverse-transcribed from RNA extracted from *Pst*-infected wheat leaves) (accession: *PstAZ2B06G00574*, *PstAZ2B06G00575* and *PstAZ2B06G00587*) using primers PstCCC1.1-F/R, PstCCC1.2-F/R and PstCCC1.3-F/R (Supplementary Data 6). The amplified products were cloned into the plasmid pMD19-T [TaKaRa Biotechnology (Dalian) Co., Ltd, Dalian, China], transformed into bacterial cell DH5α (Shanghai Weidi Biotechnology Co., Ltd, Shanghai, China) and sequenced by Sangon [Sangon Biotech (Shanghai) Co., Ltd, Shanghai, China]. The coding sequencies of the genes and their protein domains were analyzed using the HMMER software (http://www.ebi.ac.uk/Tools/hmmer/). The amino acid sequences of proteins of the candidate genes from other fungal species were retrieved from the NCBI database, and software Mega 7.0 (https://www.megasoftware.net/) was used to construct a phylogenetic tree for comparison of *Pst* with different fungal species based on these genes using the neighbor-joining method.

### HIGS assay of *Pst* candidate genes through BSMV-mediated gene silencing

To determine the role of the three candidate genes for triadimefon resistance, one specific cDNA fragment of these genes was selected for BSMV-HIGS using the previously described method^25^. HIGS is a technique that utilizes viral vectors (BSMV) to deliver RNAi constructs into host plants, leading to the silencing of target genes in the *Pst*. These fragments were not similar to any genes of the wheat genome databases determined by Basic Local Alignment Search Tool (BLAST) searching (https://urgi.versailles.inra.fr/blast/). The recombinant BSMV-HIGS vectors were constructed with these genes for use in the gene silencing experiment using the method described by Liu et al.^50^ For HIGS assay, the specific cDNA segments of *PstCCC1.1*, *PstCCC1.2* and *PstCCC1.3*, which were predicted using the siRNA finder software Si-Fi^51^, were inserted into the BSMV-γ carriers with *Not*I and *Pac*I restriction sites (Thermo Fisher Scientific, Waltham, Massachusetts, USA). The wheat plants inoculated with BSMV:*TaPDS* (phytoene desaturase) were used as the positive control, whereas the BSMV-γ-inoculated plants served as the negative control. The second leaves of Su11 were inoculated with BSMV and incubated in a plant growth chamber at 25–27 °C. When the plants inoculated with BSMV:*TaPDS* exhibited the expected photobleaching phenotype, 9–12 days after inoculation, the fourth leaves were further inoculated with low-resistant isolate YQ324. To evaluate the silencing efficiency, leaf samples were collected at 24, 48 and 72 hours post-inoculation (hpi) with *Pst*, prior to any fungicide treatment. The fungal biomass was assessed 10 days after triadimefon treatment^52^. This time point was selected as it corresponds to the peak of sporulation on untreated control plants, allowing for a robust comparison of the fungicide’s inhibitory effect. Furthermore, it precedes the natural dispersal of urediniospores, ensuring accurate and consistent quantification. This protocol is consistent with our established methods for evaluating fungicide efficacy against *Pst*^52^.

### Histological observations of fungal growth

To characterize the functions of *PstCCC1.1*, *PstCCC1.2* and *PstCCC1.3* in *Pst* responses to triadimefon, *Pst* development was observed by microscopy. Wheat seedlings (cv. MX169) were inoculated with *Pst* (isolate YQ324, low resistant to triadimefon) and sprayed evenly with an acetone suspension of triadimefon (97.5% powder) at the rate of 10 μg/mL on wheat leaves 3 days post *Pst* inoculation (dpi). Acetone was sprayed on wheat leaves as a control. Wheat leaf segments (2 cm) were collected at 72 hours post triadimefon treatment (hpt). The methods for leaf sample preparation and wheat germ agglutinin (WGA) staining were conducted as previously described^53^. *Pst* hyphae were stained with WGA conjugated to Alexa-488 (Invitrogen, Carlsbad, CA, USA). WGA is a lectin that binds specifically to N-acetylglucosamine residues, a major component of chitin in the fungal cell wall, enabling clear fluorescence-based detection of the pathogen. Stained samples were observed using an Olympus BX53F fluorescence microscope (Olympus, Tokyo, Japan) under blue-light excitation (excitation wavelength 450–480 nm, emission wavelength 515 nm). To measure the leaf areas containing *Pst* hyphae, 50 random infection sites were examined for each treatment.

### HIGS of Cyp51 in *Pst* and comparative response profiling

To compare the dependence on *Cyp51* for triadimefon resistance among isolates of differing sensitivity. A 253-bp *Cyp51*-specific cDNA fragment, which was verified for the lack of homology to the wheat genome by BLAST analysis against the URGI wheat database (https://urgi.versailles.inra.fr/blast/), was cloned into the BSMV-γ vector using *Not*I and *Pac*I restriction sites (Thermo Fisher Scientific, Waltham, Massachusetts, USA). Wheat seedlings (cv. MX169) at the two-leaf stage were rub-inoculated with BSMV:γ-C*yp51*, and plants inoculated with BSMV:γ-*TaPDS* and BSMV:γ were used as positive and negative controls, respectively. Inoculated plants were maintained at a diurnal cycle of 25 °C to 27 °C corresponding to the 8 hours dark and 16 hours light periods. At 9–12 days post-viral inoculation when TaPDS-silenced plants showed photobleaching, the fourth leaves were inoculated with *Pst* isolates Gui 1-2 (triadimefon sensitive), YQ324 (low-resistant) or TS-7 (moderately resistant). Silencing efficiency was determined by reverse transcription quantitative polymerase chain reaction (RT-qPCR) using primers listed in Supplementary Data 6 (qRT-Cyp51-F/R). For phenotypic and biochemical characterization. Fungal biomass was quantified in *Cyp51*-silenced and non-silenced plants under triadimefon-treated and untreated conditions. Ergosterol content and cytosolic iron levels were subsequently measured in the same set of samples across the four combinatorial treatment groups (non-silenced/silenced × untreated/treated) using high-performance liquid chromatography (HPLC) and inductively coupled plasma mass spectrometry (ICP-MS), respectively. For detailed procedures on ergosterol content and cytosolic iron measurement, see the sections “Analysis of ergosterol content in *Pst*” and “Analysis of Fe content in *Pst*, yeast, and the Fusarium head blight fungus” below.

### Generation and characterization of wheat plants transformed with *Pst* resistance candidate genes

The wheat cultivar Fielder was used to generate transgenic plants. To obtain RNAi-silenced transgenic wheat plants, the specific cDNA segments of *PstCCC1.1*, *PstCCC1.2* and *PstCCC1.3* from sensitive *Pst* isolate Gui1-2 were inserted into the PC336 (Ubi: GWRNAi:Nos) plasmids (Thermo Fisher Scientific, Waltham, Massachusetts, USA) using the gateway cloning method^54^. All recombinant constructs were transformed into *Agrobacterium* strain EHA105 using an *Agrobacterium*-mediated transformation system^55^. For transformation, wheat calli were co-cultivated with *Agrobacterium* (OD_600_ = 0.6, 22 °C, 3 days, dark) and selected on hygromycin media. Positive T_0_ transformants were self-pollinated to generate T_1_ and subsequent generations. Homozygous T_3_ lines were then spray-inoculated with *Pst* YQ324 urediniospore suspension (2 mg/mL) and grown at 16 °C (16-h light, 70% RH). To confirm and quantify the silencing efficiency prior to fungicide exposure, leaf samples were collected from a subset of inoculated plants at 48 hours post-inoculation for RNA extraction and RT-qPCR analysis. For phenotyping, transgenic and control plants were spray-inoculated with the low-resistant isolate YQ324. Fungal biomass was quantified by qPCR in plants treated with triadimefon (Tri), Tri + FeSO₄ or Tri + FeSO₄ + BPS. Triadimefon sensitivity was further assessed by calculating EC₅₀ values for triadimefon, tebuconazole, hexaconazole and flubeneteram. Cytosolic and total iron contents were determined using ICP-MS, and the expression of iron acquisition genes was analyzed through RT-qPCR. Silencing specificity was confirmed by measuring transcript levels of all three *PstCCC1* paralogs in each RNAi line.

### Assessment of transcription levels of *Pst* resistance genes in wheat plants after inoculation and triadimefon treatment using real-time PCR

To assess the expression levels of *PstCCC1.1*, *PstCCC1.2* and *PstCCC1.3*, wheat cv. MX169, which is susceptible to *Pst* isolate YQ324, was grown in controlled chambers (18 °C, 70% RH, 16-h light). At the two-leaf stage, 120 plants were inoculated with YQ324 (2 mg urediniospores/mL) and incubated for 24 hours (100% humidity, dark). Plants were then transferred to 16 °C with 14-h light. At 72 hpi, triadimefon (10 μg/mL) or control solution was applied. Leaf samples from both groups were collected at 6–120 hpt for analyzing *PstCCC1.1-1.3* expression. qRT-PCR was performed in a 25 μL reaction mixture containing 12.5 μL of Light Cycler SYBR Green I Master Mix (GenScript Biotech Corporation, Nanjing, China), 2 μL of diluted cDNA (1:5), 8.9 μL of distilled H_2_O, 0.8 μL of forward primer (10 mM) and 0.8 μL of reverse primer (10 mM) on a CFX Connect Real-Time System (Bio-Rad, Hercules, CA, USA). The primers are listed in Supplementary Data 6. The real-time PCR data were analyzed using the 2^-ΔΔCT^ comparative method to estimate relative gene expression. The expression levels of all tested genes were normalized using the levels of house-keeping genes *PstEF* and *TaEF*. Three biological replications were used for each of the time points, and the experiment was conducted three times.

### Physiological and biochemical characterization of candidate resistance genes and their mutants

To physiologically and biochemically characterize the candidate genes (*PstCCC1.1*, *PstCCC1.2* and *PstCCC1.3*) and their mutants, yeast vectors were constructed using a previously described method^56^. Briefly, the cDNA of each gene was amplified from isolates Gui1-2 and YQ324 using primers listed in Supplementary Data 6 (pDR195-PstCCC1.1/PstCCC1.1^Y300H^-F/R, pDR195-PstCCC1.2/PstCCC1.2^R82C,S86P,E109K^-F/R and pDR195-PstCCC1.3/PstCCC1.3^R306C,S310H,E333K^-F/R) and inserted into the *Not*I/*BamH*I restriction sites and fused in-frame to the C-terminus of the GFP reporter gene in plasmid pDR195 (Thermo Fisher Scientific, Waltham, Massachusetts, USA) to obtain the complementation constructs pDR195-*PstCCC1.1/*PstCCC1.1^Y300H^, pDR195-*PstCCC1.2/*PstCCC1.2^R82C,S86P,E109K^ and pDR195-*PstCCC1.3/*PstCCC1.3^R306C,S310H,E333K^. All constructs were under the control of the constitutive LacZ_a promoter in the pDR195 vector. The yeast vectors were transformed into the ΔScCCC1 yeast (*Sc*) strain (Biovector Science Lab, Beijing, China) via the lithium acetate method. The SD-U liquid medium was used to culture the transformed yeast cells and grown to OD_600_ = 0.1. To clarify the experimental design, we prepared four 10-fold dilution series of yeast cells under sterile conditions and monitored their growth by recording the A_600_ values every 3 h. The measurements began after 18 hours of cultivation under the following four treatments: (1) no addition, (2) 20 mM FeSO₄, (3) 10 μg/mL triadimefon and (4) 20 mM FeSO₄ plus 10 μg/mL triadimefon, in order to generate the growth curves.

### Overexpression of Pst candidate genes for tridimefon resistance in *Fg*

To express *PstCCC1.1*, *PstCCC1.2* and *PstCCC1.3* and their variants in *Fg*, *Fg* isolate ΔFgCCC1 was used to produce overexpression isolates. The *FGSG_07832* (*FgCCC1)* gene knockout mutant (ΔFgCCC1) was generated in the wild-type strain PH-1 using a split-marker homologous recombination strategy^57^. Briefly, approximately 1.0 kb of the upstream and downstream flanking sequences of the *FgCCC1* open reading frame were amplified by PCR using primer pairs (listed in Supplementary Data 6) designed with overlaps to the hygromycin B phosphotransferase gene (*hph*). The *hph* cassette, which confers resistance to hygromycin B, was used as a selectable marker. The upstream-*hph* and *hph*-downstream fragments were fused by double-joint PCR to form the final deletion cassette. This cassette was then transformed into PH-1 protoplasts via polyethylene glycol (PEG)-mediated transformation. Putative transformants were selected on TB3 agar plates containing 100 μg/mL hygromycin B. Successful gene replacement events were initially screened by diagnostic PCR using gene-specific and *hph*-specific primers (Supplementary Data 6).

For transfection assays, constructs of the genes and their variants were made using the method described in Xu et al.^58^. Briefly, the cDNA of each gene amplified from Gui1-2 and YQ324 using primers listed in Supplementary Data 6 (pFL2-PstCCC1.1/PstCCC1.1^Y300H^-F/R, pFL2-PstCCC1.2/PstCCC1.2^R82C,S86P,E109K^-F/R and pFL2-PstCCC1.3/PstCCC1.3^R306C,S310H,E333K^-F/R) and cloned into the *Xho*I-digested pFL2 vector carrying the Geneticin resistance marker and a GFP tag (Thermo Fisher Scientific, Waltham, Massachusetts, USA). The pFL2 vector contains the constitutive RP27 promoter for strong expression, with the inserted gene fused in-frame to the C-terminus of GFP, and a geneticin (G418) resistance marker for selection. The constructs were transformed into protoplasts of the ΔFgCCC1. Protoplast preparation and PEG (polyethylene glycol)-mediated transformation of *Fg* were performed as described previously^59^.

To validate the conservation of the resistance mechanism, a site-directed mutant of FgCCC1 was generated. The amino acid residues R181 and S185 in FgCCC1, which are homologous to R306 and S310 in PstCCC1.3, were selected for mutagenesis. The DNA sequence encoding the FgCCC1 open reading frame, with codons mutated to introduce the R181P and S185D substitutions, was designed in silico and chemically synthesized in vitro by Sangon Biotech (Shanghai, China). The synthesized mutant gene, *FgCCC1*^*R181P,S185D*^, was delivered in a standard cloning vector. The gene fragment was then subcloned into the *Xho*I -digested pFL2 vector. The resultant plasmid was transformed into protoplasts of the ΔFgCCC1 knockout mutant using PEG-mediated transformation, as described above. All complementation strains contained transgene integrated ectopically in multiple copies. To verify successful transfection of these genes, all transformants were analyzed through RT-PCR using the specific primers listed in Supplementary Data 6. Positive transfected isolates were obtained and used for further analysis.

### Analysis of Fe content in *Pst*, yeast and the Fusarium head blight fungus

To quantify cytosolic and total iron content across different fungi, tailored methods were employed. For the obligate biotroph *Pst*, *Pst* hyphae were isolated from infected wheat leaves inoculated within a defined 5-cm segment. After fungicide and other treatment, leaf tissues were harvested and digested with an enzyme solution containing 1.5% (w/v) cellulase (Sigma-Aldrich, St. Louis, MO, USA) and 0.5% (w/v) pectinase in 0.6 M mannitol (Sigma-Aldrich, St. Louis, MO, USA) for 4 hours at 28 °C to selectively degrade plant cell walls and release fungal structures. The fungal-enriched fraction was collected from the filtrate by centrifugation at 4,000× g for 10 min at 4 °C. The resulting pellet was resuspended and split into two aliquots for parallel processing. For total iron analysis, one aliquot was washed, transferred to a pre-weighed tube, and lyophilized to determine the fungal dry weight. The lyophilized biomass was then digested in 65% trace metal-grade HNO₃ at 95 °C for 4 hours for subsequent iron measurement. For cytosolic iron analysis, the second aliquot was processed for subcellular fractionation. The cells were converted to spheroplasts using zymolyase (Sigma-Aldrich, St. Louis, MO, USA). The spheroplasts were lysed in a hypotonic buffer (10 mM Tris-HCl, pH 7.4, 1 mM EDTA), and the lysate was subjected to differential centrifugation: first at 1000 × *g* for 10 min at 4 °C to remove unbroken cells and nuclei, then at 10,000 × *g* for 15 min at 4 °C to pellet heavy organelles and vacuoles. The resulting supernatant was further clarified by ultracentrifugation at 100,000 × *g* for 1 hours at 4 °C to obtain the final cytosolic fraction (supernatant), which was then processed for acid digestion. The iron content in both the total biomass digests and the cytosolic fraction digests was determined using ICP-MS. Non-inoculated healthy leaves processed identically served as the background control. All iron content values from infected samples were corrected by subtracting the background iron levels from the healthy controls. The final data for both total and cytosolic iron were normalized to the dry weight of the fungal biomass, expressed as microgram of iron per gram of dry weight (μg/g).

The cytosolic and vacuolar iron content in *Sc* was measured as previously described^57^. In brief, cells were harvested by centrifugation (3000 × *g*, 5 min), washed twice with ice-cold phosphate-buffered saline (PBS) containing 1 mM EDTA to remove surface-bound iron, and resuspended in 1 mL PBS. For subcellular fractionation, spheroplasts were generated using zymolyase (Sigma, St Louis, MO, USA). Cytosolic and vacuolar fractions were isolated via differential centrifugation^57^. Briefly, spheroplasts were lysed in hypotonic buffer (10 mM Tris-HCl, pH 7.4, 1 mM EDTA) and centrifuged (10,000 × *g*, 15 min) to pellet vacuoles. The supernatant (cytosolic fraction) was further clarified (100,000 × *g*, 1 h). Vacuoles were purified using a Ficoll density gradient method^60^. Samples were digested in 65% HNO₃ at 95 °C for 4 h, followed by dilution in ultrapure water. Fe²⁺ concentrations were determined using Agilent 7900 ICP-MS (Agilent, Santa Clara, CA, USA) with ⁵⁶Fe as the monitored isotope^61^.

The cytosolic and total iron content in *Fg* were measured using the method as previously described^57^ with modifications for the ICP-MS analysis. Briefly, mycelia of the tested cultures were harvested after cultivation in complete medium (CM) for 36 hours at 25 °C, with or without prior fungicide or iron stress treatments. The harvested mycelia (approximately 50 mg fresh weight) were digested into protoplasts by incubation in a solution containing 2% cellulase (Sigma, St Louis, MO, USA), 2% lysozyme (Sigma, St Louis, MO, USA) and 0.2% driselase (Sigma, St Louis, MO, USA) for 4–6 h. The resulting protoplast suspension was filtered through a funnel with filter paper to remove debris and then centrifuged at 5000 × *g* at 4 °C for 10 min to pellet the protoplasts. For total iron content measurement, the pelleted protoplasts were directly processed for acid digestion. For cytosolic fraction isolation, the protoplast pellet was carefully resuspended in ice-cold lysis buffer (20 mM HEPES-KOH, pH 7.5, 0.25 M sucrose, 1 mM EDTA, and protease inhibitors) to osmotically lyse the protoplasts. The lysate was then subjected to a series of differential centrifugation steps to isolate the cytosolic fraction. Specifically, the crude lysate was first centrifuged at a low speed of 600 × *g* for 10 min at 4 °C to pellet unbroken cells and nuclei. The resulting supernatant was subsequently transferred to a new tube and centrifuged at a high speed of 12,000 × *g* for 30 min at 4 °C to pellet the heavy organelles, including mitochondria, peroxisomes and lysosomes. The final supernatant from this high-speed spin, which represents the cytosolic fraction devoid of nuclei and organelles, was carefully collected and used for subsequent ICP-MS analysis.

### Analysis of ergosterol content in *Pst*

The method for harvesting mycelia of the tested *Pst* (including *Cyp51*-silenced/non-silenced, sensitive, low-resistant and moderate-resistant isolates treated with or without fungicide) was consistent with that described in the “Analysis of Fe content in *Pst*” section. Following harvest, the fresh mycelia were dried at 60 °C for 3 h. Total ergosterol was extracted from the dried mycelia using a previously published protocol^62^. Briefly, the dried mycelial samples were saponified and then ergosterol was extracted with n-hexane. The ergosterol concentrations in the extracts were quantified using a High-Performance Liquid Chromatography (HPLC) system (Prominence SPD-20A, Shimadzu, Japan). Separation was achieved at room temperature on a Hypersil BDS C18 analytical column (250 × 4.6 mm, 5 μm) using 100% methanol (chromatography grade) as the mobile phase. The detection wavelength was set at 282 nm. Ergosterol identification was based on the retention time and co-chromatography with a commercial ergosterol standard (Sigma-Aldrich, St. Louis, USA). Quantification was performed by comparing the peak areas of the samples to those of the standard curve.

### RNA-seq data analysis of *Pst* gene expression in wheat plants after inoculation and triadimefon treatment

A total of 300 wheat plants (cv. MX169) were grown under controlled greenhouse conditions (16 °C, 16-h light/8-h dark cycle) until reaching the two-leaf stage. Inoculation was performed by spraying urediniospore suspensions (2 mg/mL; *Pst* isolate YQ324) onto the plants. At 24 hpi, triadimefon (10 μg/mL) was applied, with sterile water serving as the control treatment. Leaf samples (100 mg per biological replicate; *n* = 3 per treatment) were collected at 0, 24, 48, 72 and 120 hpi following triadimefon application. All samples were immediately flash-frozen in liquid nitrogen and stored at −80 °C for subsequent RNA extraction. Total RNA was extracted using TRIzol reagent (Invitrogen) followed by DNase I treatment (Thermo Fisher Scientific, Waltham, MA, USA). RNA integrity was verified by Bioanalyzer (RIN ≥ 7.0, Agilent Technologies). Strand-specific cDNA libraries were prepared using the NEBNext Ultra II RNA Library Prep Kit (NEB) with poly(A) selection and fragmented to 300-bp inserts. Paired-end sequencing (150 bp) was performed on an Illumina HiSeq 4000 platform (Guangzhou GENE DENOVO Technologies). An index of the reference genome was built, and paired-end clean reads were mapped to the reference genome using HISAT2. 2.4 and other parameters set as a default^63^. The mapped reads of each sample were assembled using StringTie v1.3.1^64^ in a reference-based approach. For each transcription region, a FPKM (fragment per kilobase of transcript per million mapped reads) value was calculated to quantify its expression abundance and variations, using the RSEM software^65^. Principal component analysis (PCA) of the RNA sequences was performed with the R package (http://www.r-project.org/). Differentially expressed genes (DEGs) were analyzed using the R package DESEQR (v.3.7.1) and then used in the Kyoto Encyclopedia of Genes and Genomes (KEGG) and Gene Ontology (GO) enrichment analyses with the R package CLUSTERPROFILER (*P* < 0.05, FOLDCHANGE > 2.0).

### Assessment of cytosolic ROS and lipid peroxidation in yeast transformed with the *Pst* candidate genes for triadimefon resistance

Flow cytometry was used to detect lipid peroxidation and assess reactive oxygen species (ROS) levels in yeast heterologous transformants of the candidate genes and their mutants under iron stress conditions, following established methods^39^. Briefly, yeast cells were placed in 96-well plates treated with different reagents. After 10 h, yeast cells were incubated with 10 μmol DCFH-DA (Sigma, MO, USA) fluorescence probe diluted in PBS at 37 °C for 30 min. The ROS level in each sample was detected using a flow cytometer (Beckman, Brea, CA, USA) and analyzed using the FlowJo software (FlowJo LLC, Delaware, USA). To assess the lipid peroxidation levels in each sample after different treatments, 2 μmol C11-BODIPY581/591 (Thermo Fisher, Waltham, MA, USA) was loaded for 30 min. The lipid peroxidation level was quantified as the ratio of green (oxidized) to red (reduced) fluorescence intensity (FL1/FL2) using the flow cytometer.

### Expression and purification of PstCCC1 proteins for biophysical analyses

All three PstCCC1 proteins and their mutants were produced using an established *E. coli*-based continuous-exchange cell-free protein synthesis (CFPS) platform coupled with liposome-assisted refolding. This approach, in which SDS-solubilized precipitates are refolded upon rapid dilution into lipid bilayers, has been validated by structural and functional studies, including crystallographic evidence of correct folding for reconstituted membrane proteins ^66^. The broader applicability of cell-free systems for producing challenging transporters and channels has been extensively reviewed^67^. Prior to final construct selection, we performed in silico hydrophobicity and transmembrane domain predictions for each homolog and codon-optimized all genes for optimal expression. Each gene was then systematically tested with six different N-terminal fusion tags-His, GST, SUMO, MBP, and no tag-under various expression and refolding conditions. For PstCCC1.2 and PstCCC1.3, small tags (SUMO and His, respectively) were sufficient to obtain soluble, functional protein after cell-free synthesis and liposome refolding. For PstCCC1.1, however, none of the small tags yielded detectable soluble protein; only fusion to the large solubility-enhancing partner MBP enabled sufficient recovery, and this tag was therefore used for all subsequent experiments. Thus, *PstCCC1.1* and *PstCCC1.1*^*Y300H*^ were amplified from *Pst* isolates Gui1-2 and YQ324 using primers pMAL-PstCCC1.1/PstCCC1.1^Y300H^-F/R and cloned into the *EcoR*I/*Hind*III restriction sites in the pMAL-C2x vector (Thermo Fisher Scientific, Waltham, Massachusetts, USA) to obtain PstCCC1.1*-*MBP and PstCCC1.1^Y300H^*-*MBP. *PstCCC1.2* and *PstCCC1.2*^*R82C,S86P,E109K*^ were amplified from Gui1-2 and YQ324 using primers pSmart-PstCCC1.2/PstCCC1.2^R82C,S86P,E109K^-F/R (Supplementary Data 6) and cloned into the *BamH*I/*Xho*I restriction sites in the pSmart-I vector (Thermo Fisher Scientific, Waltham, Massachusetts, USA) to obtain PstCCC1.2*-*sumo and PstCCC1.2^R82C,S86P,E109K^*-*sumo. *PstCCC1.3* and *PstCCC1.3*^*R306C,S310P,E333K*^ were amplified from Gui1-2 and YQ324 using primers pET28a-PstCCC1.3/PstCCC1.3^R306C,S310H,E333K^-F/R (Supplementary Data 6) and cloned into the *EcoR*I/*Xho*I restriction sites in the pET28a vector (Thermo Fisher Scientific, Waltham, Massachusetts, USA) to obtain PstCCC1.3*-*His and PstCCC1.3^R306C,S310P,E333K^*-*His.

Cell-free protein synthesis reactions were carried out at 30 °C for 16 hours in the absence of any hydrophobic supplement, causing the target proteins to accumulate as non-denatured precipitates. The precipitates were collected by centrifugation, washed, and solubilized in buffer containing 1% SDS and 0.1 M DTT. Refolding was initiated by rapid ten-fold dilution of the SDS-solubilized proteins into pre-formed liposomes composed of *E. coli* polar lipids, followed by incubation at 25 °C for 4 hours to allow bilayer insertion and folding. Proteoliposomes were collected by ultracentrifugation, solubilized with 1% DDM, and the refolded proteins were purified by affinity chromatography using the respective tags. PstCCC1.1-MBP fusions were purified on amylose resin and eluted with 10 mM maltose. PstCCC1.2-SUMO fusions were purified on Ni-NTA resin under native conditions, followed by on-column SUMO protease cleavage to remove the tag. PstCCC1.3-His fusions were purified on Ni-NTA resin in the presence of 0.05% DDM and eluted with 300 mM imidazole. All purified proteins were then subjected to size-exclusion chromatography on a Superdex 200 Increase column equilibrated with assay buffer (20 mM HEPES pH 7.4, 100 mM NaCl, 0.05% DDM). Peak fractions corresponding to monodisperse protein were pooled, concentrated, and buffer-exchanged into fresh assay buffer using 10 kDa molecular weight cutoff centrifugal filters.

For quality control, an analytical aliquot of each purified, refolded protein was analyzed by SDS-PAGE. The band corresponding to the expected molecular weight was excised, destained, subjected to in-gel tryptic digestion, and analyzed by LC-MS/MS, which confirmed protein identity with over 95% sequence coverage. This excision step was performed solely for identity confirmation and was never used to recover material for downstream biophysical assays. All microscale thermophoresis and isothermal titration calorimetry measurements were conducted using the independently purified, liposome-refolded, and buffer-exchanged proteins described above.

### In vitro iron affinity assay of *Pst* proteins for triadimefon resistance

Microscale thermophoresis (MST) and isothermal thermal titration (ITC) analysis were used to determine the affinity of iron ions and the three candidate proteins, respectively, according to previous studies^68,69^. Briefly, different concentrations of proteins were labeled with fluorescent labeling dye (Monolith His-tag labeling kit RED-tris-NAT 2^nd^ generation, Nano Temper Technologies) at 100 nM for 30 min at room temperature. Then, 1 μM/mL of FeSO_4_ was diluted into 16 PCR tubes using a 10-fold dilution concentration gradient and then added with 10 μL of labeled protein to each PCR tube in sequence. Other experimental conditions were set to be consistent with the Monolith NT (Nano Temper Technologies GMBH), and data analyses were performed using the MO Affinity Analysis v. 2.3 software^70^. For ITC assay, 100 nM FeSO_4_ solution was loaded into a 200 μL syringe, and 500 nM protein solutions were injected into the reaction tank. The titration interval was set to 300 s/5 μL. Under continuous stirring, the FeSO_4_ solution was continuously dropped into the reaction tank 50 times. The control was included in the experiment to measure the dilution heat of protein and water and obtained the binding isotherm by correcting the dilution heat. The data were analyzed using the Nano Analyze software^71^.

### Molecular docking analysis of *Pst* proteins for triadimefon resistance

The three-dimensional structures of the CCC1 proteins from *Pst* and *Fg* were predicted using AlphaFold3.0 (https://alphafoldserver.com/). Blind docking between these PstCCC1 proteins and iron ions was performed with Vina to obtain docking scores and binding sites. Saturation mutagenesis of the Vina-predicted binding sites in FgCCC1 was conducted using FoldX to evaluate the effects of mutations on protein stability and binding affinity (https://foldxsuite.crg.eu/). Software PyMOL (https://pymol.org/) was used to visualize docking results and analyze the interaction patterns between PstCCC1 and iron ions.

### Statistical analyses

Quantitative data, such as relative expression levels of *Pst* candidate genes for triadimefon resistance, infection area, biomasses of urediniospores, mycelial growth, relative inhibition, Fe content, yeast growth, DCFH intensity and PE/FITC ratio were analyzed using software DPS ver. 7.05^72^. To plot the sensitivity to triadimefon, EC_50_ values were calculated using nonlinear regression analysis (four-parameter logistic model) and transformed to relative logarithm values. Spearman correlation analysis was conducted with the log-transformed EC_50_ values to assess replicate reproducibility in the fungicide sensitivity assay. All quantitative data are shown as mean ± standard deviation (SD). Different letters indicate significant differences as determined by one-way analysis of variance (ANOVA) with Duncan’s new multiple range test (*P* < 0.05).

### Reporting summary

Further information on research design is available in the Nature Portfolio Reporting Summary linked to this article.

## Supplementary information

Supplementary Information

Description of Additional Supplementary Files

Supplementary Data 1-7

Reporting Summary

Transparent Peer Review file

## Source data

Source data

## Data Availability

The BSA-seq and transcriptomic data generated in this study have been deposited in the China National GeneBank DataBase (CNGBdb) under accession codes CNP0007785 and CNP0007780. The Pst AZ2 reference genome used in this study is available in the NCBI database under accession code GCA_039519225.1 [https://www.ncbi.nlm.nih.gov/datasets/genome/GCA_039519225.1/]. The detailed sequence information for genes *PstCCC1.1* (*PstAZ2B06G00574*), *PstCCC1.2* (*PstAZ2B06G00575*) and *PstCCC1.3* (*PstAZ2B06G00587*) including identified allelic variations, are provided in the Supplementary Information file. Source data are provided with this paper.
