# Supplementary material for: Vacuolar iron transporters mediate resistance to triadimefon in plant pathogenic fungi
Source: Nat Commun. 2026 May 6;17:6379. doi: 10.1038/s41467-026-72157-6 (PMC13376413; doi:10.1038/s41467-026-72157-6)
Supplement: Supplementary file 1 — Supplementary Information [file 41467_2026_72157_MOESM1_ESM.pdf]

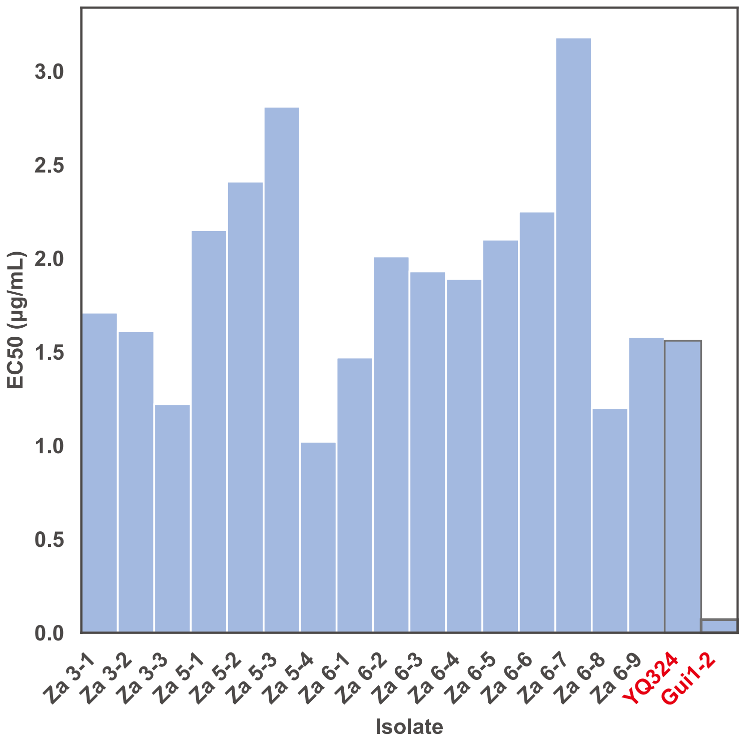

Supplementary Fig. 1. Triadimefon sensitivity of F_1 isolates of *Puccinia striiformis* f. sp. *tritici* (*Pst*) from a cross between the resistant isolate (YQ324) and the sensitive isolate (Gui1-2).

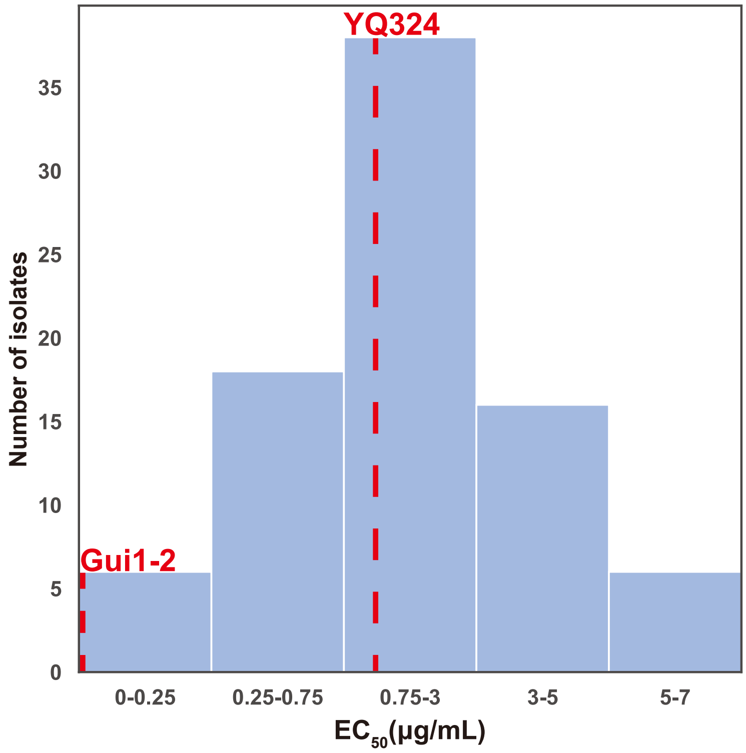

Supplementary Fig. 2. Distribution of triadimefon sensitivity in the F_2 population of *Pst* from a cross between the resistant isolate (YQ324) and the sensitive isolate (Gui1-2).

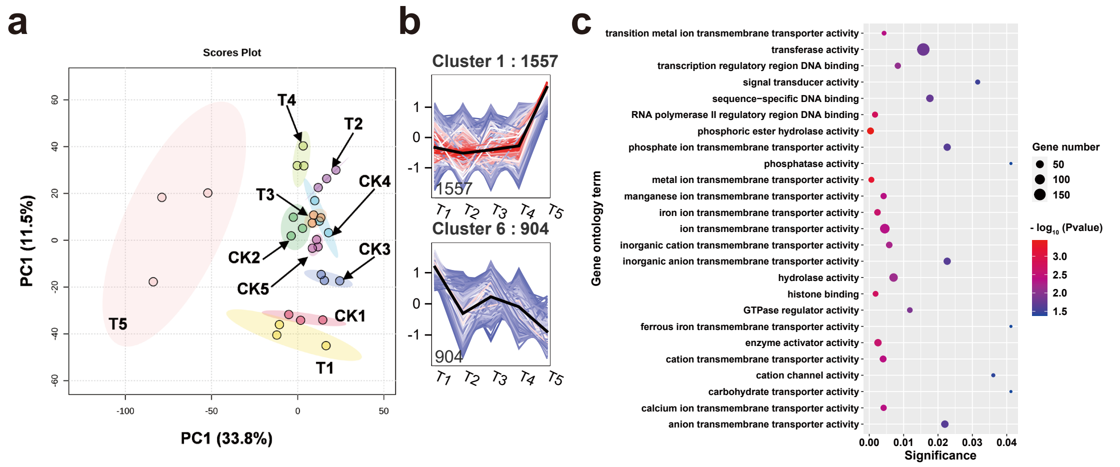

Supplementary Fig. 3. RNA-Seq analysis of *Pst* sensitivity to triadimefon. The *Pst* isolate used in this experiment was the resistant isolate (YQ324), and the wheat cultivar was MX169. **a** The RNA-seq analysis of principal component analysis (PCA) plots of transcriptome profiles of *Pst* at different temporal phases of triadimefon treatment. Ellipses show 95% confidence intervals. The samples with different treatments were shown in different colors. T1-T5 and CK1-CK5 denote samples collected at 0, 24, 48, 72, and 120 h post-treatment with triadimefon and the untreated control, respectively. **b** Expression trend analysis on transcriptome profiles of *Pst* at distinct temporal phases of triadimefon treatment. The trend analysis identified two expression modules (cluster1 and cluster6) of genes that continuously up and down-regulated in *Pst* during fungicide treatment process. **c** Scatter plot displays the gene ontology enrichment results based on genes from cluster1 and cluster6. The various color levels displayed different levels of significance of terms from low (blue) to high (red). The plot size represented the number of genes in each gene ontology term.

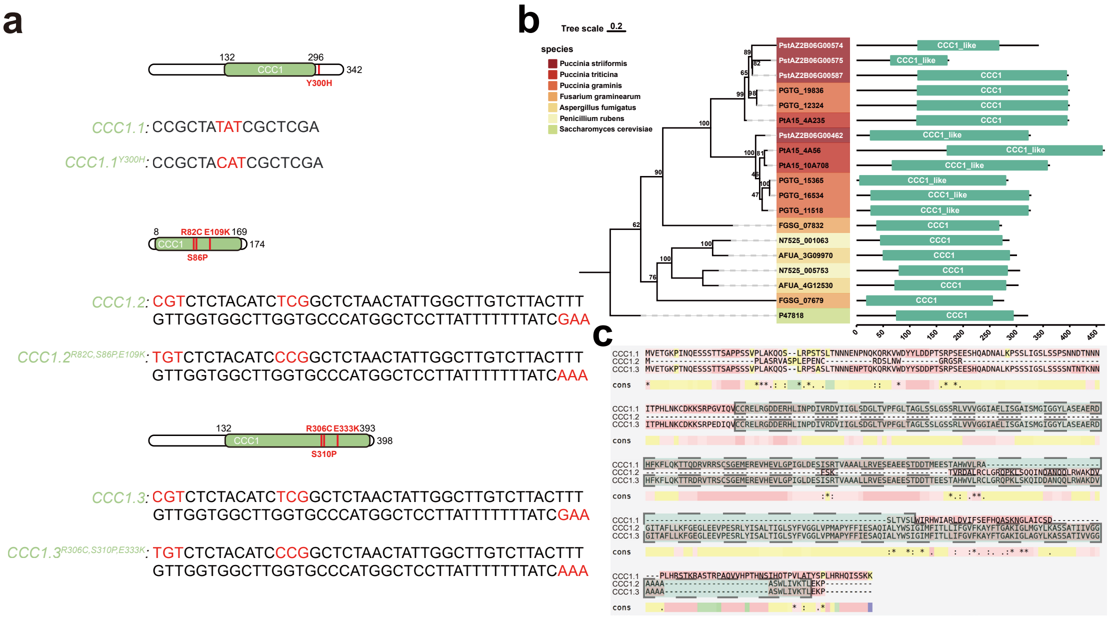

Supplementary Fig. 4. Sequence alignment and mutation site analysis of triadimefon sensitivity genes. **a** Identification of mutation sites (highlighted in red) in the candidate genes for triadimefon (Tri) resistance. **b** Phylogenetic tree of CCC1 homologous proteins. Amino acid sequences were retrieved from the NCBI database. Multiple sequence alignment was performed with MAFFT, and the phylogenetic tree was constructed using RAxML with the JTT+G4 model and 1000 bootstrap replicates. The conserved CCC1 domain is indicated by green boxes. **c** Amino acid alignment of the candidate triadimefon-resistance genes *PstCCC1.1* (*PstAZ2B06G00574*), *PstCCC1.2* (*PstAZ2B06G00575*), and *PstCCC1.3* (*PstAZ2B06G00587*), generated using T-Coffee. The coloring reflects conservation levels from high (red) to low (purple). The consensus line uses an asterisk (*) to denote fully conserved residues, a colon (:) for conservation between groups of strongly similar properties, and a period (.) for conservation between groups of weakly similar properties. The extent of the conserved CCC1 domain in each protein is demarcated by semi-transparent green boxes outlined with black dashed lines.

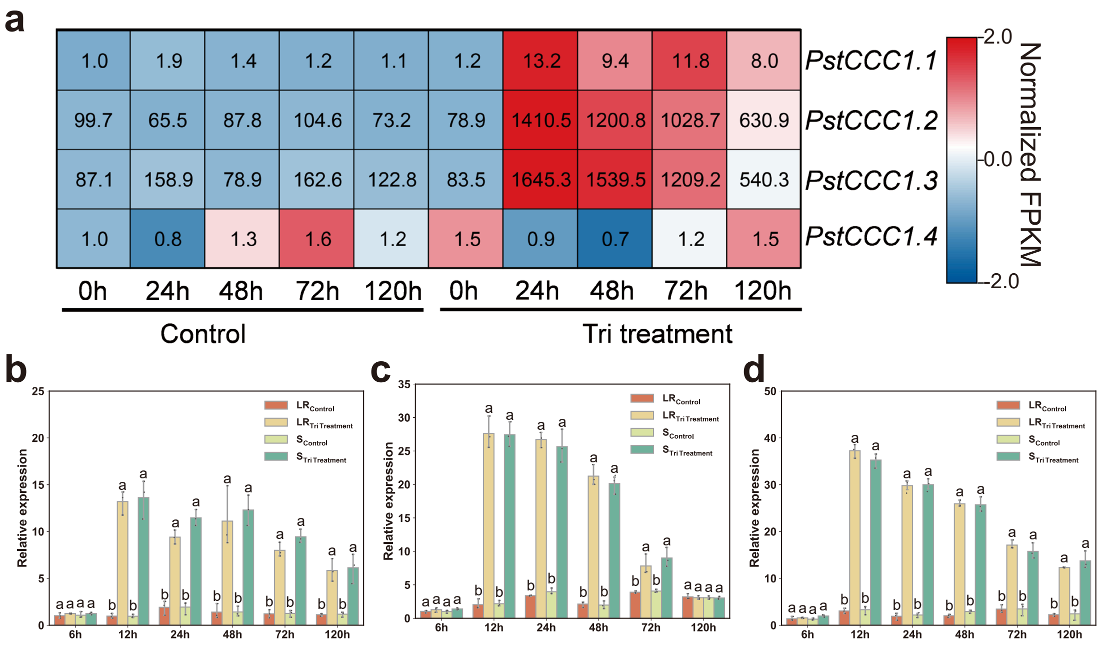

Supplementary Fig. 5. *PstCCC1.1*, *PstCCC1.2*, and *PstCCC1.3* exerts triadimefon-responsiveness in *Pst*. a Heatmap displaying the basal expression abundances of *PstCCC1.1*-*PstCCC1.4* in *Pst* following Tri treatment. The scale bar represents normalized FPKM value of these genes in *Pst* following Tri treatment. Tri treatment: hours after triadimefon application (0, 24, 48, 72, 120 h). Control: untreated samples at the corresponding time points. **b-d** Expression analysis of candidate triadimefon-resistance genes *PstCCC1.1*, *PstCCC1.2*, and *PstCCC1.3* in resistant (YQ324) and sensitive (Gui1-2) *Pst* isolates under triadimefon treatment. Wheat seedlings (cv. Mingxian 169) were inoculated with YQ324 and Gui1-2 urediniospores at 10 days after planting and treated with Tri at 3 days after *Pst* inoculation. Leaf samples were then collected at 0, 6, 12, 24, 48, 72, and 120 hours after Tri treatment for RNA-sequencing to determine the expression levels of the candidate genes. Expression levels were normalized based on the *Pst* elongation factor gene (*PstEF*). The relative expression values of triadimefon-resistance candidate genes were calculated using the comparative threshold method ($2^{-\Delta\Delta Ct}$). Data in **b–d** are mean ± SD ($n = 3$ replicates, each with 3 detached leaf segments). Statistical significance was assessed by one-way analysis of variance (ANOVA) with Duncan's new multiple range test (two-sided). Different letters denote significant differences at $P < 0.05$. Source data are provided as a Source Data file.

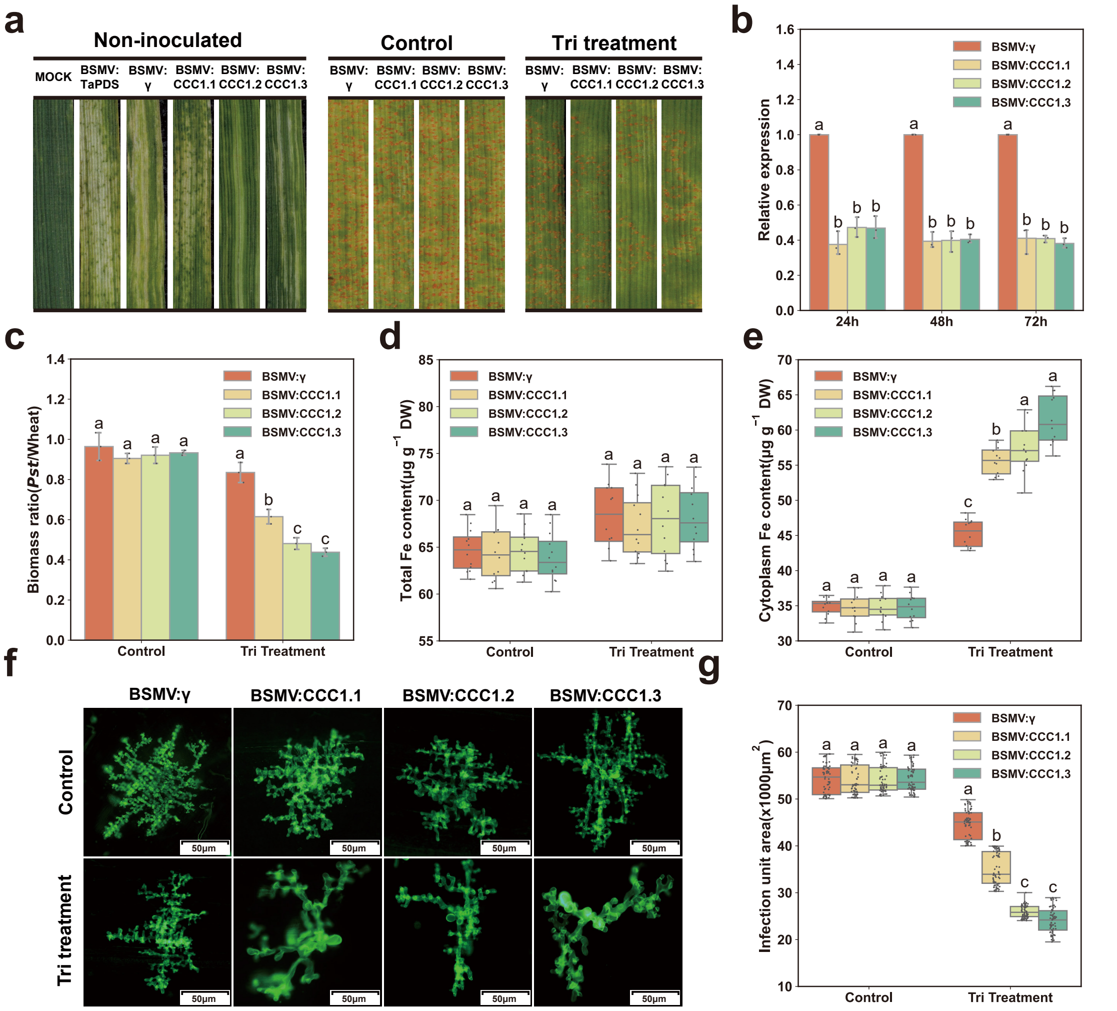

Supplementary Fig. 6. Silence of *PstCCC1.1*, *PstCCC1.2* and *PstCCC1.3* reduces the resistance of *Pst* to triadimefon by BSMV-HIGS. **a** Disease phenotype of low-resistant isolate YQ324 on the fourth leaves of Suwon 11 under 10 µg/mL Tri or water control. BSMV: TaPDS (phytoene desaturase) induces photobleaching, confirming silencing efficiency. BSMV: γ serves as empty vector control. **b** Silencing efficiency of *PstCCC1.1*, *PstCCC1.2*, and *PstCCC1.3* was measured in HIGS plants at 24, 48, and 72 hours after *Pst* inoculation, prior to triadimefon treatment. The data represent the transcript levels relative to those in the BSMV: γ control plants at the same time point. **c** Statistical analysis of *Pst* biomass from wheat leaves treated with Tri in comparison to water control, assessed 10 days post-treatment. *TaEF* and *PstEF* were used to normalize the RNA level of wheat leaves and *Pst*, respectively. **d, e** Total iron (**d**) and cytosolic iron (**e**) content in *Pst* hyphae collected from *Pst*-inoculated HIGS plants treated with Tri and water (control). **f** Fluorescence microscopy observation of mycelial growth of *Pst* in wheat leaves after silencing of resistance candidate genes after 72 hpi of the triadimefon treatment and water control. WGA-Alexa488 (excitation/emission: 495/519 nm) was used for staining. Scale bar = 50 µm. **g** Comparison of the mycelial area of each infected unit in wheat leaves treated with triadimefon and water (control) at 72 hpi. Data in **b**, **c** are mean ± SD (*n* = 3 replicates, each with 3 detached leaf segments). For box

plots in **d**, **e**, and **g**, boxes represent the interquartile range (25th to 75th percentiles), center lines indicate medians, and whiskers extend to 1.5 × IQR. Data in **d**, **e** are mean ± SD (*n* = 12 replicates, each from 3 detached leaf segment). Data in **g** are mean ± SD (*n* = 3 replicates, each with 60 infection sites per replicate). Statistical significance was determined by one-way analysis of variance (ANOVA) with Duncan's new multiple range test (two-sided). Different letters denote significant differences at $P < 0.05$. Source data are provided as a Source Data file.

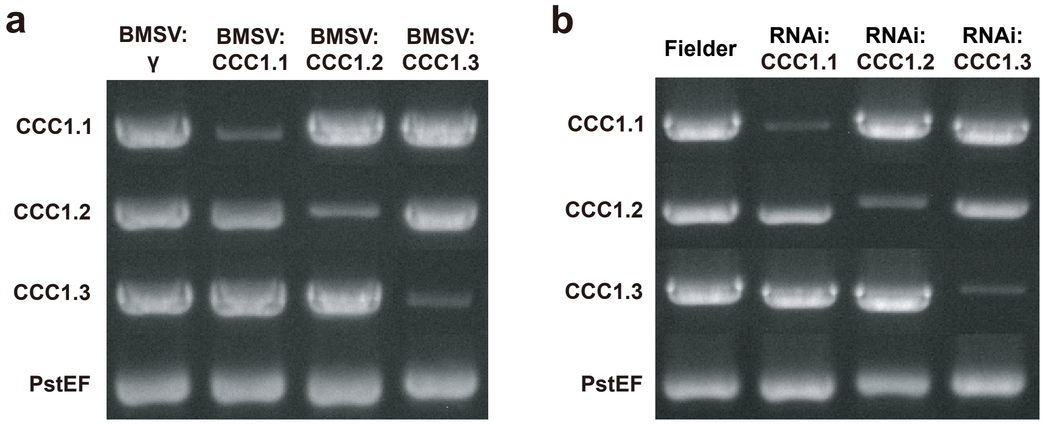

Supplementary Fig. 7. Semi-quantitative RT-PCR analysis of *PstCCC1.1*, *PstCCC1.2*, and *PstCCC1.3* expression following HIGS and RNAi-mediated silencing. **a** Expression analysis of *PstCCC1.1*, *PstCCC1.2*, and *PstCCC1.3* in the *Pst* isolate YQ342 after host-induced gene silencing (HIGS). The negative control sample (BMSV: γ) was infiltrated with a vector not targeting any genes. The test samples were infiltrated with vectors for transient silencing of *PstCCC1.1*, *PstCCC1.2*, or *PstCCC1.3*, as indicated. Gene expression of all three *PstCCC1* genes were assessed in each silencing condition. **b** Expression analysis of *PstCCC1.1*, *PstCCC1.2*, and *PstCCC1.3* in the *Pst* isolate YQ342 during infection of RNAi wheat lines. The negative control sample (Fielder) is the wild-type cultivar with no gene silencing. The test samples were from plants with transient silencing of *PstCCC1.1*, or stable silencing of *PstCCC1.2* or *PstCCC1.3*, as indicated. Gene expression of all three *PstCCC1* genes were assessed in each condition. *PstEF* was used as the internal reference gene for all samples.

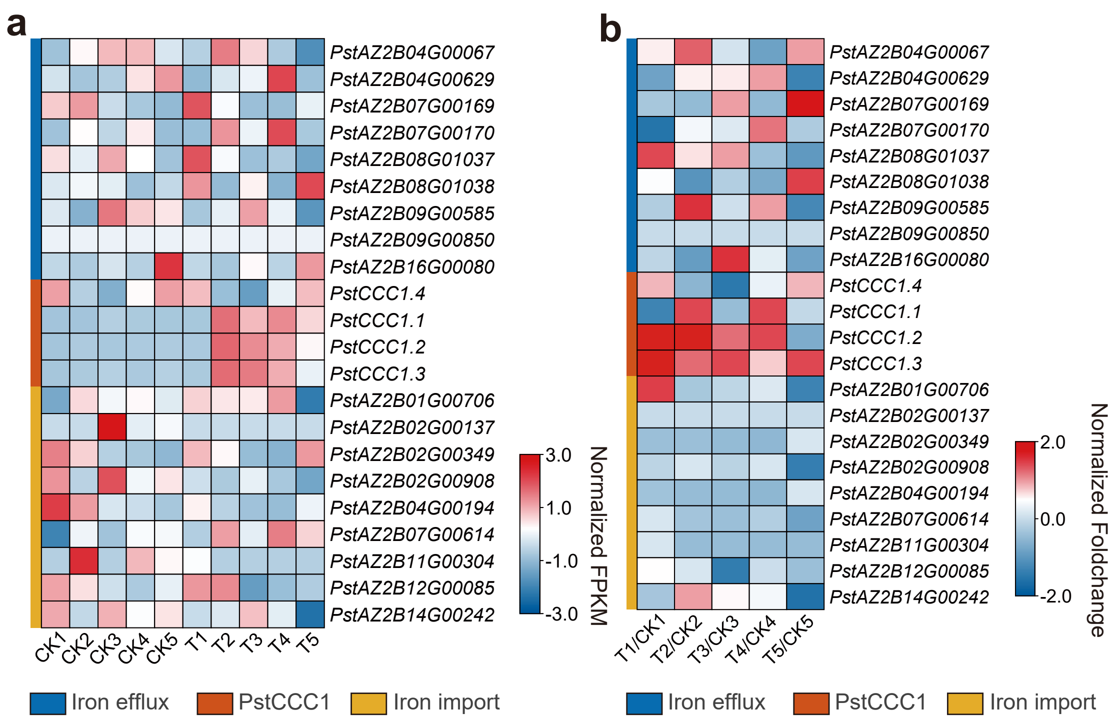

Supplementary Fig. 8. Transcription abundances of *PstCCC1* and genes responsible for iron import or efflux in *Pst* under experimental conditions. a Basal expression abundance of these genes in *Pst* following triadimefon treatment. Scale bar represents the normalized FPKM values in *Pst* following triadimefon treatment. **b** Heatmap displaying the foldchange values of these genes in each comparison. T1-T5: time points at 0, 24, 48, 72, and 120 hours after triadimefon treatment.CK1-CK5: corresponding untreated control samples collected at the same time points as T1-T5, respectively. All samples were taken starting at 72 hours post-inoculation

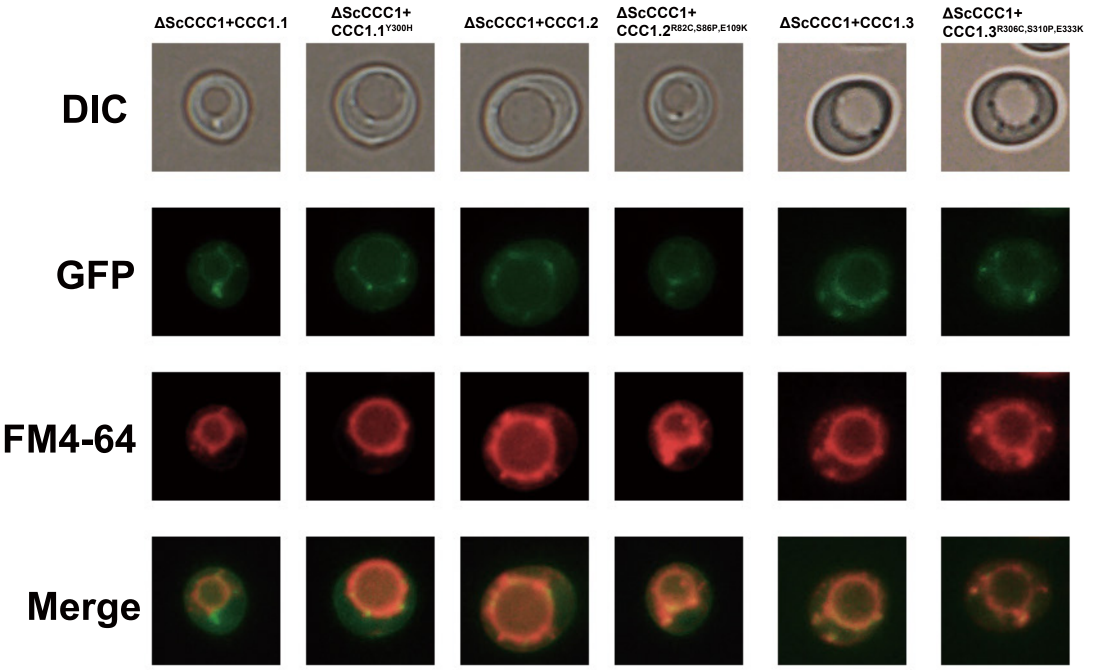

Supplementary Fig. 9 Subcellular localization of candidate resistance genes and their mutants in yeast cells. All signals were monitored using fluorescence microscope. FM4-64 (excitation/emission: 515/640 nm) was used to label the plasma membrane. The mCherry channel (excitation/emission: 587/610 nm) was used and the excitation wavelength was set to 599 nm. Green fluorescence protein (GFP) is in green and the excitation wavelength was set to 488 nm. Bright field images show the equivalent field observed under white light. Comparable expression and localization patterns were observed in three independent biological replicates.

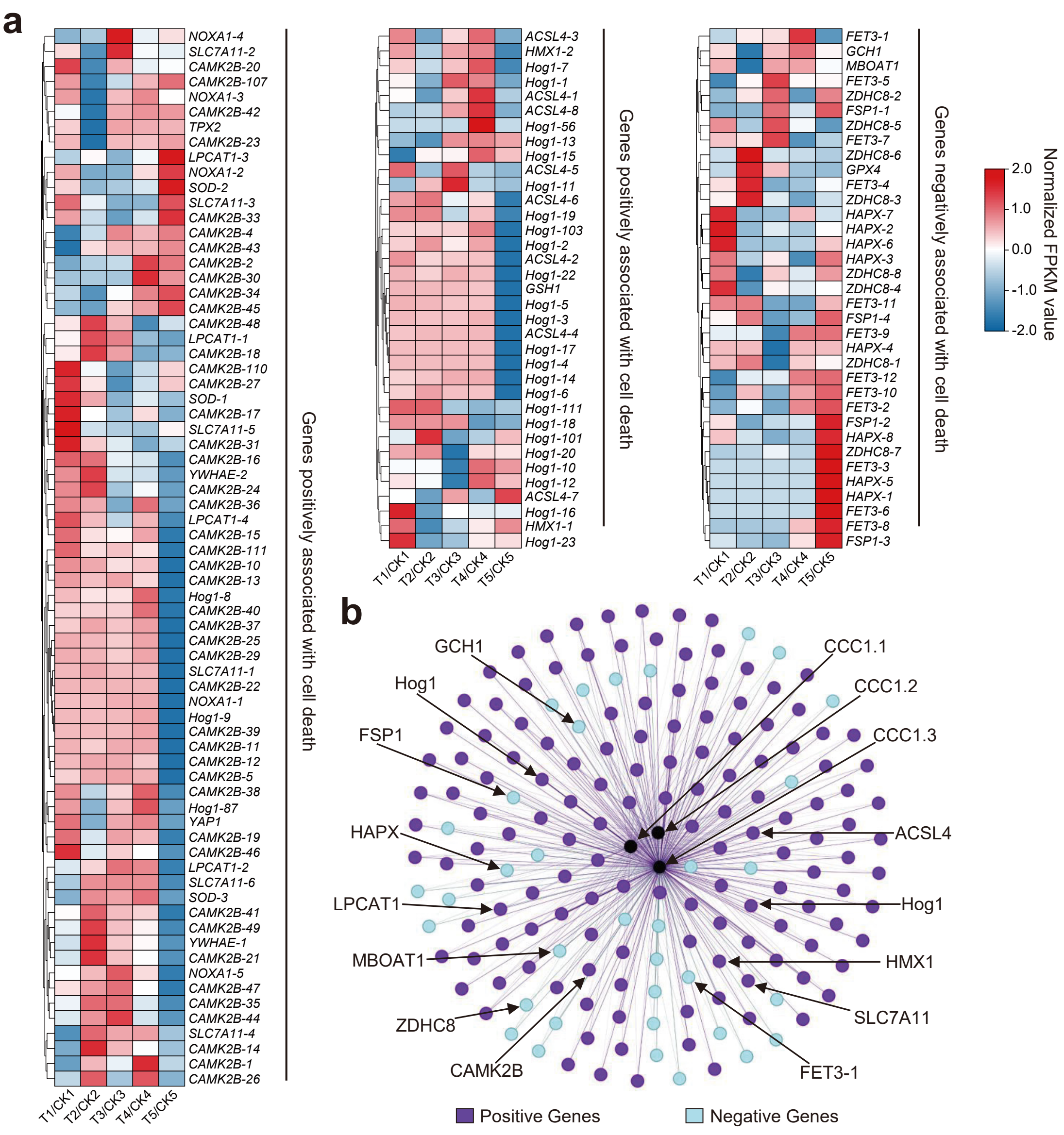

Supplementary Fig. 10. Triadimefon triggered the expression of genes involved in cell death in *Pst*. a Heat map displaying the expression abundances of cell death promoting and inhibitory genes in *Pst* following triadimefon. The high and low expression abundances of related genes are shown in red and blue, respectively. The scale bar represents the relative expression levels of genes. The genes involved in cell death are identified in *Pst* using via homology identification of functional genes that have been well documented in cell death of mouse, *Saccharomyces cerevisiae* and *Aspergillus fumigatus* (Supplementary Table 7). T1-T5: time points at 0, 24, 48, 72, and 120 hours after triadimefon treatment. CK1-CK5: corresponding untreated control samples collected at the same time points as T1-T5, respectively. All samples were taken starting at 72 hours post-inoculation. **b** Correlation network of *PstCCC1* and genes relevant to cell death. The correlation

relationship between *PstCCC1* and genes relevant to cell death are calculated using Pearson algorithm based on their expression abundances in each experimental group. The positive and negative relationships are labeled by purple and blue lines, respectively. The cell death promoting genes were shown in purple and inhibitory genes in blue.

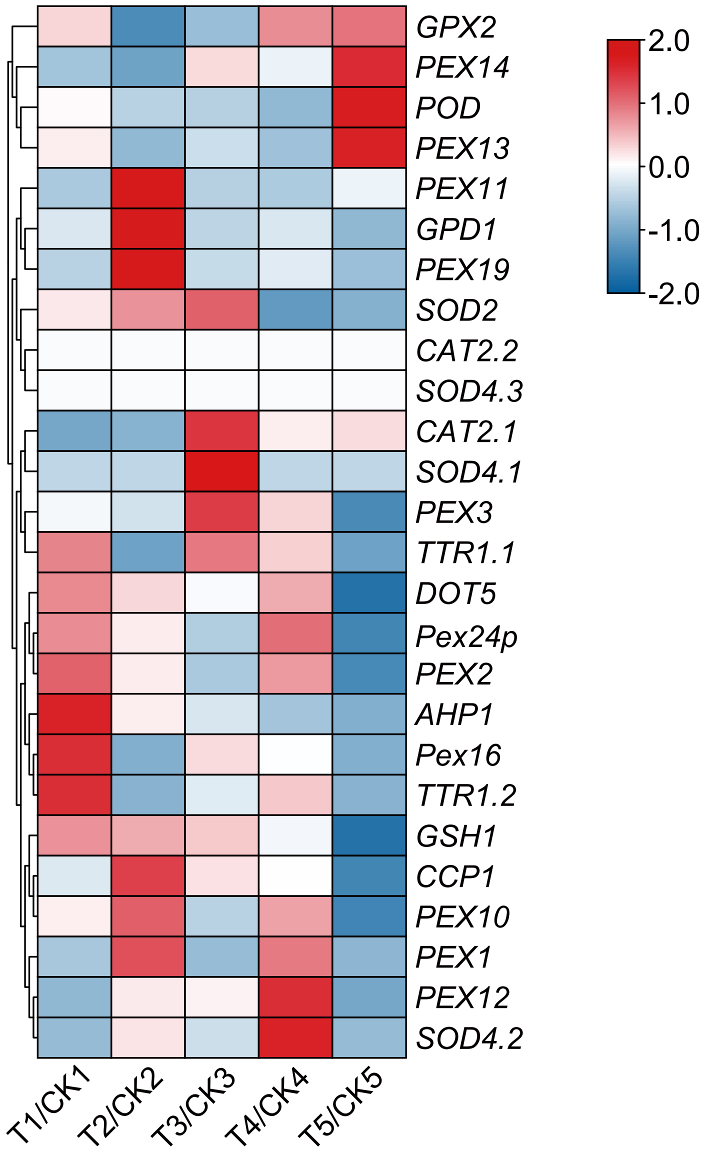

Supplementary Fig. 11. Relative expression levels of genes encoding antioxidant enzymes in *Pst* following triadimefon treatment. The scale bar represents the normalized foldchange values of representative antioxidant genes in *Pst* following triadimefon treatment, showing that triadimefon treatment induced upregulations of numerous antioxidant genes, and the effects are gradually decreased following the triadimefon treatment time. T1-T5: time points at 0, 24, 48, 72, and 120 hours after triadimefon treatment. CK1-CK5: corresponding untreated control samples collected at the same time points as T1-T5, respectively. All samples were taken starting at 72 hours post-inoculation.

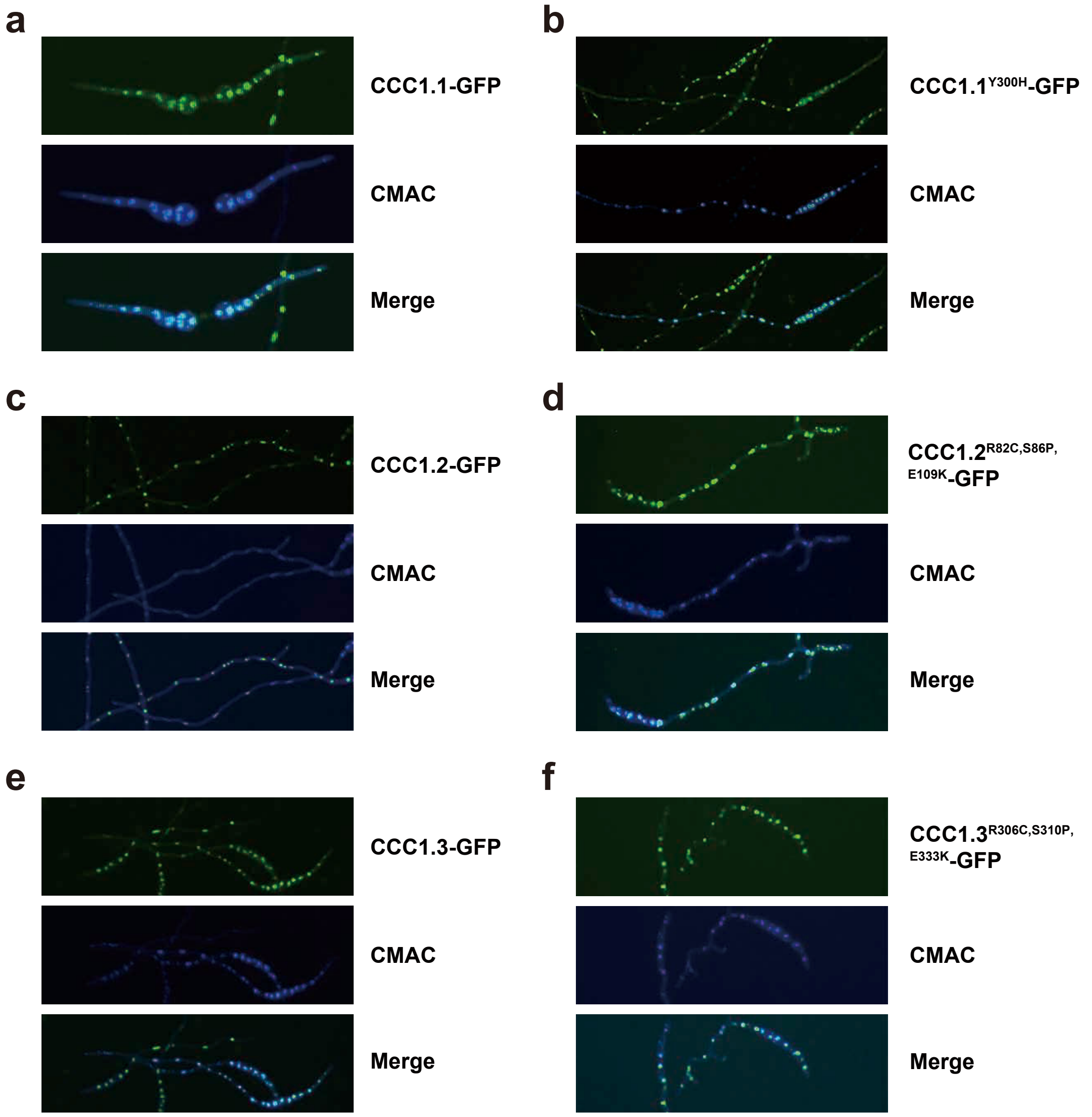

Supplementary Fig. 12. Subcellular localization of candidate resistance genes and their mutants in ΔFgCCC1 mutants. All signals were monitored using a fluorescence microscope. The 7-amino-4-chloromethylcoumarin, L-arginine amide (CMAC) (excitation/emission: 354/466 nm) was used for vacuolar staining with a blue signal, and a wavelength of 350 nm is set using a UV channel. Green fluorescence (GFP) is in green and the excitation wavelength was set to 488 nm. Comparable expression and localization patterns were observed in three independent biological replicates.

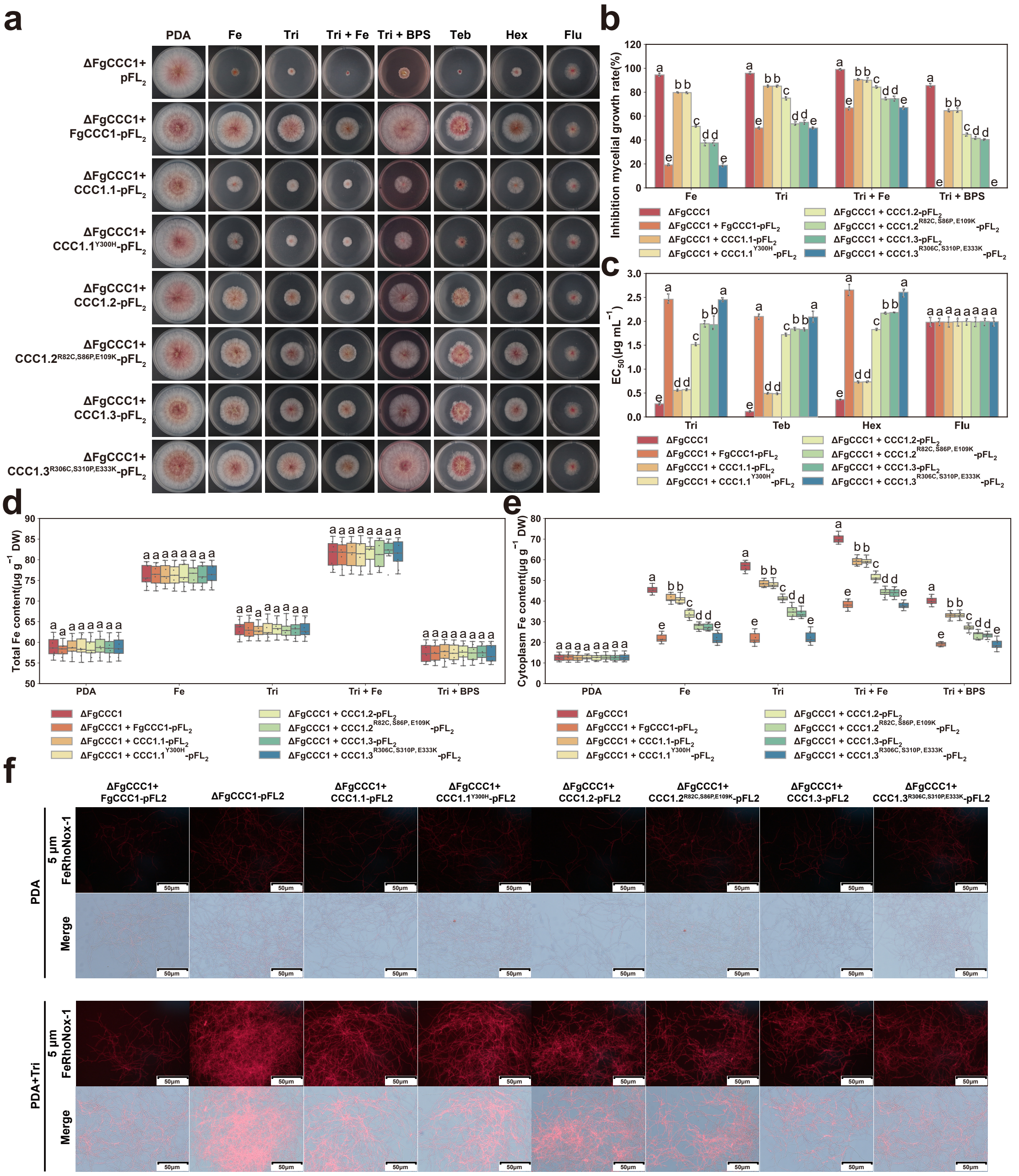

Supplementary Fig. 13. Heterologous expression of *PstCCC1* genes and their mutants in ΔFgCCC1 reveals their role in triadimefon sensitivity and iron homeostasis. a Phenotypic analysis of *Fusarium graminearum* strains grown for 3 days on potato dextrose agar (PDA) under different conditions: normal PDA, PDA supplemented with 2 mM $FeSO_4$, PDA with 2.5 μg/mL Tri, PDA with 2 mM $FeSO_4$ and 2.5 μg/mL Tri, PDA with 2.5 μg/mL Tri and 0.1 mM BPS, PDA with 2.5 μg/mL Teb, PDA with 2.5 μg/mL Hex, and PDA with 2.5 μg/mL Flu. Strains include *ΔFgCCC1*, complemented strain ΔFgCCC1+FgCCC1, and ΔFgCCC1 expressing *PstCCC1.1*, *PstCCC1.2*, and *PstCCC1.3* or mutant versions. **b** Relative inhibition rate (%) of mycelial growth under four treatment conditions: PDA + 2 mM $FeSO_4$, PDA + 2.5 μg/mL Tri, PDA + 2 mM $FeSO_4$ + 2.5 μg/mL Tri, and PDA + 2.5 μg/mL Tri + 0.1 mM BPS. Inhibition rates were calculated relative to the mycelial diameter of untreated controls for each strain**. c** Comparison of EC_{50} values for Tri, Teb,

Hex, and Flu among the transgenic and control strains. **d, e** Total iron (**d**) and cytosolic iron (**e**) content measured by ICP-MS in mycelia grown under four conditions: normal PDA, PDA + 2 mM $FeSO_4$, PDA + 2.5 μg/mL Tri, and PDA + 2 mM $FeSO_4$ + 2.5 μg/mL Tri. For box plots, boxes represent the interquartile range (25th to 75th percentiles), center lines indicate medians, and whiskers extend to 1.5 × IQR. **f** Iron content in whole cell of the wild type, ΔFgCCC1 and heterologous overexpression mutant of *PstCCC1* was determined by a laser scanning microscope with 5 μM fluorescent iron-binding dye FeRhoNox-1 (excitation/emission: 488/523 nm). Data in **b**, **c** are mean ± SD (n = 3 replicates). Data in **d**, **e** are mean ± SD (n = 9 replicates, each with mycelia pooled from 3 petri dishes). Statistical significance was determined by one-way analysis of variance (ANOVA) with Duncan's new multiple range test (two-sided). Different letters above the bars denote significant differences at $P < 0.05$. Source data are provided as a Source Data file.

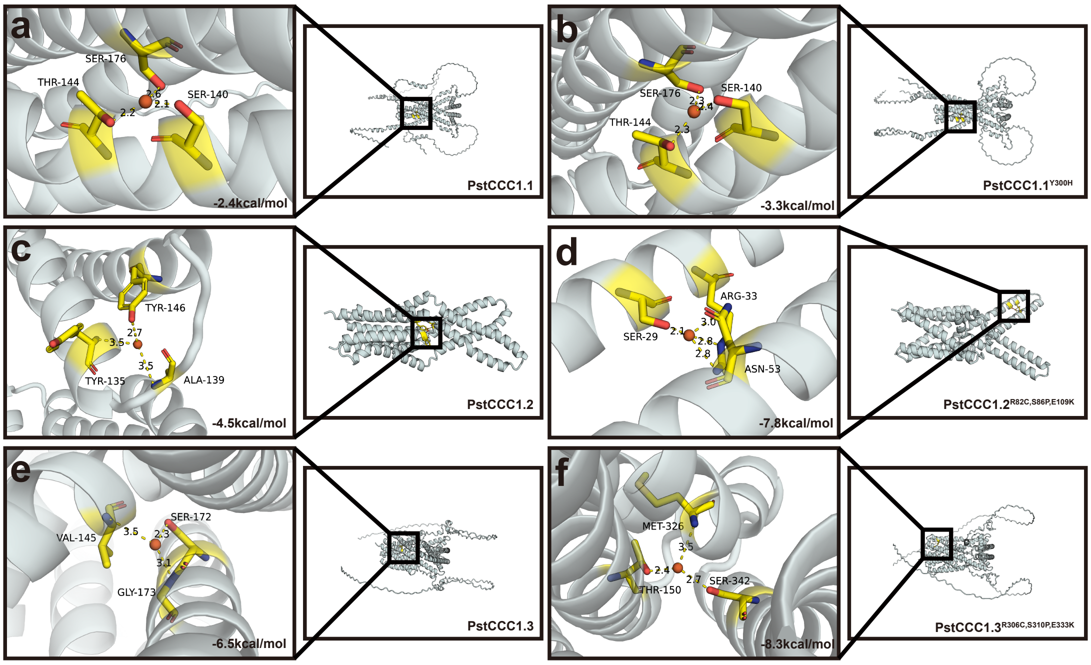

Supplementary Fig. 14. Molecular docking of vacuolar iron transporters and their mutants. **a, c, e** Molecular docking of iron ion with PstCCC1.1, PstCCC1.2 and PstCCC1.3. **b, d, f** Molecular docking of iron ion with PstCCC1.1^{Y300H}, PstCCC1.2^{R82C,S86P,E109K} and PstCCC1.3^{R306C,S310P,E333K}, with an enlarged view of the binding site detailing H-bond interactions (distances indicated in magenta).

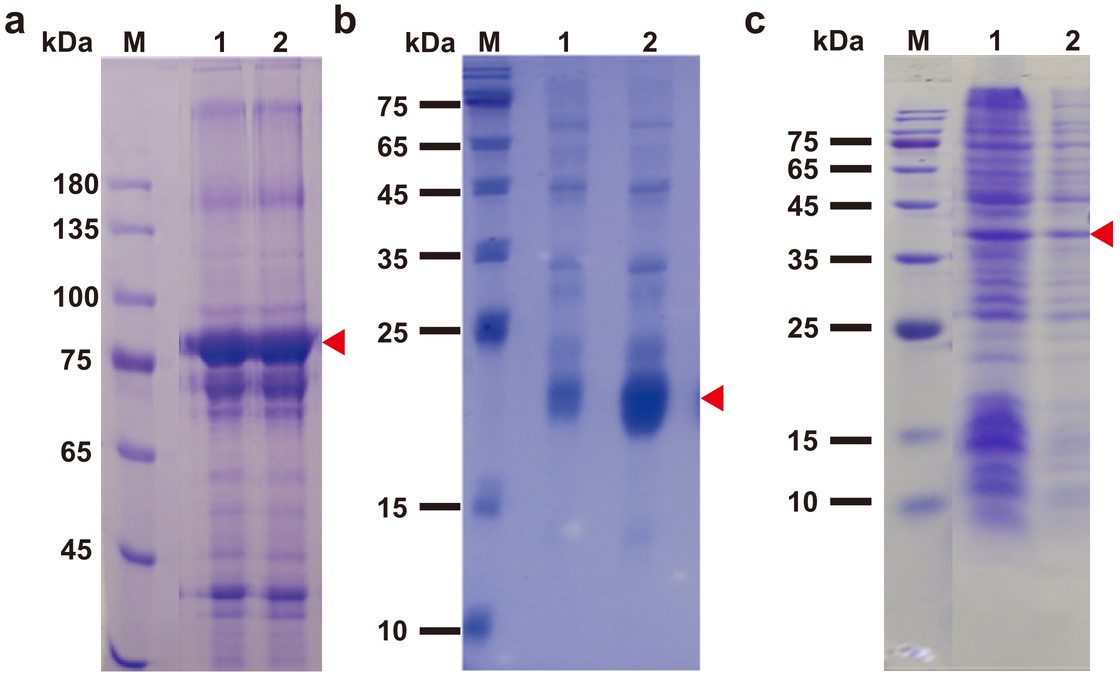

Supplementary Fig. 15. Sodium dodecyl sulfate-polyacrylamide gel electrophoresis (SDS-PAGE) of purified proteins. **a** Lanes 1 and 2 represent PstCCC1.1 and PstCCC1.1^{Y300H}, respectively. **b** Lanes 1 and 2 represent PstCCC1.2 and PstCCC1.2^{R82C,S86P,E109K}, respectively. **c** Lanes 1 and 2 represent PstCCC1.3 and PstCCC1.3^{R306C,S310P,E333K}, respectively.

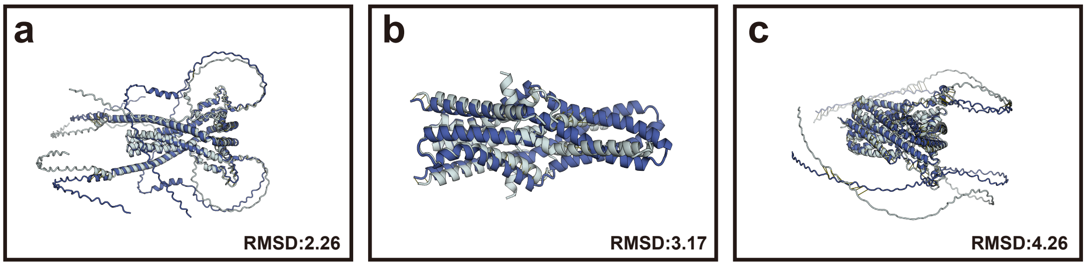

Supplementary Fig. 16. Superimposition of the vacuolar iron transporters and their mutants. **a** The superimposition of the PstCCC1.1 structures, showing the rotation of the heterocyclic ring that occurs when Y300 is mutated to histidine. **b** The superimposition of the PstCCC1.2 structures, showing the rotation of the heterocyclic ring that occurs when R82C, S86 and E109K are mutated to cysteine, proline, and lysine, respectively. **c** The superimposition of the PstCCC1.3 structures, showing the rotation of the heterocyclic ring that occurs when R306C, S310P and E333K are mutated to cysteine, proline, and lysine, respectively. Note: Structural superimpositions were evaluated by Root Mean Square Deviation (RMSD; measured in Ångströms, with lower values indicating higher structural precision).

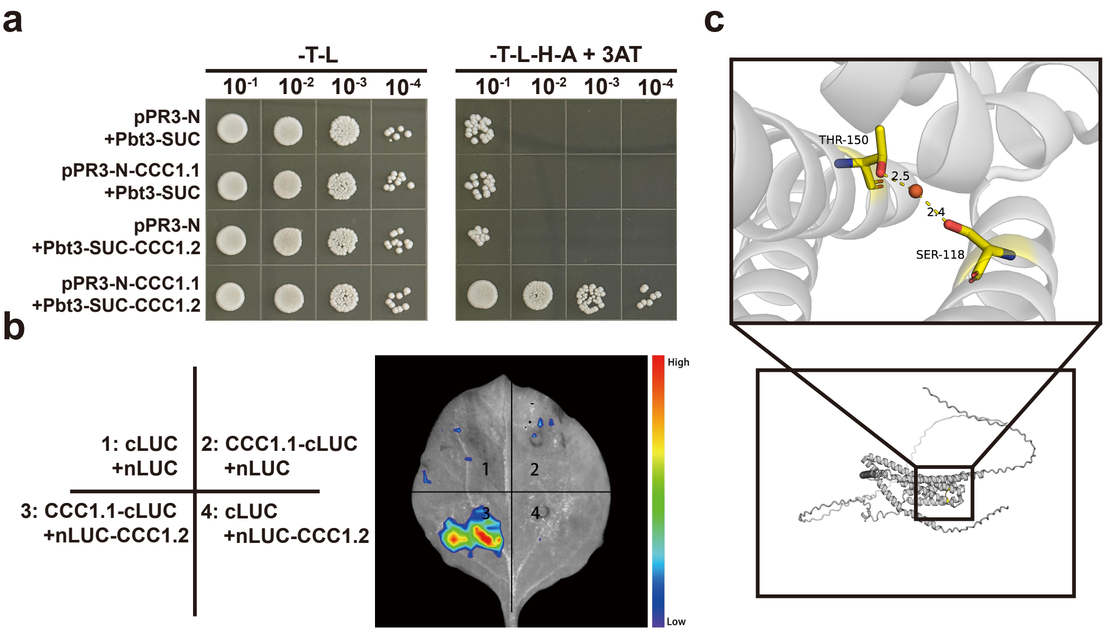

Supplementary Fig. 17. The interaction between PstCCC1.1 and PstCCC1.2 synergistically increases their ferrous iron-binding ability beyond their individual capacities. **a** Yeast transformants expressing the labeled constructs of PstCCC1.1 and PstCCC1.2 of *Puccinia striiformis* f. sp. *tritici* were assayed for growth on Synthetic Dropout Medium without Tryptophan and Leucine (SD-TL) or Synthetic Dropout Medium without Lysine, Leucine, Histidine, and Adenine (SD-LLHA) with 3-Amino-1,2,4-triazole (3AT). **b** Split luciferase complementation assay in *Nicotiana benthamiana* showing that PstCCC1.1 interacts with PstCCC1.2. Only co-agroinfiltration with cLuc-PstCCC1.1 and nLuc-PstCCC1.2 was detected luminescence, whereas nLuc-PstCCC1.2 and cLuc, cLuc-PstCCC1.1 and nLuc, or cLuc and nLuc were not. Luminescence was detected by spraying the leaves with beetle luciferin and observed by cooled Charge-Coupled Device (CCD) imaging. **c** PstCCC1.1-PstCCC1.2 interaction modeling and iron molecular docking, with an enlarged view of the binding site detailing H-bond interactions (distances indicated in magenta).

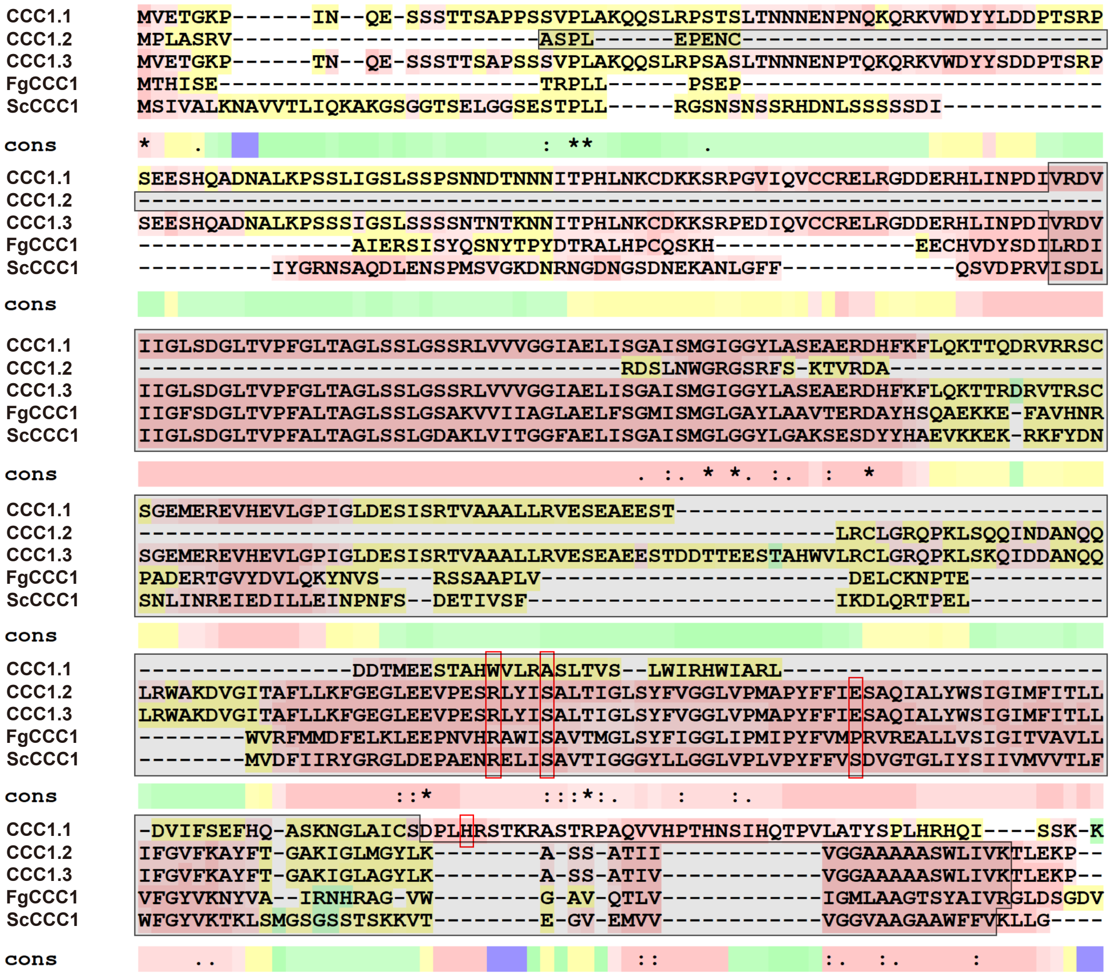

Supplementary Fig. 18. Analysis of the conservation of mutation sites in *Puccinia striiformis* CCC1 (CCC1.1, CCC1.2, CCC1.3) relative to their orthologs in *Fusarium graminearum* (FgCCC1) and *Saccharomyces cerevisiae* (ScCCC1). The amino acid sequence alignment was generated using T-Coffee. Residue coloring reflects conservation levels from high (red) to low (purple). The consensus line uses an asterisk (*) for fully conserved residues, a colon (:) for conservation between groups of strongly similar properties, and a period (.) for conservation between groups of weakly similar properties. The conserved CCC1 domain in each protein is demarcated by a semi-transparent gray area, and the specific mutation sites are highlighted within red boxes.

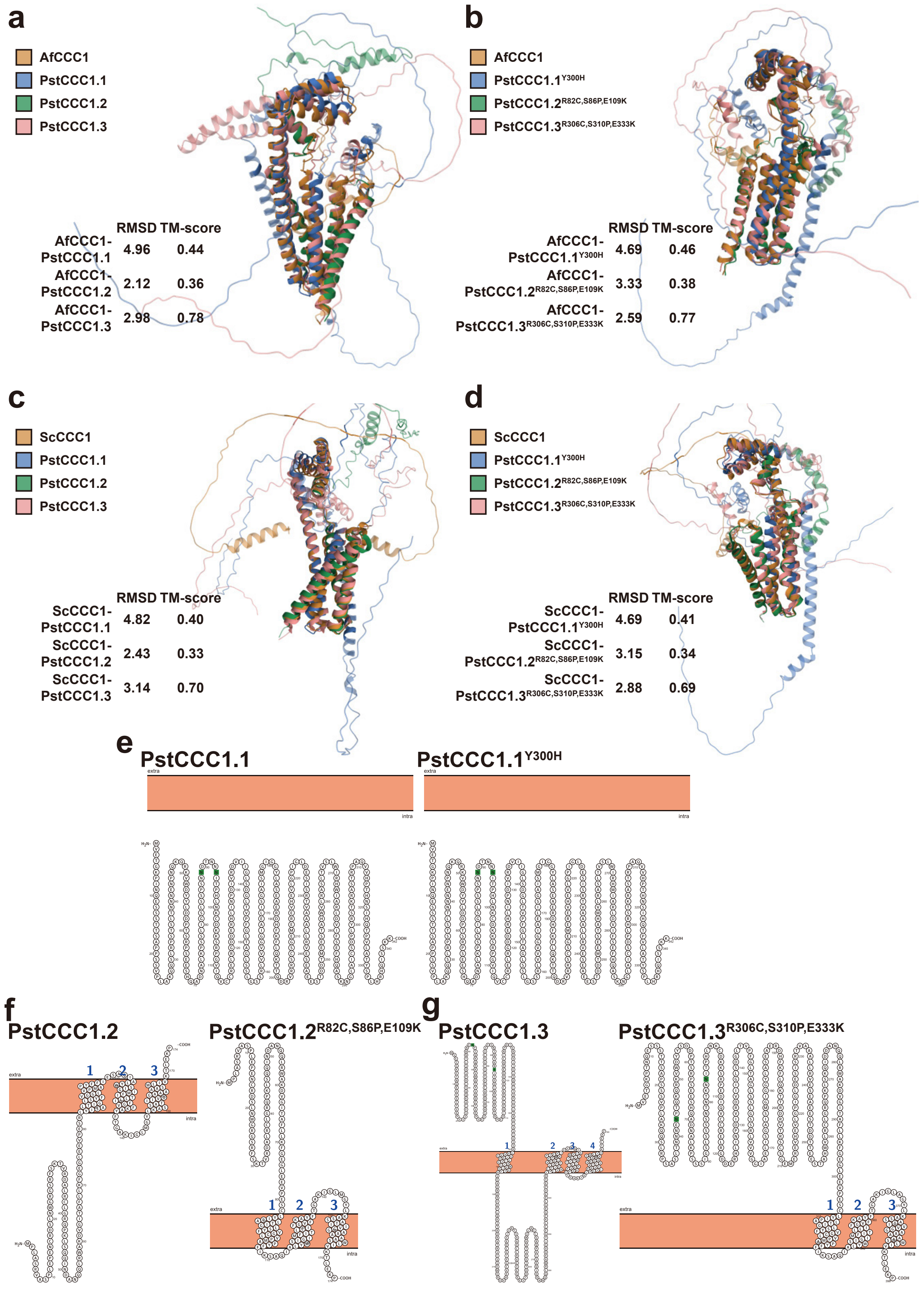

Supplementary Fig. 19. Natural mutation of key functional sites causes changes in structure and transmembrane (TM) domains of PstCCC1.1-PstCCC1.3. a-d Structural comparative analysis of the *PstCCC1* before and after natural mutation with AfCCC1 and ScCCC1. The structural comparative analysis is performed using PDB database based on T-m score (ranging from 0 to 1, assessing overall fold similarity; higher values indicate greater similarity) and RMSD

(measured in Ångströms, measuring local atomic positional deviation; lower values indicate higher precision). The proteins are shown in different colors, and visualized using Pymol. **e-g** Predication about transmembrane domain of PstCCC1.1-PstCCC1.3 before and after natural mutation using PROTTER predictor. The natural mutation results in changes in transmembrane domain and extracellular peptide segments of PstCCC1, especially PstCCC1.3 and PstCCC1.2.
