# Supplementary material for: Vacuolar iron transporters mediate resistance to triadimefon in plant pathogenic fungi
Source: Nat Commun. 2026 May 6;17:6379. doi: 10.1038/s41467-026-72157-6 (PMC13376413; doi:10.1038/s41467-026-72157-6)
Supplement: Supplementary file 2 — Description of Additional Supplementary Files [file 41467_2026_72157_MOESM2_ESM.pdf]

# Description of Additional Supplementary Files

**File Name:** Supplementary Data 1

**Description:** Triadimefon sensitivity of $F_1$ progeny isolates of *Puccinia striiformis* f. sp. *tritici* measured by half effective concentration ($EC_{50}$), toxicity regression equation, $R^2$ and resistance index (RI).

**File Name:** Supplementary Data 2

**Description:** Triadimefon sensitivity of $F_2$ progeny isolates of *Puccinia striiformis* f. sp. *tritici* measured by half effective concentration ($EC_{50}$), toxicity regression equation, $R^2$ and resistance index (RI).

**File Name:** Supplementary Data 3

**Description:** Half effective concentration ($EC_{50}$) values of the resistant parental (RP) isolate YQ324 and the sensitive parental (SP) isolate Gui1-2 of *Puccinia striiformis* f. sp. *tritici* and their progeny populations and the segregation in the $F_2$ population for sensitivity to triadimefon.

**File Name:** Supplementary Data 4

**Description:** Functional annotation of genes within the QTL region on chromosome 6.

**File Name:** Supplementary Data 5

**Description:** Annotation of genes in *Puccinia striiformis* f. sp. *tritici* on chromosomes 6 and 11 related to triadimefon resistance.

**File Name:** Supplementary Data 6

**Description:** Primers used in this study.

**File Name:** Supplementary Data 7

**Description:** Genes associated with cell death.
