# Supplementary material for: Vacuolar iron transporters mediate resistance to triadimefon in plant pathogenic fungi
Source: Nat Commun. 2026 May 6;17:6379. doi: 10.1038/s41467-026-72157-6 (PMC13376413; doi:10.1038/s41467-026-72157-6)
Supplement: Supplementary file 4 — Reporting Summary [file 41467_2026_72157_MOESM4_ESM.pdf]

nature portfolio

Corresponding author(s): Gang-ming Zhan

Last updated by author(s): Mar 29, 2026

Reporting Summary

Nature Portfolio wishes to improve the reproducibility of the work that we publish. This form provides structure for consistency and transparency in reporting. For further information on Nature Portfolio policies, see our Editorial Policies and the Editorial Policy Checklist.

Statistics

For all statistical analyses, confirm that the following items are present in the figure legend, table legend, main text, or Methods section.

n/a	Confirmed	
☐	☒	The exact sample size (*n*) for each experimental group/condition, given as a discrete number and unit of measurement
☐	☒	A statement on whether measurements were taken from distinct samples or whether the same sample was measured repeatedly
☐	☒	The statistical test(s) used AND whether they are one- or two-sided<br>*Only common tests should be described solely by name; describe more complex techniques in the Methods section.*
☒	☐	A description of all covariates tested
☒	☐	A description of any assumptions or corrections, such as tests of normality and adjustment for multiple comparisons
☐	☒	A full description of the statistical parameters including central tendency (e.g. means) or other basic estimates (e.g. regression coefficient) AND variation (e.g. standard deviation) or associated estimates of uncertainty (e.g. confidence intervals)
☐	☒	For null hypothesis testing, the test statistic (e.g. *F*, *t*, *r*) with confidence intervals, effect sizes, degrees of freedom and *P* value noted<br>*Give P values as exact values whenever suitable.*
☒	☐	For Bayesian analysis, information on the choice of priors and Markov chain Monte Carlo settings
☒	☐	For hierarchical and complex designs, identification of the appropriate level for tests and full reporting of outcomes
☐	☒	Estimates of effect sizes (e.g. Cohen's *d*, Pearson's *r*), indicating how they were calculated

Our web collection on statistics for biologists contains articles on many of the points above.

Software and code

Policy information about availability of computer code

Data collection	BSA-seq were sequenced by Illumina HiSeq 2500 at Novogene Bioinformatics Institute (China). RNA-seq libraries were sequenced with Illumina HiSeq 2500 at Guangzhou GENE DENOVO Technologies (China). Quantitative reverse transcription polymerase chain reaction (qRT-PCR) assays were performed using the CFX96 Real-Time System (Bio-RAD, USA).
Data analysis	The quality of the BSA-seq raw sequencing data was evaluated and filtered using software Illumina Casava 1.8 version 1.2.3 (https://support-docs.illumina.com/SW/ClarityLIMS/ClarityINT/Content/SW/ClarityLIMS/Integrations/CASAVA182SampleSheet.htm). After completing the quality assessment of sequencing data, the clean sequencing reads were mapped using the Pst AZ2 genome as the reference (https://doi.org/10.6084/m9.figshare.24265198.v5) and software BWA (parameter: mem-t4-k32-M), and the alignment results were deduplicated using SAMTOOLS (parameter: rmdup). ΔSNP-index and 0mED values were calculated using 200000-bp windows and 20000-bp step sizes, retaining windows with SNP counts ≥20. The mapped reads of RNA-seq were assembled using StringTie v1.3.1 in a reference-based approach. For each transcription region, a FPKM (fragment per kilobase of transcript per million mapped reads) value was calculated to quantify its expression abundance and variations, using the RSEM software. Principal component analysis (PCA) of the RNA sequences was performed with the R package (http://www.r-project.org/). Differentially expressed genes (DEGs) were analyzed using the R package DESEQR (v.3.7.1) and then used in the Kyoto Encyclopedia of Genes and Genomes (KEGG) and Gene Ontology (GO) enrichment analyses with the R package CLUSTERPROFILER (P<0.05, FOLDCHANGE > 2.0).

For manuscripts utilizing custom algorithms or software that are central to the research but not yet described in published literature, software must be made available to editors and reviewers. We strongly encourage code deposition in a community repository (e.g. GitHub). See the Nature Portfolio guidelines for submitting code & software for further information.

Data

Policy information about availability of data

All manuscripts must include a data availability statement. This statement should provide the following information, where applicable:

- Accession codes, unique identifiers, or web links for publicly available datasets
- A description of any restrictions on data availability
- For clinical datasets or third party data, please ensure that the statement adheres to our policy

We have provided a full data availability statement in the manuscript. The BSA-seq and transcriptomic data generated in this study have been deposited in the China National GeneBank DataBase (CNGBdb) under accession codes CNP0007785 [https://db.cngb.org/data_resources/project/CNP0007785/] and CNP0007780 [https://db.cngb.org/data_resources/project/CNP0007780/]. The Pst AZ2 reference genome used in this study is available in the NCBI database under accession code GCA_039519225.1 [https://www.ncbi.nlm.nih.gov/datasets/genome/GCA_039519225.1/]. The detailed sequence information for genes PstCCC1.1 (PstAZ2B06G00574), PstCCC1.2 (PstAZ2B06G00575) and PstCCC1.3 (PstAZ2B06G00587) including identified allelic variations, are provided in the Supplementary Information file. Source data are provided with this paper.

Research involving human participants, their data, or biological material

Policy information about studies with human participants or human data. See also policy information about sex, gender (identity/presentation), and sexual orientation and race, ethnicity and racism.

Reporting on sex and gender	NA
Reporting on race, ethnicity, or other socially relevant groupings	NA
Population characteristics	NA
Recruitment	NA
Ethics oversight	NA

Note that full information on the approval of the study protocol must also be provided in the manuscript.

Field-specific reporting

Please select the one below that is the best fit for your research. If you are not sure, read the appropriate sections before making your selection.

☒ Life sciences ☐ Behavioural & social sciences ☐ Ecological, evolutionary & environmental sciences

For a reference copy of the document with all sections, see nature.com/documents/nr-reporting-summary-flat.pdf

Life sciences study design

All studies must disclose on these points even when the disclosure is negative.

Sample size	No statistical method was used to predetermine sample size. The specific sample size for each experiment is delineated in the methods section and Figure legends. For HIGS and RNAi experiments, 3 biological replicates were sampled, each with 3 detached leaf segments (refs. 40, 50, 54). For Pst fungicide sensitivity assays, 3 biological replicates were used, each with 5 detached leaf segments (refs. 8, 44). For Pst iron content measurements, 12 or 18 biological replicates were used, each with 3 detached leaf segments, which was determined to be sufficient based on preliminary trials. For Fusarium graminearum assays, 9 independent replicates were performed, each with mycelia pooled from 3 petri dishes (refs. 16, 57). For yeast-based assays, 3 independent replicates were used (refs. 60, 61). The sample sizes and material amount were determined based on experimental trials to allow for confident statistical analyses.
Data exclusions	No data were excluded from the analyses.
Replication	All attempts at replication were successful. The number of replicates is indicated in the corresponding figure legend and/or in the corresponding material and method section.
Randomization	Randomization was used for all the biological experiments.
Blinding	No blinding was done as none of the experiments described in this study involve group allocation during data collection or analyses.

Reporting for specific materials, systems and methods

We require information from authors about some types of materials, experimental systems and methods used in many studies. Here, indicate whether each material, system or method listed is relevant to your study. If you are not sure if a list item applies to your research, read the appropriate section before selecting a response.

Materials & experimental systems

n/a	Involved in the study
☒	☐ Antibodies
☒	☐ Eukaryotic cell lines
☒	☐ Palaeontology and archaeology
☒	☐ Animals and other organisms
☒	☐ Clinical data
☒	☐ Dual use research of concern
☐	☒ Plants

Methods

n/a	Involved in the study
☒	☐ ChIP-seq
☐	☒ Flow cytometry
☒	☐ MRI-based neuroimaging

Dual use research of concern

Policy information about dual use research of concern

Hazards

Could the accidental, deliberate or reckless misuse of agents or technologies generated in the work, or the application of information presented in the manuscript, pose a threat to:

No	Yes
☒	☐ Public health
☒	☐ National security
☒	☐ Crops and/or livestock
☒	☐ Ecosystems
☒	☐ Any other significant area

Experiments of concern

Does the work involve any of these experiments of concern:

No	Yes
☒	☐ Demonstrate how to render a vaccine ineffective
☒	☐ Confer resistance to therapeutically useful antibiotics or antiviral agents
☒	☐ Enhance the virulence of a pathogen or render a nonpathogen virulent
☒	☐ Increase transmissibility of a pathogen
☒	☐ Alter the host range of a pathogen
☒	☐ Enable evasion of diagnostic/detection modalities
☒	☐ Enable the weaponization of a biological agent or toxin
☒	☐ Any other potentially harmful combination of experiments and agents

Plants

Seed stocks	Wheat cultivars Mingxian169 (MX169), Suwon 11 (Su11) and Fielder were used in different experiments.The wheat varieties used in all three experiments were sourced from seed stocks maintained by the Plant Immunity Research Group at the College of Plant Protection, Northwest A&F University.
Novel plant genotypes	For HIGS assay, the specific cDNA segments of PstCCC1.1, PstCCC1.2 and PstCCC1.3 that were predicted using the siRNA finder software Si-Fi were inserted into the BSMV-γ carriers with NotI and PacI restriction sites. The Su11 inoculated with BSMV:TaPDS (phytoene desaturase) were used as the positive control, whereas the BSMV-γ-inoculated plants were acted as the negative control. The second leaves of Su11 were inoculated with BSMV and incubated in a plant growth chamber at 25-27°C. The wheat cultivar FiThe confirmation of positive plants in HIGS experiments was achieved by calculating the silencing efficiency of candidate genes post-inoculation, while transgenic plants carrying candidate genes were verified by detecting the inserted small RNA fragment vector using specific detection primers.elder was used to generate transgenic plants. To obtain RNAi-silenced transgenic wheat plants, the specific cDNA segments of PstCCC1.1, PstCCC1.2 and PstCCC1.3 from sensitive Pst isolate Gui1-2 were inserted into the PC336 plasmids using the gateway cloning method. For transformation, wheat calli were co-cultivated with Agrobacterium and selected on hygromycin media. For transgenic lines, two independent lines were analyzed.
Authentication	*Describe any authentication procedures for each seed stock used or novel genotype generated. Describe any experiments used to assess the effect of a mutation and, where applicable, how potential secondary effects (e.g. second site T-DNA insertions, mosiacism, off-target gene editing) were examined.*

Flow Cytometry

Plots

Confirm that:

☒ The axis labels state the marker and fluorochrome used (e.g. CD4-FITC).

☒ The axis scales are clearly visible. Include numbers along axes only for bottom left plot of group (a 'group' is an analysis of identical markers).

☐ All plots are contour plots with outliers or pseudocolor plots.

☒ A numerical value for number of cells or percentage (with statistics) is provided.

Methodology

Sample preparation	Yeast cells were placed in 96-well plates treated with different reagents. After 10 h, yeast cells were incubated with 10 μmol DCFH-DA (Sigma, MO, USA) fluorescence probe diluted in PBS at 37°C for 30 min.
Instrument	flow cytometer (Beckman, Brea, CA, USA).
Software	FlowJo software (FlowJo LLC, Delaware, USA).
Cell population abundance	Sorted yeast transformants showed ≥85% purity (re-analyzed by original gating). Key populations:ROS-high cells (DCFH-DA$^+$): 88.5±1.8% (triadimefon) vs 12.3±0.9% (control);Lipid peroxidationhigh (C11-BODIPYFL1/FL2>2.0): 85.2±2.6% (Fe^{2+} +triadimefon). Validation:Excluded: doublets (FSC-H/FSC-W) & PI$^+$ dead cells (<5%); Calibrated with CS&T beads (<2% CV);Data from ≥3 replicates (n=5,000 events).
Gating strategy	1.Initial gating: Live yeast cells were selected by FSC-A/SSC-A to exclude debris (FSC<10^3, SSC<10^2) and doublets (FSC-H vs FSC-W, diagonal gate). 2.Viability gate: PI-negative cells (<5% PI+) were gated using FL3 channel (Ex/Em 535/617 nm). 3.ROS detection (DCFH-DA):Positive population: FL1-H >10^2 (defined by unstained control, 99% in Q1);Triadimefon-treated: 88.5% FL1+ (vs 12.3% control). 4.Lipid peroxidation (C11-BODIPY):Oxidized (FL1)/reduced (FL2) ratio >2.0 = positive; Boundaries set using Fe^{2+}-treated controls (85.2% positive).

☒ Tick this box to confirm that a figure exemplifying the gating strategy is provided in the Supplementary Information.
