# Supplementary material for: Vacuolar iron transporters mediate resistance to triadimefon in plant pathogenic fungi
Source: Nat Commun. 2026 May 6;17:6379. doi: 10.1038/s41467-026-72157-6 (PMC13376413; doi:10.1038/s41467-026-72157-6)
Supplement: Supplementary file 5 — Transparent Peer Review file [file 41467_2026_72157_MOESM5_ESM.pdf]

nature portfolio

Peer Review File

Vacuolar iron transporters mediate resistance to triadimefon in plant pathogenic fungi

Corresponding Author: Professor Gangming Zhan

This file contains all reviewer reports in order by version, followed by all author rebuttals in order by version.

Version 0:

Reviewer comments:

Reviewer #1

(Remarks to the Author)
Resistance to the triazole antifungal triadimefon in the wheat pathogenic fungal species Puccinia striiformis f. sp. tritici (Pst) is becoming increasingly problematic. In general, the major triazole resistance mechanism is mutation and/or upregulation of the triazole drug, Cyp51. In previous studies the authors identified Pst isolates with increased triadimefon resistance and termed them ”low resistant” compared to ”sensitive strains”. In the current study, the authors used genetic analysis of crosses between a sensitive and a low-resistant Pst isolate to identify mutations in three CCC1 paralogs, which are thought to encode vacuolar iron importers, in the low-resistant strain. Subsequent studies in Pst, baker's yeast and Fusarium graminearum indicated that the CCC1 paralogues mediate low triadimefon resistance through vacuolar iron detoxification. The data show that triadimefon treatment induces the upregulation of CCC1 paralogous and antioxidative genes in vivo. The identified mutations appear to increase CCC1´s iron transport capacity. Overall, the data suggest that cytosolic iron amplifies oxidative stress, thereby decreasing fungal viability and triadimefon resistance, which is counteracted by vacuolar iron deposition.
Previous studies have indicated that triazoles cause oxidative stress (e.g., PMID: 25445311, PMID: 30126960) but a resistance mechanism involving vacuolar iron detoxification is novel.
It is well-known that plants combat fungal invaders by producing reactive oxygen species (ROS) locally. Furthermore, plant pathogens are challenged by host-induced iron limitation and iron excess. Poaceae plants, including wheat, locally increase iron levels to trigger a toxic oxidative burst to combat pathogens, including Fusarium graminearum (PMID: 37650633). These neglected data are important as iron detoxification appears to be particularly important during periods of high iron levels. Why is CCC1-mediated iron detoxification important: is it due to plant-derived ROS, plant-derived iron or triadimefon-mediated derangement of cellular REDOX and iron homeostasis. Does one factor come before the other or is it a combination?
Interesting questions coming from the results: (i) are Ccc1-mediated and Cyp51-mediated resistances additive? (ii) Does the mutation of CCC1 only confer resistance to triadimefon, or also to other triazol drugs as well. This should be at least tested in i the heterologous F. graminearum system (see Supplementary Figures 9 and 11 and Figure 7).
The results are interesting, but there are several shortcomings. This manuscript is difficult to read, particularly for non-experts due to poor descriptions, but even experts may struggle due to poor description. There are methodological shortcomings, with important technical details and literature missing (please see in specific comments).

____
Specific comments

1) Line 49:
Here and throughout the text: please define and stick to one term such as ”low resistant” to avoid confusion (do not mix with tolerant or resistant). I also suggest to always use the term ”sensitive” and ”not wild-type (WT)”. WT is usually used when comparing mutant strains that are otherwise isogenic.

2) Line 50:
Transcriptional upregulation of Cyp51 is highly depending on the time of triazole confrontation and might be strain-dependent. Is the untreated Cyp51 transcript level unchanged?

3) Line 100/ Figure 1:

Which Pst strain was used for transcriptional analysis, the sensitive or the low resistant one?
The rationale for performing transcriptional profiling to identify resistance-conferring gene after the genetic analysis unclear to clear to me (If there are mutations in the coding regions, there is no compelling need for a change in transcript level. Furthermore, genes that do not respond at the transcriptional level to drug confrontation may be involved in drug resistance).
Fig. 1c: The time points and the labelling of the expression columns are not explained.
Fig. 1f: how was this analysis done? What are the ”significant transcription factors involved in regulating genes involved in metal ion transport”? Very few genes are functionally characterized in Pst, I assume this is based on bakers yeast homology? Iron regulation is quite different in bakers yeast compared to filamentous ascomycetes such as Aspergillus fumigatus (PMID: 33045305) and I assume it is also different to basidiomycetes.

4) Line 152:
”…sense mutations were…”

5) Line 152/Supplementary Figure 4:
In which public database can the analyzed Pst CCC1-homologs (e.g. PstAZ2B06G00574) be found ? Otherwise please deposit them- the sequences must be publicly available. I could not find these gene ID´s in NCBI.
In Fig. S4a, the lines for the mutated and non-mutated sequences should be aligned for clarity.
The colors of Fig. S4c are not explained.
Please enlarge Figs. S4b S4c for clarity.
Can CCC1.2 really function as a vacuolar transporter -its sequence is significantly shorter than the other´s. Is the gene annotation (intron/exon prediction, etc) correct? Please check the cDNA of the analyzed homologs, which should be possible using the transcriptomic data and the cloned cDNAs. The secondary and tertiary structures of the three paralogs should be compared to those of functionally characterized homologs such as S. cerevisiae Ccc1 and A. fumigatus CccA (PMID: 23151814) using transmembrane domain predictor PROTTER (http://wlab.ethz.ch/protter/#) and AlphaFold. At least the transmembrane domains should be conserved.
How many CCC1 homologs does Pst possess? According to Fig. S4 there is at least a fourth one! Does it also contain mutations/? Is the regulation and transcript level similar to the others? S. cerevisiae possesses only a single homolog, while A. fumigatus (and numerous other fungal species) possesses two paralogs of which one (CccA) shows high upregulation in response to iron and plays an important role in iron resistance, while the other one (CccB) shows a low transcript level and is functionally uncharacterized (PMID: 23151814). CccA and CccB homologs of different fungal species cluster phylogenetically in different clades (PMID: 23151814). According to Fig. S4b, PstCCC1.1, PstCCC1.2, and PstCCC1.3 are in the same phylogenetic clade, while the fourth paralog is in a different one.
Fusarium graminearum also has two CccA homologs - which one was deleted in the current study?
Please comment on the genomic architecture of PstCCC1.1, PstCCC1.2, and Pst CCC1.3; the gene annotation numbers imply that they are neighbor genes. Which other genes are in this region? The fourth homolog appears to have a distant location? These data might indicate a local amplification of CCC1 homologous genes. What might be the reason? Resistance against triadimefon? Resistance against iron stress? Poaceae plants, including wheat, can accumulate iron locally to suppress pathogen infection (PMID: 37650633).
Please comment on the basal transcript level of the four paralogs.

6) Supplementary Figure 5 and respective text:
The upregulation of CCC1 between the 6h and 12h time point is puzzling. What is the reason for the set up used (triadimefon treatment 3 days post inoculation for 3 days + 6/12/24/48/72/120h)? Does the response reflect the triadimefon response (3.5 days?) or the plant-derived iron excess response? How long does germination require? What about the fourth paralog?
Are PstCCC1.1, PstCCC1.2, PstCCC1.3 and the fourth paralog also upregulated in the sensitive isolate (Gui1-2)?
Does the upregulation of the CCC1 homologs reflect an effect specific for these genes, or does this reflect an ”excess iron response”, i.e., is there a concomitant down-regulation of iron acquisition genes? Most filamentous fungi utilize reductive iron assimilation and siderophore-mediated iron acquisition (PMID: 33045305). For example , Pst possesses homologs of A. fumigatus genes involved in siderophore biosynthesis (SidA: KAI9614796.1, KAI9624531.1), siderophore uptake (KAI9623704, KAI9631105) and the iron permease involved in reductive iron assimilation (FtrA: KAI9609975). Compared to CccA, these genes exhibit inverse transcriptional regulation in response to iron exposure, i.e they are downregulation.
Supplementary Fig. S6 and Fig. 2 indicate that silencing of each of the three CCC1 paralogs decreases triadimefon resistance, which is not expected as there are three paralogs for the same ”job”. An explanation would be that the silencing construct silences all three paralogs. Therefore, pleas analyze the transcript levels of all three paralogs in each silenced strain.

7) Line 170 and general:
In general, please define abbreviations when they are first used at their first use, e.g., HIGS (”host-induced gene silencing) and provide a reference.
Lines 170-171 are confusing: without reading the Material and Methods´ section, it is not clear that this is a control experiment. Please explain the methodology used in more detail: HIGS and links to RNAi and BSMV (Barley stripe mosaic virus) – this is not clear to a general audience.
Please also explain PDS and lycopene dehydrogenase (in Material and Methods it is termed ”phytoene dehydrogenase”, which is confusing), photobleaching and links to gene silencing. This is also not explained in Fig. S6a.

8) Supplementary Figure 6 and respective text.
Supplementary Fig. S5 shows that triadimefon treatment increases expression of PstCCC1.1, PstCCC1.2 and PstCCC1.3 up to 40-fold! Therefore, I wonder why BSMV-mediated HIGS has a similar effect (about 50% transcriptional downregulation)

with and without triadimefon treatment? Does BSMV infection affect expression of the CCC1 homologs?
Fig. S6e shows the iron content or cytosolic iron content (I assume cytosolic)? Please specify. What was the total iron content? It is important to know whether triadimefon treatment increases the total cellular iron content or changes the cellular iron compartmentalization.

9) Supplementary Figure 2 and respective text.
What is ”Fielder”?
As for Supplementary Figure 5, it is puzzling that BSMV-mediated HIGS has the same effect with and without triadimefon treatment given that triadimefon treatment significantly increases the expression of the analyzed genes (Supplementary Fig. S5)?
Does Fig. 2d show the iron content or cytosolic iron content (I assume cytosolic)? Please specify. It is important to know whether triadimefon treatment increases the total cellular iron content or changes the cellular iron compartmentalization. It would be interesting to analyze the iron content with iron and BPS supplementation (as done in Fig. 3d), respectively!

10) Iron determination:
Was this done using urediniospores for Pst or is it possible to purify the hyphal Pst cells from plants? If urediniospores were used, the measurements may not reflect the iron content of hyphal cells. This should be stated. What was the total cellular iron content in all these measurements?

11) Line 226:
According to the ”Introduction” section, the Y134F mutation does not confer triadimefon resistance?

12) Figure 3.
While the data are interesting, they are of limited value as the strains are not isogenic apart from mutations in Cyp51 and CCC1 homologs.
Fig. 3c: To which value were the data normalized?
Fig. 3d: The sensitive strain should not be called WT since it is not isogenic with the other strains. What was measured in ”culture solution”?

13) Line 274/Supplementary Figure 7:
Technical details of the cloning strategy are missing (e.g., primers used, promoter used, etc). Was the GFP-tag N-terminal or C-terminal?
Fig. 7b/c, yeast strains were grown in SD-U, or? Please specify.
Do the different mutant strains show differences in resistance to triadimefon without 20 mM iron supplementation? Together with the shown data, this experiment may indicate if external iron stress is required to for the CCC1-mediated triadimefon resistance.

14) Line 297:
This is also true for the PstCCC1.1 with and without mutation.

15) Supplementary Figure 8/Line 334 and following:
Ferroptosis is a form of regulated cell death characterized by iron-dependent accumulation of lipid peroxidation products. In my oppinion, the data presented indicate oxidative stress (including lipid oxidation) amplified by increased cytosolic iron accumulation. I would advise against using the term 'ferroptosis', as it has never been proven in fungi, also not in this study.
What is the source or definition of ”ferroptosis”-genes shown in Supplementary Figure 8 - there is no reference.
The labelling (e.g., T5/CK5) of the gene expression columns is hardly visible and not explained.
The columns order should follow the time points, i.e., T1, T2, T3, T4, T5
The gene-IDs of the presented genes have to be supplied in Supplementary data!
It is well known that azole treatment is linked to oxidative stress (e.g., PMID: 25445311, PMID: 30126960), which is not mentioned. Therefore, authors should focus on the analysis of genes that have characterized antioxidative roles in fungi, e.g. catalases, peroxidases, superoxide dismutases, lipid peroxidation, Yap1, etc. Moreover, the expression pattern of genes involved in iron acquisition and detoxification (Ccc1) should be shown.

16) Supplementary Figure 9, Supplementary Figure 11:
To prove functionality of FgCCC1 in iron detoxification, resistance to iron excess without triadimefon treatment should be analyzed as done in baker´s yeast.

17) Supplementary Figure 11:
Fig. S11 a:
Important technical details for generation of the mutant strains are missing: Integration at the homologous locus or ectopically (identical locus)? Integration in single or multiple copy. Which promoter was used? Anyway, the PH-1 strain is not the right control; a strain with integration of the non-mutated gene should be used.
Fig. S11b/c: How was growth recorded – radial growth? How were the data normalized? Why do some of the mutant strain exhibit significant differences in radial growth on MM? I do not really see the quantified triadimefon inhibition of mycelial growth in Fig. S11a Convincing is only the difference between CCC1.3 not-mutated/mutated). I appreciate to see original pictures of fungal colonies but I suggest to analyze the triadimefon resistance by classical determination of the ”minimal inhibitory concentration (MIC)”. To prove functionality in iron detoxification of Pst1 CCC1 paralogs in F. graminearum, resistance to iron excess without triadimefon treatment should be analyzed as done in baker´s yeast.

18) Line 440 and following:
The methodology used for expression of the PstCCC1 homologs is not suitable for membrane proteins. Therefore, the 3D structure of the protein is most likely wrong and not suitable for further analyses.

19) Line 500:
E333 from PstCCC1.3 is conserved neither in FG nor SC.

20) Figure 7, Line509.
Important technical details for generation of the mutant strains are missing: the mutated FgCCC1 gene has been introduced into the FgCCC1-lacking mutant: how was this done? Integration at the homologous locus or ectopically (identical locus)? Integration in single or multiple copy. Which promoter was used - the original promoter? Anyway, the PH-1 strain is not the right control; a strain with integration of the non-mutated gene should be used.

21) Line 613:
"low resistant" not "resistant"

22) Line 654:
Please replace "ion" by "calcium"

23) Line 659:
Refs 34 and 36 do not report release of free iron ions by contributing to iron homeostasis imbalance.

24) Line 659:
Ref 38 does not fit to the statement and should be replaced by PMID: 23151814 and PMID: 25092765.

25) Figure 8:
According to the "Introduction" section, the Y134F mutation does not confer triadimefon resistance? What causes moderate triadimefon resistance?
Please avoid the terms Wild-type and ferroptosis.
The schemes do not differ apart from the marked mutations – the read out is always the same – this is not very intuitive.

26) Line 570:
The Discussion should be made more concise and compact.

Reviewer #2

(Remarks to the Author)
This manuscript reports on the intriguing discovery through genetic mapping of polymorphisms in the vacuolar iron transporter family CCC1 in one of the major pathogens of wheat as conferring increased tolerance to a commonly used fungicide. The fungicide used, triadimefon, is part of the demethylation inhibitor/azole class, the most widely used set of chemicals to control fungal diseases in agriculture and human health.

The authors then follow up this discovery with an extensive and substantial set of experiments towards establishing how this might be occurring, including using the model fungus Saccharomyces cerevisiae for heterologous expression, and then showing similar changes in resistance occur in an evolutionarily-diverse plant pathogen, Fusarium graminearum. Of note the discoveries and many of the analyses were made in an obligate plant pathogen, making much of the work technically challenging.

Hence, this research could be of wide appeal to those exploring mechanisms of resistance to azole fungicides or other genetic aspects in fungi that might mean they are less (or ideally more) effective in treating diseases.

One major point that stands out is that only a single example of this fungicide class was tested. The findings would be considerably stronger if this were a general feature across, as an example, other commonly used DMI fungicides in agriculture.

Medium points for consideration:

In figure 4, what is impact of triadimefon used alone?

The model to explain the mechanism is unclear - how is this working to provide resistance? The text is vague, about the well-known interaction between the DMI fungicides an iron (heme) cofactor within it. A discussion on the impacts of iron on Candida albicans, and no change in DMI resistance is covered in Van Genchten et al. 2024 FEBS J. Or more broadly, is there additional literature available to explain/support the model?

Minor points:

General: this focus is on China (e.g. first line of the abstract), but is this an issue globally? To follow on from this, in addition

to the analysis of the 34 isolates of Pst previously sequenced from China there are likely other genome sequences of Pst available beyond those examined in this research that might carry these polymorphisms.

The Introduction is an extended Abstract, so should be re-written.

What is the origin of the F. graminearum CCC1 mutant strain?

Line 468: the structures were not 'determined' but 'predicted'. The sentence could be 'We then predicted the structures and interactions of the vacuolar…' and on line 473: 'assay identified an interaction between'.

Text:
Line 80: 'levels that ranged'.
Lines 83-86: one can propose a segregation pattern, but the chi-square test only supports it.
Line 94: missing information to add.
Line 95: duplication on 'polymorphic' and the 'P' in 'SNP'.
Lines 98-99: add a space after the numbers and 'bp'.
Lines 125 and 131: clarify the meaning of 'sense mutation'.
Line 170: people will not know what this PDS assay is, so will need a phrase to introduce it.
Line 186: 'confirm'.
Lines 186-202: needs re-writing as confusing as to what was transgenic. It mixes 'isolate' (fungal) with 'line' (plant). It seems like the plant was transgenic expressing an RNAi molecule to target the fungus. Line 94 probably 'impact on disease relative to the untransformed control'.
Line 387: first mention of 'Fg' but the species is not written out.
Line 390: 'knockout of FgCCC1 and complementation in the deltaFgCCC1 mutant'.
Line 567: 'mutation' spelling.
Line 574: 'its' for 'their'.
Line 614: 'aggregata' spelling.
Line 739: add italics to the species.
Line 771: 'Petri' spelling.
Line 794: 'Quantitative' spelling.
Lines 807-811: unclear what 'RNA sequenc[i]es' means, i.e. RT clones?
Line 841: add something in the main text about the WGA to explain how the fungus was visualized under a fluorescence microscope.

Figures: a general comment much of the font will be too small if some aims to read this as a printed out version as well as the figures in the supplemental material.

Figures S2 is too small, and panel a the nucleotides do not align.

Reviewer #3

(Remarks to the Author)
Since fungal pathogens are rapidly evolving resistance to chemical fungicides with single-site modes of action, it is important to elucidate molecular mechanisms responsible for the resistance. In this study, the authors have identified a novel mechanism for resistance to a triazole fungicide triadimefon widely used in China for management of the stripe rust disease caused by Puccinia striiformis f. sp. tritici (Pst) in wheat. The results reported in this study are interesting because the mechanism of resistance to this triazole fungicide in specific isolates of this pathogen does not involve mutations in the Cyp51 gene as reported by a number of earlier studies. Instead, the authors have found that mutations in the genes encoding the vacuolar iron transporters are responsible for resistance to triadimefon in a few Pst isolates previously collected from the wheat fields in China. This finding is significant because it identifies a novel mechanism for fungicide resistance in an obligate biotrophic basidiomycete pathogen which is difficult to study.
Using bulked segregant analysis and genetic mapping, authors identified three candidate iron transporter genes PstCCC1.1, PstCCC1.2 and PstCCC1.3 on chromosome 6 as candidates for triadimefon resistance in Pst. Silencing these genes reduced triadimefon resistance, while sequence analysis identified specific mutations (PstCCC1.2R82C,S86P,E109K and PstCCC1.3R306C,S310P,E333K) in PstCCC1.2 and PstCCC1.3 that enhanced iron transport. Heterologous overexpression of theese candidate genes and their mutants in yeast and the ascomycete fungal pathogen Fusarium graminearum (Fg) confirmed their role in vacuolar iron transport and triadimefon sensitivity. The cytosolic iron contents in Pst isolates overexpressing these mutants were significantly lower than those in the wild-type isolates, thus providing correlation with decreased sensitivity to triadimefon. In vitro assays using microscale thermophoresis (MST) and isothermal titration calorimetry (ITC) further validated these observations, showing that specific mutations in PstCCC1.2 and PstCCC1.3 enhance iron transport into vacuole and detoxification conferring low resistance to triadimefon. Overall, the authors have used a solid mix of genetic mapping, gene expression analysis, functional experiments, and validation across multiple fungal species to provide insight into Cyp51-independent mechanism of fungicide resistance. They have provided compelling evidence for existence of this novel mechanism of fungicide resistance. Conceptually and technically, the study is sound and experiments have been conducted with rigor and correctly interpreted. However, some parts of the manuscript such as rationale behind the isolate selection, distinction between iron content and transport, and significance of ergosterol for fungicide resistance need clearer explanations.
Lines 7-11: "These mutants exhibited decreased cellular iron content and increased triadimefon tolerance." Which mutants? In the sentence before authors say heterologous expression of natural mutants in yeast and Fusarium enhances iron

transport. I think these two sentences seem confusing/contradictory on iron content and transport part. Authors may need to add more clarification if iron transport is into vacuoles, thereby reducing cytosolic iron? This part on iron transport and iron content should be clarified throughout the manuscript, it is a bit confusing.
Lines 35-41: The authors should clarify whether the single-nucleotide mutation at 401 directly translates to the Y134F substitution in Cyp51.
Lines 41-51: The authors present a rationale for investigating non-Cyp51-mediated resistance to triadimefon, citing field evidence that some Pst isolates exhibit resistance without mutations or expression changes in Cyp51. However, the authors should explain why a controlled cross was chosen over direct analysis of naturally resistant field isolates? While genetic tractability is a valid reason, this choice may limit the ecological relevance unless explicitly justified.
Why was the fungal biomass only detected after 10 days of triadimefon treatment? What happens if you go beyond that timepoint or before that?
How was the relative inhibition rate of fungicide calculated in Fig3c as well as other figures? It is not mentioned anywhere.
Lines 249–252: The statement that “exogenous ergosterol supplementation effectively reduced the cytoplasmic Fe^{2+} levels in all isolates compared to fungicide treatment alone” does not appear to be supported by the data in Figure 3d. In particular, for the moderately resistant (MR) isolate, the Fe^{2+} content under triadimefon + ergosterol treatment seems slightly higher than under triadimefon alone. This contradicts the claim made by authors. To clarify this discrepancy, I recommend the authors provide the actual mean ± SD values for Fe^{2+} content across all treatment groups. Additionally, statistical significance should be indicated to support the interpretation (for comparison of triadimefon and triadimefon+ergosterl treatments). If the differences are not statistically significant, the phrasing should be revised accordingly.
Was ergosterol supplementation tested across a concentration gradient? A dose-dependent change in Fe content might further clarify the role of dependence of moderately resistant (MR) isolate to ergosterol for fungicide resistance.
Can the authors distinguish whether ergosterol supplementation restores membrane integrity (thereby reducing iron influx) or directly modulates iron transport mechanisms? Inclusion of membrane permeability assays or expression data for iron transporters would be informative. Overall, the conclusion made on “MR isolate are more dependent on ergosterol for fungicide resistance” warrants further exploration.
Minor comments
Line 82: Change (3.18) to (3.18 μg/mL)
Line 82-83: Modify this sentence “The 82 F2 isolates had much wider range of EC50 values ranging from 0.14 to 6.93 μg/mL”.
Lines 84-86: The chi squared test ($\chi 2 = 0.27$, $P = 0.60$) segregating into a 3:1 ratio for the resistant (should be sensitive?) (EC50 <1.00) to sensitive (should be resistant?) classes (EC50 > 1.00) (Supplementary Table S3), indicating that the sensitivity of Pst to triadimefon is controlled by a single locus.
Line 94: Need to add the reference genome used “(add the genome here)”
Line 101: Please specify which three isolates were used for transcriptome analysis. What was the concentration of triadimefon used?
In all figure legends including supplementary figures, the authors should mention that YQ324 isolate is resistant and Gui1-2 isolate is sensitive add details needed in all figure legends including supplementary figures.
Figure 1b describes in detail the SNP index plot. What does each color mean? What does it show? What is the cutoff used to choose chromosome 6 and 11?
Supplementary Fig. 3c legend does not explain what the dot plot is showing. Figure does not show cluster 1 and 6 separate as described in the legend. This seems like an error. Additionally, expression trend analysis on transcriptome profiles “of jujube at” Pst at distinct temporal phases of triadimefon treatment. Jujube is not mentioned anywhere in the manuscript.
Supplementary Fig. 6a legend, inoculated “on” the fourth leaves of Sumon 11.
Line 170: The acronyms HIGS and PDS should be spelled out at their first citations in the manuscript.
Lines 173-174: Modify this sentence as “Pst inoculation and triadimefon treatment experiments were conducted on the PDS silenced plants containing the gene silencing constructs for the three candidate genes”.
Lines 174-175: Modify this sentence as “The transcription levels of PstCCC1.1, PstCCC1.2 and PstCCC1.3 decreased by 40% - 60% in the Pst-inoculated silenced plants”
Lines 176-177: “but significantly reduced resistance in the triadimefon treatment” not clear what this means. Do the authors mean to say that the plants had increased Pst virulence upon triadimefon treatment.
Lines 186-187: Modify this sentence as “To further confirm the results of the transient silencing of PstCCC genes, results, transgenic Fielder wheat variety was generated expressing RNAi directed against these genes”.
Line 189: State whether T3 plants were homozygous.
Line 198-201: Modify this sentence as “To further determine whether the RNAi transgenic lines inoculated with Pst affected the growth and development of hyphae after treatment with triadimefon, the development of hyphae was observed and measured using a fluorescence microscope”.
Line 198: “To” further determine
Fig 2b Statistics on without triadimefon treatment are missing.
Fig 3a legend indicates wild type (WT), moderately resistant (MR), and low resistant (LR) in the legend as well.
Fig. 4b and 4c should be described and cited immediately after Fig. 4a. Please modify the text accordingly.
Fig. 5c second graph add “T” on top for triadimefon treatment
Fig. 8. For the moderately resistant isolate part of the model, the authors state that “This reduces the destructive power of the pathogen cell membrane, and the level of ferroptosis caused by ferrous ion overload inside the cell is greatly reduced compared to the wild-type isolates, resulting in a higher level of triadimefon resistance”. However, the model does not show decreased ferroptosis.
Authors should provide both excitation and emission wavelength of the fluorescent dyes/channels used in the study in the figure legends including supplementary figures.
Supplementary Fig. 11a 20 mM FeSO4 or 2 mM FeSO4? In the Results section, authors have mentioned 20 mM but in figure it appears 2 mM. Also, what does MM stand for?

The manuscript will greatly benefit from thorough editorial revision to enhance clarity and to ensure consistency in scientific expression.

Reviewer #4

(Remarks to the Author)
I co-reviewed this manuscript with one of the reviewers who provided the listed reports. This is part of the Nature Communications initiative to facilitate training in peer review and to provide appropriate recognition for Early Career Researchers who co-review manuscripts.

Version 1:

Reviewer comments:

Reviewer #1

(Remarks to the Author)
The authors addressed most of my questions and suggestions satisfactorily, with exception of my comment 18.

18) Line 440 and following (original manuscript): The methodology used for expression of the PstCCC1 homologs is not suitable for membrane proteins. Therefore, the 3D structure of the protein is most likely wrong and not suitable for further analyses.

I did not question the 3D structure predictions by AlphaFold 3. Rather, I criticised the methodology used to express the PstCCC1 proteins. The authors expressed the proteins in E. coli, followed by a purification protocol intended for hydrophilic proteins, which is not suitable for membrane proteins (Wwhy was PstCCC1 expressed as an MBP fusion protein when smaller affinity tags were used for the others?) As shown by SDS-PAGE analysis (see Supplementary Figure 15), the purification procedure yielded heterogeneous fractions. Therefore, the proteins of the correct size were excised from the SDS-PAGE gels. As SDS-PAGE is a denaturing method, the 3D structures of the produced proteins are most likely incorrect and not suitable for further analysis, such as microscale thermophoresis or isothermal titration calorimetry.

Minor:
The authors largely eliminated the use of the term ferroptosis but at Line 745 the term ”ferroptotic cascade” is till used.

Reviewer #3

(Remarks to the Author)
We thank the authors for carefully addressing the issues raised in our review of this manuscript. The authors have satisfactorily responded to all our concerns and comments and made changes in the manuscript accordingly. The editorial changes have also greatly improved the flow of the manuscript making the interpretation of the data easier to understand.

Reviewer #4

(Remarks to the Author)
I co-reviewed this manuscript with one of the reviewers who provided the listed reports. This is part of the Nature Communications initiative to facilitate training in peer review and to provide appropriate recognition for Early Career Researchers who co-review manuscripts.

Version 2:

Reviewer comments:

Reviewer #1

(Remarks to the Author)
I thank the authors for addressing the issues raised in my review. The authors have satisfactorily responded to all my comments and made changes accordingly.

Open Access This Peer Review File is licensed under a Creative Commons Attribution 4.0 International License, which permits use, sharing, adaptation, distribution and reproduction in any medium or format, as long as you give appropriate credit to the original author(s) and the source, provide a link to the Creative Commons license, and indicate if changes were made.
In cases where reviewers are anonymous, credit should be given to 'Anonymous Referee' and the source.
The images or other third party material in this Peer Review File are included in the article's Creative Commons license, unless indicated otherwise in a credit line to the material. If material is not included in the article's Creative Commons license and your intended use is not permitted by statutory regulation or exceeds the permitted use, you will need to obtain permission directly from the copyright holder.
To view a copy of this license, visit https://creativecommons.org/licenses/by/4.0/

POINT-BY-POINT RESPONSE TO REVIEWERS' COMMENTS

Reviewer #1 (Remarks to the Author):

Resistance to the triazole antifungal triadimefon in the wheat pathogenic fungal species Puccinia striiformis f. sp. tritici (Pst) is becoming increasingly problematic. In general, the major triazole resistance mechanism is mutation and/or upregulation of the triazole drug, Cyp51. In previous studies the authors identified Pst isolates with increased triadimefon resistance and termed them ”low resistant” compared to ”sensitive strains”. In the current study, the authors used genetic analysis of crosses between a sensitive and a low-resistant Pst isolate to identify mutations in three CCC1 paralogs, which are thought to encode vacuolar iron importers, in the low-resistant strain. Subsequent studies in Pst, baker's yeast and Fusarium graminearum indicated that the CCC1 paralogues mediate low triadimefon resistance through vacuolar iron detoxification. The data show that triadimefon treatment induces the upregulation of CCC1 paralogous and antioxidative genes in vivo. The identified mutations appear to increase CCC1´s iron transport capacity. Overall, the data suggest that cytosolic iron amplifies oxidative stress, thereby decreasing fungal viability and triadimefon resistance, which is counteracted by vacuolar iron deposition.

Previous studies have indicated that triazoles cause oxidative stress (e.g., PMID: 25445311, PMID: 30126960) but a resistance mechanism involving vacuolar iron detoxification is novel.

It is well-known that plants combat fungal invaders by producing reactive oxygen species (ROS) locally. Furthermore, plant pathogens are challenged by host-induced iron limitation and iron excess. Poaceae plants, including wheat, locally increase iron levels to trigger a toxic oxidative burst to combat pathogens, including Fusarium graminearum (PMID: 37650633). These neglected data are important as iron detoxification appears to be particularly important during periods of high iron levels. Why is CCC1-mediated iron detoxification important: is it due to plant-derived ROS, plant-derived iron or triadimefon-mediated derangement of cellular REDOX and iron homeostasis. Does one factor come before the other or is it a combination?

Response: We thank the reviewer for this valuable question. We fully agree with your point, and this factor was incorporated into our experimental design. To minimize the impact of pathogen-triggered early ROS bursts, our triadimefon treatment was conducted at 72 hours after inoculation with *Pst*. It is well established that the ROS burst in induced by pathogen invasion occurs predominantly during the early stages of infection and largely subsides by 72 hours post-inoculation (Wang et al. 2008: DOI:10.1016/j.pmpp.2008.02.006; PMID: 34999846; PMID: 33692546).

To further evaluate this, we compared the expression patterns of reactive oxygen species (ROS)-related genes in wheat during *Pst* infection and during triadimefon treatment. In the absence of triadimefon treatment, early pathogen infection induced a clear upregulation of ROS-related genes, consistent with an early ROS burst (**Fig. R1a**). However, when triadimefon treatment was initiated at 72 hours post-inoculation with *Pst*, no significant upregulation of ROS-related genes was observed thereafter. (**Fig. R1b**). These results indicate that triadimefon treatment itself does not trigger an additional ROS burst under our experimental conditions and thus does not act synergistically with plant-derived ROS.

While these data cannot fully exclude the possibility that CCC1 contributes to pathogen survival against plant-derived ROS, our findings support the conclusion that the primary importance of CCC1-mediated iron detoxification in this context arises from triadimefon-induced disruption of cellular redox balance and iron homeostasis, rather than from a combined effect of pathogen-derived, plant-derived, or chemical-induced ROS.

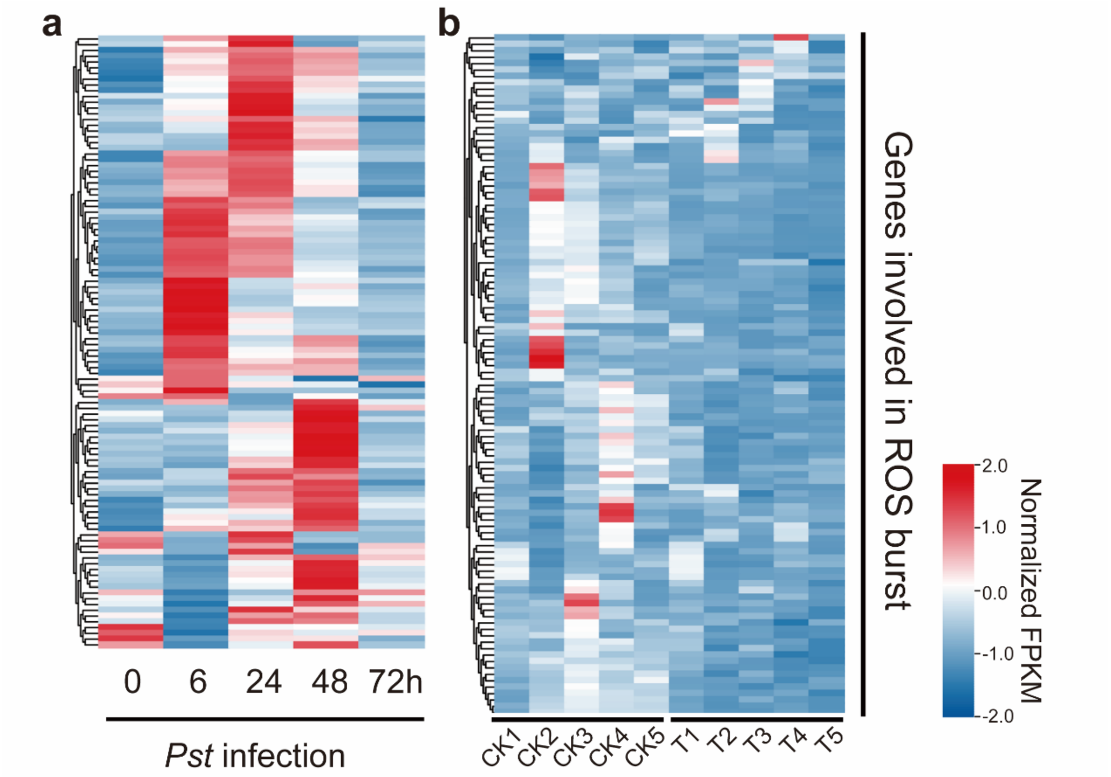

Fig. R1 Fig. R1 Expression abundances of genes involved in ROS burst in wheat following *Pst* challenge before and after triadimefon treatment. **a** Expression levels of ROS-related genes in wheat post different time points of *Pst* inoculation. The scale bar represents the normalized FPKM value about these genes. The high and low expression levels are shown in red and blue, respectively. **b** Expression levels of ROS-related genes in *Pst*-infected wheat at different time points of triadimefon treatment. Triadimefon treatments were performed at 72 hours post-inoculation of *Pst*. T1-T5: time points at 0, 24, 48, 72, and 120 hours after triadimefon treatment.CK1-CK5: corresponding untreated control samples collected at the same time points as T1-T5, respectively.

Interesting questions coming from the results: (i) are Ccc1-mediated and Cyp51-mediated resistances additive?

Response: We thank the reviewer for raising this interesting and important question regarding the potential additivity of CCC1-mediated and Cyp51-mediated resistance mechanisms. In our study, we indeed observed that among the 33 resistant *Pst* isolates analyzed, moderate resistance was associated with mutations in *Cyp51* (specifically Y134F), while low resistance was linked to mutations in *PstCCC1* genes. Notably, we did not identify any isolates carrying mutations in both *Cyp51* and *PstCCC1* simultaneously. Furthermore, no highly resistant isolates were detected in our field

collections.

We agree with the reviewer that the co-occurrence of mutations in both *Cyp51* and *PstCCC1* could potentially lead to an additive or synergistic enhancement of resistance, possibly resulting in a highly resistant phenotype. This represents a plausible evolutionary pathway under continued fungicide selection pressure.

We plan to address this hypothesis in our future work by expanding our monitoring efforts to include more diverse geographic populations and conducting genetic crosses to experimentally test the combined effect of these mutations. We appreciate the reviewer's insightful suggestion and incorporated a discussion on the potential additivity of these resistance mechanisms into the revised manuscript. (**Line 726-731**).

(ii) Does the mutation of CCC1 only confer resistance to triadimefon, or also to other triazol drugs as well. This should be at least tested in the heterologous F. graminearum system (see Supplementary Figures 9 and 11 and Figure 7).

Response: We thank the reviewer for this valuable question. To determine whether the PstCCC1 mutation confers resistance specifically to triadimefon or more broadly to other triazole fungicides, we performed additional assays. We tested sensitivity to two additional triazoles (tebuconazole and hexaconazole) and included the SDHI fungicide flubeneteram for comparison. These experiments were conducted both in the heterologous *Fusarium graminearum* expression system and in *Pst* with CCC1 silencing.

Our new data show that the mutant CCC1 alleles confer reduced sensitivity to all three triazole fungicides. In contrast, sensitivity to flubeneteram remained unchanged. These findings indicate that CCC1-mediated resistance confer resistance to multiple triazoles rather than being specific to triadimefon, but not to SDHI fungicide flubeneteram.

We have added these new experimental results, which are presented in **Fig. 2**, **Fig. 7**, and **Supplementary Fig.** 13, with corresponding descriptions in their captions.

The results are interesting, but there are several shortcomings. This manuscript is difficult to read, particularly for non-experts due to poor descriptions, but even experts may struggle due to poor description. There are methodological shortcomings, with

important technical details and literature missing (please see in specific comments).

Response: We thank the reviewer for this constructive feedback. We acknowledge the issues raised regarding clarity and completeness of methodological descriptions. In the revised manuscript, we have substantially improved the readability by refining the text, clarifying experimental descriptions, and reorganizing sections where needed to assist both expert and non-expert readers. In addition, we have carefully addressed all methodological shortcomings by including the missing technical details and adding the relevant literature cited in the specific comments.

Specific comments:

1) Line 49:

Here and throughout the text: please define and stick to one term such as ”low resistant” to avoid confusion (do not mix with tolerant or resistant). I also suggest to always use the term ”sensitive” and ”not wild-type

(WT)”. WT is usually used when comparing mutant strains that are otherwise isogenic.

Response: We sincerely thank the reviewer for pointing out the inconsistency in terminology. In the revised manuscript, we now consistently use “sensitive” for susceptible isolates and “low resistant” or “moderately resistant” for isolates with reduced sensitivity to triadimefon. We have also restricted the term “wild-type (WT)” to contexts where it is appropriate-specifically, comparisons involving isogenic mutants in heterologous systems. These revisions have been applied throughout the manuscript, figures, and tables to ensure clarity and consistency.

2) Line 50:

Transcriptional upregulation of Cyp51 is highly depending on the time of triazole confrontation and might be strain-dependent. Is the untreated Cyp51 transcript level unchanged?

Response: We thank the reviewer for this insightful comment regarding the potential dynamics of *Cyp51* expression. To determine whether basal transcript levels differ among isolates, we performed a time-course analysis of *Cyp51* expression in the sensitive (Gui1-2), low-resistant (YQ324), and moderately resistant (TS-7) isolates, with and without triadimefon treatment, at 0, 6, 12, 24, 48, 72, and 120 hours. As shown

in the new **Supplementary Fig. R2**, untreated *Cyp51* transcript levels remained stable and comparable across all isolates throughout the time course. In contrast, triadimefon treatment induced significant *Cyp51* upregulation in all three isolates, indicating that the transcriptional induction is a general response to triazole exposure. These results demonstrate that the observed differences in resistance are not due to inherent variation in basal *Cyp51* expression, but instead arise from other mechanisms.

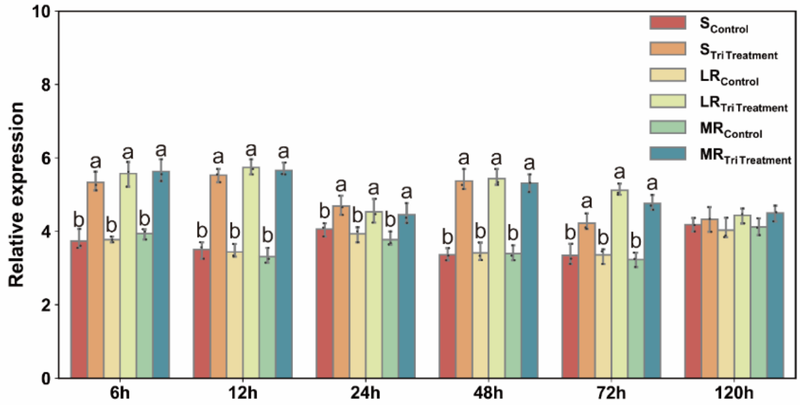

Fig. R2 Expression analysis of *Cyp51* in moderately resistant isolate (TS-7), low-resistant (YQ324), and sensitive (Gui1-2) *Pst* isolates with or without fungicide treatment. Wheat seedlings (cv. Mingxian 169) were inoculated with TS-7, YQ324 and Gui1-2 urediniospores at 10 days after planting and treated with Tri at 3 days after *Pst* inoculation. Leaf samples were then collected at 0, 6, 12, 24, 48, 72, and 120 hours after Tri treatment for RNA extraction to determine the expression levels of *Cyp51*. Expression levels were normalized based on the *Pst* elongation factor gene (*PstEF*). The relative expression values of *Cyp51* were calculated using the comparative threshold method ($2^{-\Delta\Delta Ct}$). The experiment was repeated three times independently. Different letters indicate significant differences as determined by one-way ANOVA with Duncan's new multiple range test ($P < 0.05$).

3) Line 100/ Figure 1:

Which Pst strain was used for transcriptional analysis, the sensitive or the low resistant one?

Response: Revised, we used the low-resistant *Pst* isolate in this work, and have clarified this in the revised manuscript.

The rationale for performing transcriptional profiling to identify resistance-conferring gene after the genetic analysis unclear to clear to me (If there are mutations in the coding

regions, there is no compelling need for a change in transcript level. Furthermore, genes that do not respond at the transcriptional level to drug confrontation may be involved in drug resistance).

Response: We thank the reviewer for this important comment. We agree that mutations in coding regions may confer resistance independently of transcriptional changes, and that genes not induced by fungicide exposure can still play critical roles in resistance. Our aim in performing transcriptional profiling was not to assume that resistance requires transcriptional induction, but rather to integrate expression data with genetic mapping to more confidently identify the major resistance-conferring gene(s).

By integrating transcriptomic and BSA data, we found a strong convergence on chromosome 6: both the resistance-associated mutations and the major fungicide-responsive genes mapped to this region. This convergence substantially narrows the candidate interval and provides functional context supporting CCC1 as the primary resistance-conferring gene. We have revised the text to clarify the rationale for performing transcriptional profiling to identify resistance-conferring gene (**Line 111-113**).

Fig. 1c: The time points and the labelling of the expression columns are not explained.

Response: We thank the reviewer for this comment. We have now clarified the time points and added an explanation for the expression scale in the **Fig. 1c** legend of the revised manuscript.

Fig. 1f: how was this analysis done? What are the ”significant transcription factors involved in regulating genes involved in metal ion transport”? Very few genes are functionally characterized in Pst, I assume this is based on baker yeast homology? Iron regulation is quite different in bakers yeast compared to filamentous ascomycetes such as Aspergillus fumigatus (PMID: 33045305) and I assume it is also different to basidiomycetes.

Response: The gene co-expression network in **Fig. 1f** was constructed using the R package WGCNA based on FPKM values of genes related to metal ion transport and PstCCC1.1-PstCCC1.3, with connection strengths representing Pearson’s correlation coefficients and corresponding *P*-values.

The sentence "significant transcription factors involved in regulating genes involved in metal ion transport" has been revised to a more accurate description: "The weighted co-expression network illustrating the close relationships between *PstCCC1.1-PstCCC1.3* and genes relevant to metal ion transport during fungicide treatment. *PstCCC1.1-PstCCC1.3* and all differentially expressed genes were included in constructing the co-expression network. Genes associated with cell death, iron homeostasis, metal ion transport, and primary metabolism are highlighted in blue, orange, purple, and green, respectively."

As you correctly noted, functional annotation based solely on Saccharomyces cerevisiae homologs is insufficient, especially given well-known differences in iron regulation between yeasts, filamentous ascomycetes (e.g., *Aspergillus fumigatus*, PMID: 33045305), and basidiomycetes such as *Pst*. To improve annotation accuracy, we used an integrated pipeline including: NR (non-redundant protein) database, GO annotation, KEGG annotation, and conserved domain analysis. Conserved domain analysis was particularly important in supporting putative functions independent of species-specific regulatory differences. Detailed annotations for all chromosome 6 genes used in this analysis are now provided in **Supplementary Table S5**.

4) Line 152:

"…sense mutations were…"

Response: Thank you for your correction, we have corrected the phrase 'sense mutation, to the "... missense mutations were..." as you suggested (Now in **Line 136**).

5) Line 152/Supplementary Figure 4:

In which public database can the analyzed Pst CCC1-homologs (e.g. PstAZ2B06G00574) be found ? Otherwise please deposit them- the sequences must be publicly available. I could not find these gene ID´s in NCBI.

Response: Revised as you suggested, the complete genome sequence and detailed gene annotations, including the CCC1 homologs (e.g., *PstAZ2B06G00574*), have now been deposited and are publicly accessible in the Pst AZ2 reference genome at NCBI: ASM3951922v1 (https://www.ncbi.nlm.nih.gov/datasets/genome/GCA_039519225.1/).

In Fig. S4a, the lines for the mutated and non-mutated sequences should be aligned for clarity.

The colors of Fig. S4c are not explained. Please enlarge Figs. S4b S4c for clarity.

Response: We thank the reviewer for this helpful suggestion. In response, we have aligned the mutated and non-mutated sequences in **Fig. S4a** to improve clarity. For **Fig. S4c**, we have added a detailed explanation of the color scheme in the figure legend to describe the sequence similarity levels. These changes have been incorporated into the revised manuscript.

Please enlarge Figs. S4b S4c for clarity.

Response: Revised as suggested.

Can CCC1.2 really function as a vacuolar transporter -its sequence is significantly shorter than the other´s. Is the gene annotation (intron/exon prediction, etc) correct? Please check the cDNA of the analyzed homologs, which should be possible using the transcriptomic data and the cloned cDNAs.

Response: We thank the reviewer for raising this important point. Analysis of both cDNA and genomic sequences showed that *PstCCC1.2* possesses an independent promoter and a complete coding sequence, supporting its capacity for autonomous transcription and translation. Our genetic and functional analyses further support the role of *PstCCC1.2* as an iron transporter: silencing of *PstCCC1.2* led to iron accumulation in the cytoplasm of *Pst* following fungicide treatment (See **Fig. 2**). Detailed sequence information for *PstCCC1.1* (*PstAZ2B06G00574*) and *PstCCC1.2* (*PstAZ2B06G00575*) can be found in the Pst AZ2 reference genome (NCBI: ASM3951922v1, https://www.ncbi.nlm.nih.gov/datasets/genome/GCA_039519225.1/).

The secondary and tertiary structures of the three paralogs should be compared to those of functionally characterized homologs such as S. cerevisiae Ccc1 and A. fumigatus CccA (PMID: 23151814) using transmembrane domain predictor PROTTER (http://wlab.ethz.ch/protter/#) and AlphaFold. At least the transmembrane domains should be conserved.

Response: We thank the reviewer for this suggestion. Following your recommendation,

we performed comparative analyses of the secondary and tertiary structures of PstCCC1.1-PstCCC1.3 relative to functionally characterized homologs, *S. cerevisiae* Ccc1 (ScCcc1) and *A. fumigatus* CccA (AfCccA), using PROTTER for transmembrane domain prediction and AlphaFold for structural modeling (see new **Supplementary Fig. 19**).

Our results indicate that genetic variations induce conformational changes in all three paralogs. PstCCC1.1 adopts a conformation most similar to ScCcc1 and AfCccA, whereas PstCCC1.2 and PstCCC1.3 show greater divergence. Analysis of transmembrane domains revealed notable differences: ScCcc1 and AfCccA each have three transmembrane helices, while PstCCC1.1 lacks transmembrane domains, PstCCC1.2 has three, and PstCCC1.3 has four. After mutation, PstCCC1.1^{Y300H} remains without transmembrane domains, retaining weak activity; PstCCC1.2^{R82C,S86P,E109K} maintains its three domains but gains an extended extracellular segment, correlating with enhanced activity; PstCCC1.3^{R306C,S310P,E333} shows reduced transmembrane domains but a significantly longer extracellular region, likely enabling more efficient iron capture and the greatest functional change.

Furthermore, the protein-protein interaction analysis suggests that PstCCC1.1 can interact with PstCCC1.2 to form a functional heteromeric CCC1 complex (**Supplementary Fig. 17**). Using FoldX, we showed that the mutations strengthen the interaction between PstCCC1.1^{Y300H} and PstCCC1.2^{R82C,S86P,E109K}, supporting enhanced complex functionality. These structural and interaction data collectively explain how sequence variations contribute to altered iron transport activity among the PstCCC1 paralogs.

How many CCC1 homologs does Pst possess? According to Fig. S4 there is at least a fourth one! Does it also contain mutations/? Is the regulation and transcript level similar to the others? S. cerevisiae possesses only a single homolog, while A. fumigatus (and numerous other fungal species) possesses two paralogs of which one (CccA) shows high upregulation in response to iron and plays an important role in iron resistance, while the other one (CccB) shows a low transcript level and is functionally uncharacterized (PMID: 23151814). CccA and CccB homologs of different fungal

species cluster phylogenetically in different clades (PMID: 23151814). According to Fig. S4b, PstCCC1.1, PstCCC1.2, and PstCCC1.3 are in the same phylogenetic clade, while the fourth paralog is in a different one.

Response: We thank the reviewer for this insightful comment. As noted, *Pst* possesses four homologs of PstCCC1. Our analysis revealed that PstCCC1.4 contains no mutations and is expressed at very low levels in *Pst*, even under fungicide treatment.

To address the phylogenetic context, we performed a comprehensive analysis including CccA and CccB homologs from *Aspergillus fumigatus* and *Penicillium rubens* (PMID: 23151814). The resulting tree (**Fig. S4b**) shows that *PstCCC1.1*–*PstCCC1.3* form a distinct clade, separate from the CccA/CccB cluster, whereas *PstCCC1.4* resides on a separate branch.

Given its divergent phylogenetic position and consistently negligible transcript levels, we assumed that *PstCCC1.4* does not contribute significantly to the core CCC1-mediated functions under investigation. Therefore, our study focused on the evolutionarily conserved and expressed paralogs *PstCCC1.1-PstCCC1.3*.

Fusarium graminearum also has two CccA homologs - which one was deleted in the current study?

Response: In this study, the *FGSG_07832* (XP_011327657.1) was deleted in *Fusarium graminearum*. This homolog was selected based on phylogenetic analysis (**Fig. S4b**), which shows that *FGSG_07832* clusters with the major functional *Pst* homologs (*PstCCC1.1*–*PstCCC1.3*), suggesting shared functional roles. The other homolog, *FGSG_07679*, occupies a separate phylogenetic branch and was not targeted.

Please comment on the genomic architecture of PstCCC1.1, PstCCC1.2, and Pst CCC1.3; the gene annotation numbers imply that they are neighbor genes. Which other genes are in this region? The fourth homolog appears to have a distant location? These data might indicate a local amplification of CCC1 homologous genes. What might be the reason? Resistance against triadimefon? Resistance against iron stress? Poaceae plants, including wheat, can accumulate iron locally to suppress pathogen infection (PMID: 37650633).

Response: We thank the reviewer for these insightful questions regarding the genomic architecture of the *PstCCC1* genes. As noted, *PstCCC1.1-PstCCC1.3* are neighboring genes located within a defined QTL region on chromosome 6 (**Fig. 1b**), whereas the fourth homolog (*PstCCC1.4*) is situated in a distant genomic location, consistent with a local gene amplification event.

As your suggested, we further identified the genes near *PstCCC1.1-PstCCC1.3* within this locus and investigated their functions using series gene annotation database. The results showed that these genes mainly performed functions in iron transport, particularly "ferrous iron transmembrane transporter activity" (**Fig. 1d; Supplementary Table S5**). Moreover, transcriptome results showed that most of these genes were also activated to upregulate in *Pst* by triadimefon treatment(**Fig. 1c**). This gave us a hint that that *PstCCC1.1-PstCCC1.3* and their neighboring genes might form a specific gene cluster that regulated the iron homeostasis in *Pst*, and the regulators that initiated this gene cluster might be an important target for fungicide.

Regarding the evolutionary driver of this local amplification, we agree that resistance to iron stress is the most plausible explanation. While triadimefon resistance is a recent selective pressure, the establishment of this CCC1 cluster likely predates fungicide use. In the discussion (**Line 658-665**), now we propose that the amplification of vacuolar iron transporters evolved as an adaptive mechanism to mitigate iron toxicity, potentially in response to iron-based defense strategies of Poaceae plants, which can locally accumulate iron to suppress pathogen infection (PMID: 37650633). The pre-existence of this iron detoxification system may have subsequently facilitated the evolution of triadimefon resistance in *Pst*.

Please comment on the basal transcript level of the four paralogs.

Response: Revised as you suggested, In the revised manuscript, we have provided the basal transcript level of these four *CCC1* paralogs in **Supplementary Fig. 5a**. The results showed that all *PstCCC1.1*, *PstCCC1.2* and *PstCCC1.3* were upregulated in *Pst* in response to fungicide treatment with *PstCCC1.3* exhibiting the highest basal expression, consistent with its prominent role in conferring triadimefon resistance in *Pst*. In contrast, *PstCCC1.4* had the lowest basal transcript level and showed no

induction upon fungicide treatment. These data support our conclusion that *PstCCC1.4* does not contribute to fungicide resistance, and thus its function was not further investigated in this study.

6) Supplementary Figure 5 and respective text:

The upregulation of CCC1 between the 6h and 12h time point is puzzling. What is the reason for the set up used (triadimefon treatment 3 days post inoculation for 3 days + 6/12/24/48/72/120h)? Does the response reflect the triadimefon response (3.5 days?) or the plant-derived iron excess response? How long does germination require? What about the fourth paralog?

Response: Thank you for your thoughtful question. The sampling time points in **Supplementary Fig. S5** (0, 6, 12, 24, 48, 72, and 120 h) refer to hours after triadimefon treatment, which was applied at 3 days post-inoculation. We have revised the figure legend and corresponding text to make them clear. By this time, *Pst* germination and penetration have typically completed (within ~24 h post-inoculation), indicating that the observed expression changes reflect a response to triadimefon rather than developmental processes or plant-derived iron signals.

We also analyzed the fourth paralog, *PstCCC1.4*, in both sensitive and low-resistant isolates with and without triadimefon treatment. As shown in **Fig. R3**, *PstCCC1.4* exhibits very low basal expression and shows no induction upon fungicide treatment. These results strongly suggest that *PstCCC1.4* does not contribute to triadimefon resistance.

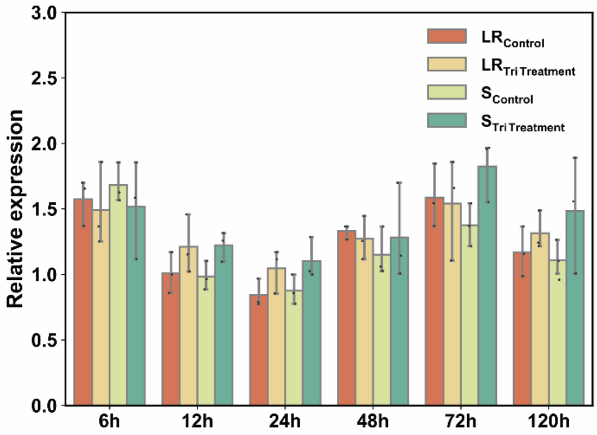

Fig. R3 Expression analysis of *PstCCC1.4* in resistant (YQ324) and sensitive (Gui1-2) *Pst* isolates with or without fungicide treatment. Wheat seedlings (cv. Mingxian 169)

were inoculated with YQ324 and Gui1-2 urediniospores at 10 days after planting and treated with Tri at 3 days after *Pst* inoculation. Leaf samples were then collected at 0, 6, 12, 24, 48, 72, and 120 hours after Tri treatment for RNA extraction to determine the expression levels of *PstCCC1.4*. Expression levels were normalized based on the *Pst* elongation factor gene (*PstEF*). The relative expression values of *PstCCC1.4* were calculated using the comparative threshold method ($2^{-\Delta\Delta Ct}$). The experiment was repeated three times independently. Different letters indicate significant differences as determined by one-way ANOVA with Duncan's new multiple range test ($P < 0.05$).

Are PstCCC1.1, PstCCC1.2, PstCCC1.3 and the fourth paralog also upregulated in the sensitive isolate (Gui1-2)?

Response: We thank the reviewer for raising this important question. We have performed additional experiments to analyze the expression of all four paralogs in both the sensitive (Gui1-2) and resistant (YQ324) isolates. The new data, included in the revised **Supplementary Figure 5b-5d**, show that *PstCCC1.1*, *PstCCC1.2*, and *PstCCC1.3* were indeed upregulated by triadimefon treatment in both isolates, whereas *PstCCC1.4* did not show any significant induction in either of the background.

Because *PstCCC1.4* is neither within the identified QTL nor contains resistance-associated mutations, it was excluded from the revised figure. These data indicate that triadimefon-induced expression of *PstCCC1.1–PstCCC1.3* represents a general stress response, while the specific amino acid changes in these genes are the key determinants of resistance.

Does the upregulation of the CCC1 homologs reflect an effect specific for these genes, or does this reflect an "excess iron response", i.e., is there a concomitant down-regulation of iron acquisition genes? Most filamentous fungi utilize reductive iron assimilation and siderophore-mediated iron acquisition (PMID: 33045305). For example , Pst possesses homologs of A. fumigatus genes involved in siderophore biosynthesis (SidA: KAI9614796.1, KAI9624531.1), siderophore uptake (KAI9623704, KAI9631105) and the iron permease involved in reductive iron assimilation (FtrA: KAI9609975). Compared to CccA, these genes exhibit inverse transcriptional regulation in response to iron exposure, i.e they are downregulation.

Response: We thank the reviewer for this insightful comment. In response, we have analyzed the expression of *Pst* homologs involved in siderophore biosynthesis (SidA: KAI9614796.1, KAI9624531.1), siderophore uptake (KAI9623704, KAI9631105), and reductive iron assimilation (FtrA: KAI9609975) under triadimefon treatment. The results, provided in **Supplementary Fig. S8**, show that most genes involved in iron acquisition exhibited reduced expression following fungicide exposure, while others remain unchanged.

These findings indicate that the upregulation of *PstCCC1* homologs reflects an “excess iron response,” consistent with the downregulation of iron acquisition pathways observed in other filamentous fungi under high-iron conditions (PMID: 33045305), rather than a gene-specific effect of triadimefon.

Supplementary Fig. S6 and Fig. 2 indicate that silencing of each of the three CCC1 paralogs decreases triadimefon resistance, which is not expected as there are three paralogs for the same ”job”. An explanation would be that the silencing construct silences all three paralogs. Therefore, pleas analyze the transcript levels of all three paralogs in each silenced strain.

Response: We thank the reviewer for this insightful comment. To address the possibility of cross-silencing, we performed additional RT-PCR analyses to measure the transcript levels of all three *PstCCC1* paralogs in each silenced strain. The results, shown in **Supplementary Fig. 7,** confirm that each silencing construct specifically reduced the expression of its target gene without affecting the other two paralogs

This specificity explains the phenotypic differences observed in **Fig. 2** and **Supplementary Fig. 6**. For example, silencing *PstCCC1.1* caused a weaker reduction in triadimefon resistance compared to silencing *PstCCC1.2* or *PstCCC1.3*, as the latter two remained functional and maintained substantial iron transport capacity. These results indicate that the three paralogs have non-redundant roles, with *PstCCC1.2* and *PstCCC1.3* contributing more strongly to resistance. We have incorporated this explanation into the revised manuscript (**Line 199-201**; **Line 216**).

7) Line 170 and general:

In general, please define abbreviations when they are first used at their first use, e.g.,

HIGS (”host-induced gene silencing) and provide a reference.

Response: We thank the reviewer for this important reminder regarding the proper definition of abbreviations. We apologize for the oversight and have carefully reviewed the entire manuscript to ensure that all abbreviations, including HIGS (host-induced gene silencing), are defined at their first occurrence in both the main text and figure legends. Appropriate references have been added for each methodological abbreviation where relevant.

Lines 170-171 are confusing: without reading the Material and Methods´ section, it is not clear that this is a control experiment. Please explain the methodology used in more detail: HIGS and links to RNAi and BSMV (Barley stripe mosaic virus) – this is not clear to a general audience.

Response: We thank the reviewer for pointing out the lack of clarity regarding the methodology in lines 170-171. We have revised the text to explicitly indicate that the experiment using PDS (phytoene desaturase) served as a positive control for the HIGS system. Additionally, we have briefly explained the HIGS technique and its connection to RNAi and BSMV (Barley stripe mosaic virus). The revised text now reads: “*After 10 days of inoculation with PDS (phytoene desaturase, a positive control for silencing efficiency), obvious photobleaching symptoms were observed in wheat leaves, indicating that the HIGS system mediated by Barley stripe mosaic virus (BSMV) was effective.*”

We also added clarification in the Methods section: “*HIGS is a technique that utilizes viral vectors (BSMV) to deliver RNAi constructs into host plants, leading to the silencing of target genes in the Pst* ”.

These revisions make the methodology accessible to a broader audience without requiring readers to consult the Methods section.

Please also explain PDS and lycopene dehydrogenase (in Material and Methods it is termed ”phytoene dehydrogenase”, which is confusing), photobleaching and links to gene silencing. This is also not explained in Fig. S6a.

Response: We sincerely thank the reviewer for this correction. The term “lycopene dehydrogenase” was wrongly used and now it is corrected to “phytoene

dehydrogenase”.

To clarify for a general audience, we have expanded the explanation of the HIGS methodology. PDS is a key enzyme in carotenoid biosynthesis. Silencing PDS via BSMV-mediated HIGS inhibits carotenoid production, which normally protects chlorophyll from photo-oxidation. Without carotenoids, chlorophyll is degraded under light, causing the characteristic photobleaching (white leaf) phenotype. This visible effect is non-lethal and serves as a reliable positive control, confirming that viral delivery, RNAi processing, and gene silencing are functioning effectively.

In HIGS experiments, plants inoculated with BSMV vectors carrying a fragment of the wheat TaPDS gene should show photobleaching, indicating a successful performance of HIGS experiment. We have updated the text and the legend of **Supplementary Fig. S6a** to include this explanation, linking the photobleaching phenotype directly to gene silencing and validation of the HIGS system.

8) Supplementary Figure 6 and respective text.

Supplementary Fig. S5 shows that triadimefon treatment increases expression of PstCCC1.1, PstCCC1.2 and PstCCC1.3 up to 40-fold! Therefore, I wonder why BSMV-mediated HIGS has a similar effect (about 50% transcriptional downregulation) with and without triadimefon treatment? Does BSMV infection affect expression of the CCC1 homologs?

Response: We sincerely thank the reviewer for raising this insightful comment.

In **Supplementary Fig. 5**, we show that triadimefon treatment strongly induces the expression of *PstCCC1* genes (up to 40-fold) within the pathogen. This experiment characterizes the pathogen's direct response to the fungicide. In contrast, the data originally presented in Supplementary Fig. 6 aimed solely to validate the efficiency of the BSMV-HIGS silencing system itself, before any fungicide application. The ~50% transcriptional downregulation was measured in *Pst* samples collected from HIGS-treated wheat plants prior to their exposure to triadimefon. The labels “with” and “without triadimefon treatment” were intended to denote the subsequent phenotypic assay groups, not the timing of the efficiency measurement. We apologize for this confusing labeling.

To eliminate the potential ambiguity regarding timing, we have revised **Supplementary Fig. 6b**. The figure now presents only the pre-treatment silencing efficiency, clearly showing that the HIGS system achieves significant knockdown of the *PstCCC1* genes**.** The comparison labeled “with and without triadimefon treatment” has been removed, as the sole purpose of this experiment was to quantify the silencing level to ensure robust setup for the subsequent phenotype assays.

Fig. S6e shows the iron content or cytosolic iron content (I assume cytosolic)? Please specify. What was the total iron content? It is important to know whether triadimefon treatment increases the total cellular iron content or changes the cellular iron compartmentalization.

Response: We thank the reviewer for this question. In **Fig. S6e**, the measured iron corresponds to cytosolic iron content. Following your suggestion, we additionally measured the total cellular iron content. Our results show that triadimefon treatment does not change the total iron level but significantly increases cytosolic iron, indicating that triadimefon primarily alters iron compartmentalization rather than total iron uptake. We have added the total iron data and updated the description in the revised manuscript.

9) Supplementary Figure 2 and respective text.

What is ”Fielder”?

Response: We thank the reviewer for this question. “Fielder” is a wheat cultivar used in this study, chosen for its well-established high transformation efficiency, which is important for generating *PstCCC1*-RNAi transgenic plants. This rationale is described in the Methods section (**Line 790-791**). To avoid confusion for readers, we have added a brief explanation at the first mention of “Fielder” in the Results section.

As for Supplementary Figure 5, it is puzzling that BSMV-mediated HIGS has the same effect with and without triadimefon treatment given that triadimefon treatment significantly increases the expression of the analyzed genes (Supplementary Fig. S5)?

Response: We thank the reviewer for raising this point. The apparent discrepancy arises from the experimental timeline, as explained in our previous response. The silencing efficiency reported in **Supplementary Fig. S6** and **Fig. 2b** was measured after pathogen inoculation but before triadimefon treatment. The strong induction of

PstCCC1 genes by triadimefon, shown in **Supplementary Fig. S5**, occurs after fungicide exposure.

Thus, the similar ~50% downregulation observed with and without triadimefon reflects the baseline silencing efficiency of the HIGS system prior to fungicide treatment. We have revised the Methods section and the legend of **Fig. 2** to clearly indicate this chronological order and resolve any ambiguity.

Does Fig. 2d show the iron content or cytosolic iron content (I assume cytosolic)? Please specify. It is important to know whether triadimefon treatment increases the total cellular iron content or changes the cellular iron compartmentalization. It would be interesting to analyze the iron content with iron and BPS supplementation (as done in Fig. 3d), respectively!

Response: We sincerely thank the reviewer for this excellent suggestion. You are correct that Fig. 2d (now **Fig. 2f**) depicts the cytosolic Fe^{2+} content, and this has been modified in the revised figure legend.

In response, we performed additional experiments analyzing cytosolic Fe^{2+} in *Pst*-inoculated RNAi plants under triadimefon, triadimefon+Fe^{2+}, and triadimefon+BPS treatments. These new data are integrated into the revised **Fig. 2c-2f**.

The results show that: **1**) Triadimefon+Fe^{2+} treatment synergistically increased cytosolic iron accumulation and caused the most severe fungal growth inhibition. **2**) Addition of the iron chelator BPS reduced cytosolic Fe^{2+} levels and partially rescued fungal growth, demonstrating that cytosolic iron toxicity underlies the enhanced sensitivity of *PstCCC1*-silenced strains. **3**) Total cellular iron content remained unchanged (**Fig. 2e**), indicating that triadimefon affects iron compartmentalization, not total iron uptake. These findings provide genetic and pharmacological evidence linking *PstCCC1* function, iron homeostasis, and triadimefon resistance.

10) Iron determination:

Was this done using crediniospores for Pst or is it possible to purify the hyphal Pst cells from plants? If urediniospores were used, the measurements may not reflect the iron content of hyphal cells. This should be stated. What was the total cellular iron content in all these measurements?

Response: We thank the reviewer for this insightful question. The iron measurements in Supplementary Fig. S6e and Fig. 2f specifically reflect cytosolic iron content in *Pst*. To clarify, total cellular iron content was also measured (now shown in **Supplementary Fig. S6d** and **Fig. 2e**), and these data confirm that triadimefon does not significantly alter total cellular iron, but rather disrupts iron compartmentalization, causing cytosolic accumulation. This distinction supports our mode that vacuolar iron transport, rather than total iron uptake, is key for triadimefon resistance.

Given the technical challenges of quantifying iron in an obligate biotroph, we used enzymatic digestion to enrich fungal structures and rigorously subtracted background iron from healthy plant tissue. Importantly, the core conclusion, that *PstCCC1* mutations enhance vacuolar sequestration to confer resistance, was independently validated in heterologous systems (*Saccharomyces cerevisiae* and *Fusarium graminearum*), providing direct support for the protein's function. We have updated the figure legends and Methods section to clarify these points.

11) Line 226:

According to the "Introduction" section, the Y134F mutation does not confer triadimefon resistance?

Response: We thank the reviewer for raising this important point. The statement in the introduction regarding the Y134F mutation was based on the study by Cook et al. (2021), who used heterologous expression of the *Cyp51* gene from *Pst* in yeast and concluded that the Y134F mutation was not significantly linked to triadimefon resistance. However, that study did not directly assess the fungicide sensitivity of the original *Pst* field isolates carrying this mutation.

In our study, we conducted systematic sensitivity assays on 486 *Pst* isolates collected from major wheat-growing regions in China, combined with *Cyp51* sequencing. We found that the Y134F mutation is indeed associated with moderate resistance to triadimefon, as all 17 moderately resistant isolates carried this mutation. In contrast, 16 low-resistant isolates lacked the Y134F mutation, suggesting the involvement of other resistance mechanisms.

Thus, the Y134F mutation does contribute to triadimefon resistance in *Pst*, but

only in the context of moderately resistant isolates, and its role is context-dependent. We have revised the introduction to clarify this point.

12) Figure 3.

While the data are interesting, they are of limited value as the strains are not isogenic apart from mutations in Cyp51 and CCC1 homologs.

Response: We thank the reviewer for raising this critical point. We acknowledge that the strains used in this study are not isogenic. However, we have taken several approaches to ensure that the observed phenotypes are strongly linked to the specific mutations in *Cyp51* and *CCC1*.

First, there is a consistent and genotype-specific association between the mutations present in each isolate and their corresponding phenotypes. The sensitive isolate carries no relevant mutations; the low-resistance isolate carries only the *PstCCC1* mutation; and the moderately resistant isolate carries the *Cyp51* Y134F mutation but lacks the *PstCCC1* mutation. Their resistance levels and sterol-dependency patterns align precisely with these genotypes, supporting a causal relationship.

Second, key experiments were conducted across multiple independent biological isolates, and the same phenotypes were reproducibly observed. This cross-isolate consistency provides further evidence that the major phenotypic differences are driven by the focal mutations rather than strain-specific background effects.

Finally, we wish to emphasize that the purpose of Fig. 3 is not to compare absolute resistance levels between isolates but to illustrate two mechanistically distinct resistance pathways-one ergosterol-dependent and one vacuolar iron-dependent. Although the isolates are not fully isogenic, this mechanistic distinction remains robust and is additionally supported by our heterologous validation experiments.

Fig. 3c: To which value were the data normalized?

Response: In the revised Fig. 3c,values were normalized to the BSMV:γ for each respective isolate, which was set to 100%. The relative values of all other treatments are presented as a percentage of this internal control. This normalization procedure has now been explicitly described in the updated figure legend for clarity.

Fig. 3d: The sensitive strain should not be called WT since it is not isogenic with the

other strains. What was measured in ”culture solution”?

Response: We have revised the WT to sensitive isolate. The term “culture solution” in the original manuscript specifically referred to a water-agar medium without triadimefon.

13) Line 274/Supplementary Figure 7:

Technical details of the cloning strategy are missing (e.g., primers used, promoter used, etc). Was the GFP-tag N-terminal or C-terminal?

Response: We thank the reviewer for this helpful comment. We have now expanded the Materials and Methods section to include all relevant technical details of the cloning strategy (Line **999-1006**). Specifically, the coding sequences of the candidate genes and their corresponding mutants were cloned into the pDR195 vector under the control of the constitutive LacZ_α promoter. A C-terminal GFP fusion was generated for each construct. In addition, all primer sequences used for cloning have been compiled and added to **Supplementary Table 4**. These additions ensure that the experimental procedures are fully transparent and reproducible.

Fig. 4b/c, yeast strains were grown in SD-U, or? Please specify.

Response: We thank the reviewer for this question. The yeast strains for the iron content assay in **Fig. 4b** and **4c** were pre-cultured in the SD-U medium. After pre-culture, the cells were harvested, washed, and transferred into fresh SD-U medium supplemented with the indicated treatments for a defined incubation period prior to iron quantification. To make this clear, we have revised the figure legend for **Fig. 4** to clearly state the four growth conditions used to assess cytosolic and vacuolar iron levels: (1) SD-U only, (2) SD-U + 20 mM $FeSO_4$, (3) SD-U + 10 μg/mL triadimefon, and (4) SD-U + 20 mM $FeSO_4$ + 10 μg/mL triadimefon. These changes have been incorporated into the revised manuscript (**Line 352-368**).

Do the different mutant strains show differences in resistance to triadimefon without 20 mM iron supplementation? Together with the shown data, this experiment may indicate if external iron stress is required to for the CCC1-mediated triadimefon resistance.

Response: We thank the reviewer for raising this important point. In response, we conducted the proposed experiment to determine whether the mutant strains exhibit

differential sensitivity to triadimefon in the absence of external iron supplementation.

As shown in the revised **Fig. 4**, we treated the *S. cerevisiae* heterologous strains expressing *PstCCC1.1*, *PstCCC1.2*, *PstCCC1.3*, and their corresponding mutant variants with triadimefon alone. The new data reveal a clear inverse relationship between vacuolar iron transport activity and fungicide sensitivity: strains expressing variants with enhanced vacuolar iron sequestration displayed lower sensitivity to triadimefon, whereas strains with impaired transport activity showed greater sensitivity.

Importantly, this pattern is evident even without 20 mM Fe^{2+} supplementation, demonstrating that CCC1-mediated triadimefon resistance does not require additional external iron stress. Instead, the ability to mobilize and compartmentalize endogenous intracellular iron is sufficient to modulate triadimefon susceptibility.

These results strengthen our conclusion by providing direct functional evidence that CCC1-dependent vacuolar iron transport plays a central role in determining triadimefon resistance independently of external iron levels.

14) Line 297:

This is also true for the PstCCC1.1 with and without mutation.

Response: We thank the reviewer for bringing this to our attention. The reviewer is correct that this statement also applies to PstCCC1.1 with and without the mutation. In the original manuscript, this point was obscured by a labeling error in the corresponding figure, where the panels were mistakenly labeled as “a, b, c, e, f” instead of the correct “a, b, c, d, e.” This was an oversight during figure assembly. The correctly labeled experimental data and results remain unchanged and fully support our conclusions. Thank you again for your careful attention to this detail.

15) Supplementary Figure 8/Line 334 and following:

Ferroptosis is a form of regulated cell death characterized by iron-dependent accumulation of lipid peroxidation products. In my opinion, the data presented indicate oxidative stress (including lipid oxidation) amplified by increased cytosolic iron accumulation. I would advise against using the term 'ferroptosis', as it has never been proven in fungi, also not in this study. What is the source or definition of ”ferroptosis”-genes shown in Supplementary Figure 8 - there is no reference.

Response: We thank the reviewer for this important clarification. In accordance with the reviewer's recommendation, we have removed the term "ferroptosis" from the manuscript and from the original Supplementary Figure 8, and we now refer to these processes more generally as "cell death" or "oxidative stress–associated cell death" in the revised **Supplementary Figure 10**. As the reviewer correctly notes, ferroptosis has not been demonstrated in fungi, including in our study, and we agree that using this term would be inappropriate and potentially misleading.

Regarding the genes shown in the previous Supplementary Figure 8, we have clarified their origin and definition. These genes were identified through homology-based annotation using BLAST searches and conserved domain analyses against well-characterized cell death-related genes from *Saccharomyces cerevisiae*, *Aspergillus fumigatus*, and mammalian systems. These homologous genes are associated with oxidative stress responses, lipid peroxidation, and regulated cell death in fungi.

We have revised the Methods section to describe this identification process in detail and added references supporting the known functions of these homologous genes. For the reviewer's convenience, the complete list of cited PMIDs is now provided: 35417721, 24119111, 24382301, 32334817, 38428031, 38110635, 37794028, 30660496, 35998750, 38714881, 21814286, 35985521, 31873291, 21146390.

We appreciate the reviewer's constructive comment, which has helped us refine the terminology and improve the accuracy of the manuscript.

The labelling (e.g., T5/CK5) of the gene expression columns is hardly visible and not explained.

The columns order should follow the time points, i.e., T1, T2, T3, T4, T5

Response: Revised as you suggested, we generated the heatmap using fold change values derived from each group comparison, as this approach allows for the presentation of significantly differentially expressed genes based on the thresholds of $P < 0.05$ and fold change > 2.0. And the columns order has been revised to follow the time points.

The gene-IDs of the presented genes have to be supplied in Supplementary data!

Response: Revised as suggested, we have provided the detained gene-IDs, annotation information, and gene FPKM values in **Supplemental Table S7**.

It is well known that azole treatment is linked to oxidative stress (e.g., PMID: 25445311, PMID: 30126960), which is not mentioned. Therefore, authors should focus on the analysis of genes that have characterized antioxidative roles in fungi, e.g. catalases, peroxidases, superoxide dismutases, lipid peroxidation, Yap1, etc. Moreover, the expression pattern of genes involved in iron acquisition and detoxification (Ccc1) should be shown.

Response: We appreciate the reviewer's insightful suggestion. As recommended, we have examined the expression profiles of genes with established roles in fungal antioxidative responses, including those encoding catalases, peroxidases, superoxide dismutases, and factors involved in lipid peroxidation and oxidative stress signaling (e.g., Yap1). These results are now presented in **Supplementary Fig. 11**. Our analyses show that numerous antioxidant-related genes are differentially expressed following azole treatment, consistent with the well-documented association between azole exposure and oxidative stress. Notably, the number of differentially expressed antioxidant genes progressively decreased during the course of fungicide treatment in the resistant strain, indicating that the oxidative impact of the fungicide diminishes as resistance manifests. Additionally, the expression patterns of genes involved in iron acquisition and detoxification, including CCC1, have been incorporated as requested (**Supplementary Fig. 8**).

16) Supplementary Figure 9, Supplementary Figure 11:

To prove functionality of FgCCC1 in iron detoxification, resistance to iron excess without triadimefon treatment should be analyzed as done in baker´s yeast.

Response: We thank the reviewer for this valuable suggestion. To directly assess the role of *FgCCC1* in iron detoxification, we have now examined the growth of all strains under iron excess conditions in the absence of triadimefon treatment following the approach established in *S. cerevisiae*. In addition, we quantified both the cytosolic and total iron content in these strains.

Furthermore, to strengthen our conclusion, we expanded the analyses by testing the sensitivity of the transformants to additional triazole fungicides (hexaconazole and tebuconazole) and to a fungicide with a distinct mode of action (flueneteram). The cytosolic and total iron measurements for these conditions were also included. These results, now presented in the revised **Supplementary Fig. 13**, demonstrate that the function of FgCCC1 in iron detoxification and resistance is conserved.

All these new data have been incorporated into the revised manuscript and figures. Together, these findings provide comprehensive evidences supporting the role of FgCCC1 in iron homeostasis and its contribution to triazole resistance.

17) Supplementary Figure 11:

Fig. S11 a:

Important technical details for generation of the mutant strains are missing: Integration at the homologous locus or ectopically (identical locus)? Integration in single or multiple copy. Which promoter was used? Anyway, the PH-1 strain is not the right control; a strain with integration of the non-mutated gene should be used.

Response: We thank the reviewer for these important technical questions. In response, we have revised the Methods section to include the missing details. The complementation strains were generated by ectopic integration of the respective genes into the ΔFgCCC1 mutant background, resulting in multiple copy insertions. The promoter used was the native *FgCCC1* promoter to ensure physiologically relevant expression levels.

We also replaced the previously used PH-1 strain with the appropriate control, i.e., a ΔFgCCC1 strain complemented with the non-mutated FgCCC1 gene, as suggested. To confirm the functional expression in the complemented strains, we performed quantitative RT-PCR, which showed that the expression levels of the candidate genes and their mutant versions were significantly higher (over 1.5-fold) than the native FgCCC1 transcript in PH- (see **Fig. R4** below). These results confirm successful overexpression and validate the functional assays performed with these strains.

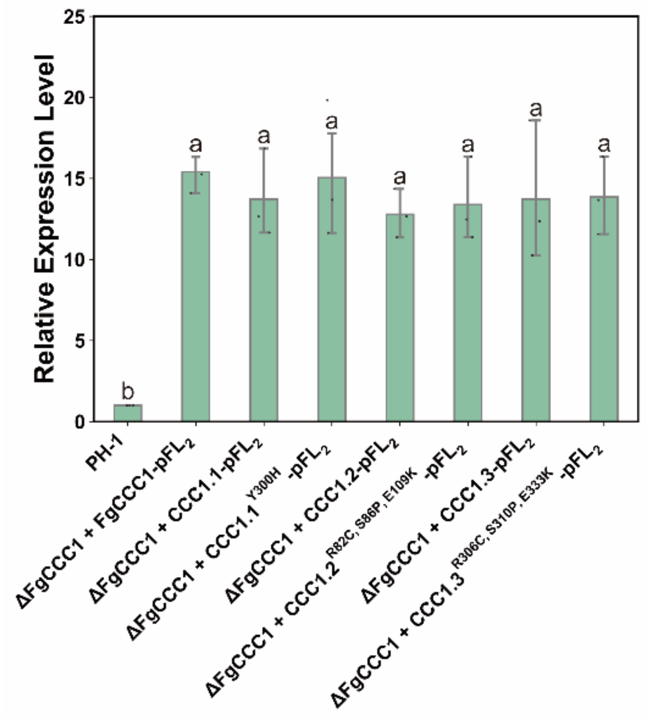

Fig. R4 Expression analysis of candidate genes and their mutant alleles in heterologous overexpression strains. Quantitative RT-PCR analysis showing transcript levels of the three candidate genes (*PstCCC1.1*, *PstCCC1.2*, and *PstCCC1.3*) and their corresponding mutant versions in the *Fusarium graminearum* overexpression strains. Transcript levels were normalized to the *FgActin* reference gene and are presented relative to the expression level of the native FgCCC1 in the wild-type PH-1 strain. Data are presented as mean ± SD from three biological replicates; Different letters indicate significant differences as determined by one-way ANOVA with Duncan's new multiple range test ($P < 0.05$).

Fig. S11b/c: How was growth recorded – radial growth? How were the data normalized? Why do some of the mutant strain exhibit significant differences in radial growth on MM? I do not really see the quantified triadimefon inhibition of mycelial growth in Fig. S11a Convincing is only the difference between CCC1.3 not-mutated/mutated). I appreciate to see original pictures of fungal colonies but I suggest to analyze the triadimefon resistance by classical determination of the "minimal inhibitory concentration (MIC)". To prove functionality in iron detoxification of Pst1 CCC1 paralogs in F. graminearum, resistance to iron excess without triadimefon treatment should be analyzed as done in baker´s yeast.

Response: We thank the reviewer for these constructive suggestions. . We have addressed them in the revised manuscript as follows:

1. Quantitative assessment of fungicide resistance: We now report triadimefon sensitivity using EC_{50} (half maximal inhibitory concentration) values, which provide a more rigorous measure than visual inspection of colony size.

2. Growth measurement: All growth assays, including fungicide and iron treatments, were recorded as radial growth on solid media. Data were normalized as percent mycelial growth relative to the untreated control for each strain.

3. Iron detoxification assays: To demonstrate the role of PstCCC1 paralogs in iron homeostasis, we performed classical iron excess assays without fungicide, similar to studies in baker's yeast. We also analyzed growth under triadimefon alone and in combination with Fe^{2+} or the iron chelator BPS.

4. Additional fungicide tests: To strengthen our conclusions, we included assays with two additional triazole fungicides and one fungicide with a different mode of action.

These revisions, including representative colony images, EC_{50} calculations, and iron detoxification data, have been incorporated into revised **Supplementary Fig. S11** and the Methods section.

18) Line 440 and following:

The methodology used for expression of the PstCCC1 homologs is not suitable for membrane proteins. Therefore, the 3D structure of the protein is most likely wrong and not suitable for further analyses.

Response: We thank the reviewer for raising this important point. We agree that predicting accurate 3D structures for membrane proteins is inherently challenging due to their complex topology and hydrophobic nature.

However, recent advances in deep learning-based structure prediction, particularly AlphaFold 3, have significantly improved the reliability of models for membrane proteins. Several high-profile studies have demonstrated that AlphaFold 3 can generate highly accurate models for various membrane protein families, whose predictions were later confirmed by experimental structures (PMID: 36462505; 39576860; 39110804). These benchmarks indicate that while cautious interpretation is necessary, high-

confidence AlphaFold 3 predictions are valuable for guiding functional hypotheses and comparative analyses.

In fact, the 3D structures of the PstCCC1 proteins were predicted using AlphaFold 3, which does not require experimental protein expression. To validate these models, we compared the predicted structures with homologous proteins whose crystal structures have been experimentally determined. The results show that the predicted PstCCC1 structures exhibit high similarity to their experimentally resolved homologs, supporting the reliability of our AlphaFold models. Therefore, these structures are suitable for downstream comparative and functional analysis.

19) Line 500:

E333 from PstCCC1.3 is conserved neither in FG nor SC.

Response: We sincerely thank the reviewer for pointing out this inaccuracy. We have revised the manuscript to accurately reflect this. Upon re-examining our multiple sequence alignment data (**Supplementary Fig. 18**), we confirm that the E333 residue in PstCCC1.3 is indeed not conserved in *F. graminearum* (*Fg*) nor *S. cerevisiae* (*Sc*). We have therefore revised the manuscript to accurately reflect this. The conservation we intended to highlight pertains primarily to the R306 and S310 in PstCCC1.3 (corresponding to R181 and S185 in FgCCC1), which were validated in our functional experiments in **Fig. 7**). The text has been corrected to remove the incorrect statement regarding E333.

20) Figure 7, Line509.

Important technical details for generation of the mutant strains are missing: the mutated FgCCC1 gene has been introduced into the FgCCC1-lacking mutant: how was this done? Integration at the homologous locus or ectopically (identical locus)? Integration in single or multiple copy. Which promoter was used - the original promoter? Anyway, the PH-1 strain is not the right control; a strain with integration of the non-mutated gene should be used.

Response: We thank the reviewer for requesting these important technical clarifications. The mutated and wild-type FgCCC1 genes were introduced into the ΔFgCCC1 mutant via ectopic integration, resulting in multiple copies expressed under the strong RP27

promoter. We agree that the PH-1 wild-type strain is not an appropriate control for assessing the effects of these mutations. Accordingly, we generated new control strains by ectopically integrating the corresponding non-mutated FgCCC1 genes into the same ΔFgCCC1 background. These control strains have been used in all comparative analyses presented in the revised **Fig. 7**, and the Methods section has been updated to include full technical details of the strain construction (**Line 1039-1047**).

21) Line 613:

”low resistant” not ”resistant”

Response: Revised as suggested.

22) Line 654:

Please replace ”ion” by ”calcium”

Response: Revised as suggested.

23) Line 659:

Refs 34 and 36 do not report release of free iron ions by contributing to iron homeostasis imbalance.

Response: We sincerely thank the reviewer for correction. We agree that references 34 and 36 primarily focus on the role of V-ATPase and ergosterol in azole sensitivity and do not directly demonstrate the “release of free iron ions” as a mechanism. To address this concern and to strengthen our conclusion with a more accurate and well-supported mechanism, we have revised the manuscript accordingly to avoid the misleading description that the release of free iron ions by contributing to iron homeostasis imbalance (**Line 693-696**).

Furthermore, we now describe the disruption of iron homeostasis as a fundamental consequence of targeting the heme-containing enzyme Cyp51. This revised narrative is well supported by a large body of evidence, which we now cite using a recent, comprehensive review that explicitly details the intricate link between azole action and cellular iron homeostasis (Van Genechten et al., 2023, FEBS J.). This reference provides direct theoretical foundation for our hypothesis that DMI fungicides disrupt iron homeostasis.

24) Line 659:

Ref 38 does not fit to the statement and should be replaced by PMID: 23151814 and PMID: 25092765.

Response: Revised as suggested.

25) Figure 8:

According to the "Introduction" section, the Y134F mutation does not confer triadimefon resistance? What causes moderate triadimefon resistance?

Response: We appreciate the reviewer's insightful question. Sorry for this discrepancy due to this unclear description. Now we revised the Introduction to clearly clarify that the Y134F mutation in *CYP51* confers triadimefon resistance (**Line 36-41**).

Please avoid the terms Wild-type and ferroptosis.

Response: Revised as suggested.

The schemes do not differ apart from the marked mutations – the read out is always the same – this is not very intuitive.

Response: We thank the reviewer for the constructive feedback regarding the clarity of our model schematic. The updated scheme now clearly distinguishes the two distinct resistance mechanisms: (1) the Cyp51 Y134F-mediated moderate resistance, and (2) the CCC1 paralog–mediated low resistance. Visual cues, such as color coding and separate pathways, have been added to emphasize the functional differences between these mechanisms, making the readout more immediately understandable.

26) Line 570:

The Discussion should be made more concise and compact.

Response: We sincerely thank the reviewer for this constructive suggestion. We have thoroughly revised the Discussion section to make it more concise and focused. The revisions were highlighted for your convenient tracking.

Reviewer #2 (Remarks to the Author):

This manuscript reports on the intriguing discovery through genetic mapping of polymorphisms in the vacuolar iron transporter family CCC1 in one of the major pathogens of wheat as conferring increased tolerance to a commonly used fungicide. The fungicide used, triadimefon, is part of the demethylation inhibitor/azole class, the

most widely used set of chemicals to control fungal diseases in agriculture and human health.

The authors then follow up this discovery with an extensive and substantial set of experiments towards establishing how this might be occurring, including using the model fungus Saccharomyces cerevisiae for heterologous expression, and then showing similar changes in resistance occur in an evolutionarily-diverse plant pathogen, *Fusarium graminearum*. Of note the discoveries and many of the analyses were made in an obligate plant pathogen, making much of the work technically challenging.

Hence, this research could be of wide appeal to those exploring mechanisms of resistance to azole fungicides or other genetic aspects in fungi that might mean they are less (or ideally more) effective in treating diseases.

One major point that stands out is that only a single example of this fungicide class was tested. The findings would be considerably stronger if this were a general feature across, as an example, other commonly used DMI fungicides in agriculture.

Response: We thank the reviewer for this valuable question. To test if the CCC1 mutation confers resistance only to triadimefon or to other triazoles, we assessed sensitivity to two other triazole fungicides, tebuconazole and hexaconazole. We also included the SDHI fungicide flubeneteram as a control. These tests were performed in both the heterologous *Fusarium graminearum* system and in *Pst* RNAi-silenced lines.

Our new data show (**Fig. 2**, **Fig. 7**, and **Supplementary Fig. 13**) that the mutant CCC1 alleles confer reduced sensitivity to all three triazole fungicides. In contrast, sensitivity to flubeneteram was not affected. This demonstrates that the CCC1-mediated resistance mechanism provides cross-resistance to multiple triazole drugs. It is not specific to triadimefon.

We have added these new results and the corresponding figures to the revised manuscript.

Medium points for consideration:

In figure 4, what is impact of triadimefon used alone?

Response: We thank the reviewer for raising this important point. To address it, we conducted the experiment using triadimefon alone on the *S. cerevisiae* heterologous

strains expressing PstCCC1.1, PstCCC1.2, PstCCC1.3, and their respective mutants. As shown in the **revised Fig. 4**, the results reveal a clear inverse correlation between vacuolar iron transport activity and fungicide sensitivity: strains with enhanced vacuolar iron sequestration exhibited reduced sensitivity to triadimefon. These findings directly demonstrate a functional link between vacuolar iron transport activity and triadimefon susceptibility, even in the absence of additional iron supplementation, thereby strengthening our mechanistic conclusions.

The model to explain the mechanism is unclear - how is this working to provide resistance? The text is vague, about the well-known interaction between the DMI fungicides an iron (heme) cofactor within it. A discussion on the impacts of iron on Candida albicans, and no change in DMI resistance is covered in Van Genchten et al. 2024 FEBS J. Or more broadly, is there additional literature available to explain/support the model?

Response: We sincerely thank the reviewer for this insightful comment, which has helped us clarify the mechanistic model and strengthen the scholarly foundation of our manuscript. In response, we have revised the Discussion to:

1. **We have substantially revised the Discussion section,** which provide a clearer, stepwise explanation linking DMI fungicide inhibition of the heme-containing Cyp51 enzyme to cytosolic iron accumulation, oxidative stress, and cell death, and to show how enhanced vacuolar iron sequestration by mutated *PstCCC1* transporters mitigates this stress to confer resistance.

2. **Clearly address the findings of Van Genchten et al. (2024, FEBS J.)**, discussed potential reasons for the apparent discrepancy between Candida albicans and *Pst*, including pathogen-specific adaptations in iron homeostasis and the unique role of vacuolar compartmentalization in plant-pathogenic fungi.

3. **Incorporate additional literature supporting our model**, added studies on iron homeostasis transcription factors (e.g., HapX, SrbA) in azole tolerance and the conserved function of CCC1-type transporters in iron detoxification across fungi.

These revisions clarify the mechanistic rationale and firmly situate our findings within the broader context of fungal stress biology.

Minor points:

General: this focus is on China (e.g. first line of the abstract), but is this an issue globally? To follow on from this, in addition to the analysis of the 34 isolates of Pst previously sequenced from China there are likely other genome sequences of Pst available beyond those examined in this research that might carry these polymorphisms.

Response: Thank you for raising this important point. Our study initially focused on Chinese isolates of *Pst* due to the high prevalence of triadimefon resistance there. The resistance mechanism we found, involving mutations in vacuolar iron transporter genes *PstCCC1.2* and *PstCCC1.3*, is likely relevant globally.

Evidence from Cook et al. (2021) shows that the Cyp51 Y134F mutation, a known DMI resistance marker, occurs at high frequency (50-60%) in *Pst* isolates from both China and New Zealand. This indicates that DMI selection pressure is widespread. While our study did not screen globally for *PstCCC1* mutations, the evolutionary conservation of the CCC1-mediated resistance mechanism (as seen in *Fusarium graminearum*) suggests similar mutations could arise in *Pst* populations worldwide under comparable fungicide pressure.

Cook et al. further identified both *Cyp51* and *SdhC* resistance mutations in geographically dispersed *Pst* populations (363 isolates from 24 countries), demonstrating that such mutations can emerge independently across genetic backgrounds. Although our work focused on Chinese isolates, we recognize the need for broader global surveillance to determine the prevalence of *PstCCC1* mutations.

Many more *Pst* genome sequences are available from regions with intensive fungicide use, such as Russia, India, Australia, and Europe. Future studies should therefore include wider geographic sampling to assess the global distribution of *PstCCC1* mutations and their role in DMI resistance.

In summary, while our study originated from observations in China, the mechanism we describe is evolutionarily conserved and likely operational in other regions where DMI fungicides are widely used. We have expanded the Discussion section in our revised manuscript to explicitly address the potential global relevance of

the PstCCC1-mediated resistance mechanism (**Line 731-738**).

The Introduction is an extended Abstract, so should be re-written.

Response: We sincerely thank the reviewer for this insightful comment. In response, we have thoroughly rewritten and reorganized the Introduction to provide a clearer conceptual framework for the study. The revised version now opens with the broader context of wheat stripe rust and its management, then narrows to the specific issue of DMI fungicide resistance beyond *Cyp51* mutations, clearly articulates the existing knowledge gap, and concludes with our hypothesis and experimental strategy. We have removed all elements resembling a summary of results to ensure that the section functions as a proper introduction rather than an extended abstract.

What is the origin of the F. graminearum CCC1 mutant strain?

Response: We thank the reviewer for this important question regarding the origin of the *Fusarium graminearum* (*Fg*) CCC1 mutant strain. The F*gCCC1* mutant used in this study (i.e., the complemented strain carrying the site-directed mutations) was generated in vitro as part of this work. Specifically:

1.**Knockout Mutant (ΔFgCCC1)**: The homologous gene deletion mutant ΔFgCCC1 was the recipient strain. The origin and construction of this knockout mutant are described in the **Methods section (line 1019-1027)**.

2. **Design and synthesis of the mutant allele:** First, a multiple sequence alignment confirmed that the critical mutation sites in *PstCCC1.1*, *PstCCC1.2*, and *PstCCC1.3* are evolutionarily conserved in *Fg* and *S. cerevisiae*. Next, we employed FoldX-based saturation mutagenesis on *FgCCC1*, pinpointing R181P and S185D as the most impactful mutations on protein structure. Finally, to functionally assess these mutations, we commissioned Sangon Biotech (Shanghai, China) to chemically synthesize an FgCCC1 gene encoding the R181P and S185D variants for complementation into the ΔFgCCC1 mutant.

3. **Complementation**: The synthesized mutant gene ($FgCCC1^{R181P,S185D}$) was cloned into the pFL2 vector. This recombinant plasmid was then transformed into the protoplasts of the ΔFgCCC1 knockout mutant to generate the complemented mutant strain.

We have now described the origin and construction process of this specific mutant strain in the revised Methods section (**line 1019-1027; 1039-1049**).

Line 468: the structures were not ‘determined’ but ‘predicted’. The sentence could be ‘We then predicted the structures and interactions of the vacuolar…’ and on line 473: ‘assay identified an interaction between’.

Response: Revised as suggested.

Text:

Line 80: ‘levels that ranged’.

Response: Revised as suggested.

Lines 83-86: one can propose a segregation pattern, but the chi-square test only supports it.

Response: We thank the reviewer for this insightful comment regarding the precise interpretation of the chi-square analysis. We agree that the chi-square test does not prove a hypothesis but assesses whether the observed data are consistent with it. Accordingly, we have revised the wording in **Line 94-96** to state that the chi-square test supports a single-locus segregation model it.

Line 94: missing information to add.

Response: We thank the reviewer for pointing out this omission. We have added the relevant information at the corresponding location in the manuscripts (**Line 105**).

Line 95: duplication on ‘polymorphic’ and the ‘P’ in ‘SNP’.

Response: Revised.

Lines 98-99: add a space after the numbers and ‘bp’.

Response: Revised as suggested.

Lines 125 and 131: clarify the meaning of ‘sense mutation’.

Response: Thank you for your valuable suggestion. We sincerely apologize for the lack of clarity in the term “sense mutation” used in the original manuscripts. In the context of the manuscripts, “sense mutation” actually refers to a “missense mutation”, specifically a nucleotide substitution that results in an amino acid change. We have thoroughly reviewed the entire manuscripts and corrected the mistake.

Line 170: people will not know what this PDS assay is, so will need a phrase to

introduce it.

Response: We thank the reviewer for pointing out the need for clarity regarding the PDS assay. We have now added a brief description at its first mention in the manuscripts.

Line 186: ‘confirm’.

Response: Revised as suggested.

Lines 186-202: needs re-writing as confusing as to what was transgenic. It mixes ‘isolate’ (fungal) with ‘line’ (plant). It seems like the plant was transgenic expressing an RNAi molecule to target the fungus. Line 94 probably ‘impact on disease relative to the untransformed control’.

Response: We sincerely thank the reviewer for this important comment. We apologize for the confusion caused by the original wording. To clarify, the transgenic materials were wheat plants (*Triticum aestivum* cv. Fielder) engineered to express RNAi constructs targeting the *Pst* genes (*PstCCC1.1*, *PstCCC1.2*, and *PstCCC1.3*). The fungal isolate (YQ324) used for inoculation was wild-type and was not genetically modified.

We have revised this section of the manuscript (now **Lines 211–226**) to clearly distinguish between the transgenic wheat lines and the fungal isolate, and to accurately describe the experimental design without mixing terminology such as “isolate” and “line.”

In addition, we have revised Line 194 (now **line 219**) to correctly state “untransformed control.”

Line 387: first mention of ‘Fg’ but the species is not written out.

Response: We sincerely thank the reviewer for pointing out this oversight. We have now revised the manuscript to include the full name of *Fusarium graminearum* at its first mention, followed by the abbreviation ‘*Fg*’.

Line 390: ‘knockout of FgCCC1 and complementation in the deltaFgCCC1 mutant’.

Response: Revised as suggested.

Line 567: ‘mutation’ spelling.

Response: Revised as suggested.

Line 574: ‘its’ for ‘their’.

Response: Revised as suggested.

Line 614: ‘aggregata’ spelling.

Response: Corrected.

Line 739: add italics to the species.

Response: Revised as suggested.

Line 771: ‘Petri’ spelling.

Response: Corrected.

Line 794: ‘Quantitative’ spelling.

Response: Corrected.

Lines 807-811: unclear what ‘RNA sequenc[i]es’ means, i.e. RT clones?

Response: We thank the reviewer for pointing out this. To clarify, we meant cDNA sequences obtained through reverse transcription of RNA extracted from *Pst*-infected wheat leaves. We have revised the text to state that the genes were amplified from cDNA (not genomic DNA) for the purpose of cloning into the plasmid (**line 882-884**).

Line 841: add something in the main text about the WGA to explain how the fungus was visualized under a fluorescence microscope.

Response: We thank the reviewer for this helpful suggestion. We have revised the Methods section (**Line 928-930**) to include a brief description of Wheat Germ Agglutinin (WGA) staining. The added text clarifies that WGA is a fluorescently labeled lectin that specifically binds to N-acetylglucosamine residues in fungal cell-wall chitin, enabling clear visualization of the fungal structures under a fluorescence microscope.

Figures: a general comment much of the font will be too small if some aims to read this as a printed out version as well as the figures in the supplemental material.

Response: We thank the reviewer for highlighting this important issue. We have carefully reviewed all figures in both the main text and the supplemental materials and have uniformly increased the font size for all labels, axis text, and legends.

Figures S2 is too small, and panel a the nucleotides do not align.

Response: We thank the reviewer for these specific comments. **Fig. S2** has been revised accordingly.

Reviewer #3 (Remarks to the Author):

Since fungal pathogens are rapidly evolving resistance to chemical fungicides with single-site modes of action, it is important to elucidate molecular mechanisms responsible for the resistance. In this study, the authors have identified a novel mechanism for resistance to a triazole fungicide triadimefon widely used in China for management of the stripe rust disease caused by Puccinia striiformis f. sp. tritici (Pst) in wheat. The results reported in this study are interesting because the mechanism of resistance to this triazole fungicide in specific isolates of this pathogen does not involve mutations in the Cyp51 gene as reported by a number of earlier studies. Instead, the authors have found that mutations in the genes encoding the vacuolar iron transporters are responsible for resistance to triadimefon in a few Pst isolates previously collected from the wheat fields in China. This finding is significant because it identifies a novel mechanism for fungicide resistance in an obligate biotrophic basidiomycete pathogen which is difficult to study.

Using bulked segregant analysis and genetic mapping, authors identified three candidate iron transporter genes PstCCC1.1, PstCCC1.2 and PstCCC1.3 on chromosome 6 as candidates for triadimefon resistance in Pst. Silencing these genes reduced triadimefon resistance, while sequence analysis identified specific mutations (PstCCC1.2R82C,S86P,E109K and PstCCC1.3R306C,S310P,E333K) in PstCCC1.2 and PstCCC1.3 that enhanced iron transport. Heterologous overexpression of these candidate genes and their mutants in yeast and the ascomycete fungal pathogen Fusarium graminearum (Fg) confirmed their role in vacuolar iron transport and triadimefon sensitivity. The cytosolic iron contents in Pst isolates overexpressing these mutants were significantly lower than those in the wild-type isolates, thus providing correlation with decreased sensitivity to triadimefon. In vitro assays using microscale thermophoresis (MST) and isothermal titration calorimetry (ITC) further validated these observations, showing that specific mutations in PstCCC1.2 and PstCCC1.3 enhance iron transport into vacuole and detoxification conferring low resistance to triadimefon.

Overall, the authors have used a solid mix of genetic mapping, gene expression analysis, functional experiments, and validation across multiple fungal species to provide insight into Cyp51-independent mechanism of fungicide resistance. They have provided compelling evidence for existence of this novel mechanism of fungicide resistance. Conceptually and technically, the study is sound and experiments have been conducted with rigor and correctly interpreted. However, some parts of the manuscript such as rationale behind the isolate selection, distinction between iron content and transport, and significance of ergosterol for fungicide resistance need clearer explanations.

Response: We sincerely thank the reviewers for their positive and constructive comments on our manuscript. We have carefully considered all the suggestions and have revised the manuscript accordingly to provide clearer explanations. The point-by-point responses are detailed below.

1. Rationale behind the isolate selection： We have now expanded the Introduction and Methods sections to describe why the specific isolates (YQ324 and Gui1-2) were chosen for genetic crossing. Briefly, YQ324 exhibits triadimefon resistance without carrying the common Cyp51-Y134F mutation or showing target gene overexpression, suggesting a novel, Cyp51-independent resistance mechanism. In contrast, Gui1-2 is highly sensitive and shares an identical Cyp51 sequence with YQ324. This contrast makes their progeny ideal for mapping non-target mechanisms of resistance.

2. Distinction between iron content and transport： We have revised the Results and Discussion sections to more clearly distinguish between cellular iron content and the transport activity of the vacuolar iron transporters. We have clarified in the text that the observed reduction in cytosolic iron content reflects enhanced vacuolar sequestration activity in the mutants, thereby explicitly distinguishing between the measured iron distribution (content) and the underlying transport activity. This efficient sequestration detoxifies the cytoplasm, reduces iron-mediated oxidative stress and thereby increases fungicide tolerance.

3. Significance of ergosterol for fungicide resistance： We agree with the reviewer that the role of ergosterol depletion in triggering iron dyshomeostasis is a key concept

that deserved a more thorough explanation. We have expanded the Discussion to elaborate on how DMI fungicides like triadimefon inhibit ergosterol biosynthesis, leading to impaired membrane integrity and dysfunction of membrane-associated proteins like the vacuolar H^+-ATPase (V-ATPase). This disruption compromises the proton gradient necessary for secondary transport and ion homeostasis, ultimately leading to cytosolic iron accumulation and ferroptosis. We believe these revisions have significantly improved the clarity and conceptual depth of our manuscript.

Lines 7-11: “These mutants exhibited decreased cellular iron content and increased triadimefon tolerance.” Which mutants? In the sentence before authors say heterologous expression of natural mutants in yeast and Fusarium enhances iron transport. I think these two sentences seem confusing/contradictory on iron content and transport part. Authors may need to add more clarification if iron transport is into vacuoles, thereby reducing cytosolic iron? This part on iron transport and iron content should be clarified throughout the manuscript, it is a bit confusing.

Response: We thank the reviewer for raising this important point. The mutants of PstCCC1.2 and PstCCC1.3 enhance vacuolar iron sequestration, which leads to decreased cytosolic iron content and thereby reduces iron-mediated toxicity. This mechanism ultimately confers increased resistance to triadimefon. We have revised throughout the manuscript to clarify this point (**Line 8-13**).

Lines 35-41: The authors should clarify whether the single-nucleotide mutation at 401 directly translates to the Y134F substitution in Cyp51.

Response: We thank the reviewer for requesting this clarification. Yes, the single-nucleotide mutation at position 401 in the *Cyp51* gene directly results in the Y134F amino acid substitution. We have revised the Introduction to clarify this direct nucleotide-to-amino acid relationship and its context-dependent effect on resistance (**Line 36-41**).

Lines 41-51: The authors present a rationale for investigating non-Cyp51-mediated resistance to triadimefon, citing field evidence that some Pst isolates exhibit resistance without mutations or expression changes in Cyp51. However, the authors should

explain why a controlled cross was chosen over direct analysis of naturally resistant field isolates? While genetic tractability is a valid reason, this choice may limit the ecological relevance unless explicitly justified.

Response: We sincerely thank the reviewer for raising this important point. The choice to use a controlled cross rather than direct analysis of naturally resistant field isolates was based on several strategic and methodological considerations:

1. Genetic Purity and Reduced Background Noise: Naturally resistant *Pst* isolates often carry complex, heterogeneous genetic backgrounds, including unknown mutations or mixed populations. A controlled cross between a well-characterized resistant isolate (YQ324) and a sensitive isolate (Gui1-2) produced a segregating population with a clear genetic structure, allowing precise mapping of resistance loci.

2. **Identification of Monogenic Traits:** The triadimefon resistance segregated in a single-locus pattern (3:1 in the F_2 population). This simplified architecture enabled efficient identification of candidate genes via bulked segregant analysis and transcriptomics, avoiding confounding effects of polygenic resistance present in field isolates.

3. **Functional Validation in a Uniform Background:** Progeny from a controlled cross share a consistent genetic background, facilitating reliable functional validation of candidate genes through HIGS or heterologous expression. This strengthens causal links between mutations and resistance phenotypes.

4. **Complementary Field Validation:** To ensure ecological relevance, we tested 38 field isolates, confirming that the identified mutations in PstCCC1.2 and PstCCC1.3 also occur naturally and correlate with triadimefon resistance (**Table 1**).

5. **Tractability and Reproducibility:** As *Pst* is an obligate biotroph, controlled crosses on barberry under greenhouse conditions provide reproducible, genetically defined progeny, which is not feasible with genetically heterogeneous field isolates.

We have now presented this rationale in the Introduction to explicitly justify why a controlled cross was used and how it complements analyses of natural populations (**Line 69-74**).

Why was the fungal biomass only detected after 10 days of triadimefon treatment?

What happens if you go beyond that timepoint or before that?

Response: We thank the reviewer for this question. The 10-day post-treatment timepoint for biomass assessment was chosen because it coincides with peak of disease development in our experimental system. At this stage, untreated control plants show full sporulation, providing a clear baseline to assess the maximum inhibitory effect of triadimefon. This timing has been validated as optimal in our previous study (Ji et al., 2023) and ensures a reliable and standardized measurement of fungal biomass and fungicide resistance. We have provided this rationale in the revised Methods section (**Line 913-917**).

How was the relative inhibition rate of fungicide calculated in Fig3c as well as other figures? It is not mentioned anywhere.

Response: We thank the reviewer for pointing out this omission. The method for calculating the relative inhibition rate has now been added in the Methods section (subsection: **Fungicide sensitivity assay and EC_{50} determination**) of the revised manuscript (**Line 844-855**).

Lines 249–252: The statement that "exogenous ergosterol supplementation effectively reduced the cytoplasmic Fe^{2+} levels in all isolates compared to fungicide treatment alone" does not appear to be supported by the data in Figure 3d. In particular, for the moderately resistant (MR) isolate, the Fe^{2+} content under triadimefon + ergosterol treatment seems slightly higher than under triadimefon alone. This contradicts the claim made by authors. To clarify this discrepancy, I recommend the authors provide the actual mean ± SD values for Fe^{2+} content across all treatment groups. Additionally, statistical significance should be indicated to support the interpretation (for comparison of triadimefon and triadimefon+ergosterl treatments). If the differences are not statistically significant, the phrasing should be revised accordingly.

Response: We thank the reviewer for this critical observation. To address this issue, we have: 1) Removed the original claim and the corresponding Fig. 3d from the manuscript. 2) Replaced it with new experiments that directly quantify endogenous ergosterol levels alongside cytosolic Fe^{2+} content in all isolates, without relying on exogenous supplementation (**Fig. 3d-3e**). 3) Included actual mean ± SD values and appropriate

statistical analyses for all treatment groups to accurately reflect significant differences. This revision provides a more robust and reliable assessment of the relationship between ergosterol levels and iron compartmentalization, eliminating the previous unsupported claim.

Was ergosterol supplementation tested across a concentration gradient? A dose-dependent change in Fe content might further clarify the role of dependence of moderately resistant (MR) isolate to ergosterol for fungicide resistance.

Response: We appreciate this suggestion. We initially attempted exogenous ergosterol supplementation across a concentration gradient; however, the results were highly inconsistent, likely due to poor uptake and uneven distribution of this lipophilic compound in plant tissue hosting the obligate biotroph.

To circumvent this limitation, we implemented a genetic "dose-response" strategy by measuring endogenous ergosterol under four different conditions: with or without *Cyp51* knockdown, and with or without triadimefon treatment. This effectively mimicries varying ergosterol availability (**Fig. 3d**): 1) Group 3 (triadimefon alone): The MR isolate maintains higher ergosterol than the LR isolate. 2) Group 4 (Cyp51 knockdown + triadimefon): The MR isolate's resistance is specifically compromised, demonstrating its dependence on high ergosterol levels. The LR isolate remains resistant, showing independence from this pathway. This approach provides a robust and physiologically relevant demonstration of ergosterol dependence in MR isolates.

Can the authors distinguish whether ergosterol supplementation restores membrane integrity (thereby reducing iron influx) or directly modulates iron transport mechanisms? Inclusion of membrane permeability assays or expression data for iron transporters would be informative. Overall, the conclusion made on "MR isolate are more dependent on ergosterol for fungicide resistance" warrants further exploration.

Response: We thank the reviewer for this excellent point. Our integrated analysis of endogenous ergosterol and cytosolic Fe^{2+} provides mechanistic clarity regarding the distinct resistance strategies of MR and LR isolates (**Fig. 3d-3e**): In Group 4 (Cyp51 knockdown + triadimefon), both MR and LR isolates show similar resistance

phenotypes and comparably low cytosolic Fe^{2+} levels, but the mechanisms differ. **1**) The MR isolate maintains higher ergosterol levels, preserving membrane integrity and thereby limiting iron influx. 2) The LR isolate achieves low cytosolic Fe^{2+} independently of ergosterol, through enhanced PstCCC1-mediated vacuolar iron sequestration, which fully supports fungicide resistance. This evidence demonstrates that the MR isolate's resistance is ergosterol-dependent, whereas the LR isolate relies on a robust iron detoxification pathway, independent of ergosterol.

Minor comments

Line 82: Change (3.18) to (3.18 μg/mL)

Response: Revised as suggested.

Line 82-83: Modify this sentence "The 82 F2 isolates had much wider range of EC50 values ranging from 0.14 to 6.93 μg/mL".

Response: Revised as suggested.

Lines 84-86: The chi squared test ($\chi 2 = 0.27$, $P = 0.60$) segregating into a 3:1 ratio for the resistant (should be sensitive?) (EC50 <1.00) to sensitive (should be resistant?) classes (EC50 > 1.00) (Supplementary Table S3), indicating that the sensitivity of Pst to triadimefon is controlled by a single locus.

Response: We thank the reviewer for pointing out this important oversight. In the original text (Lines 84–86), the greater-than and less-than signs for EC_{50} values were inadvertently reversed, which caused confusion in interpreting resistant versus sensitive classes. We have corrected this so that the resistant class is now correctly defined as EC50 > 1.00 μg/mL and the sensitive class as EC50 < 1.00 μg/mL (**Line 94-95**).

Additionally, we have revised the wording to clarify that the chi-square test supports the consistency of the observed segregation with a 3:1 single-locus model, rather than proving it. This correction ensures accurate statistical interpretation and aligns the text with Supplementary Table S3.

Line 94: Need to add the reference genome used "(add the genome here)"

Response: Revised as suggested.

Line 101: Please specify which three isolates were used for transcriptome analysis. What was the concentration of triadimefon used?

Response: We thank the reviewer for pointing this out. The phrase "three *Pst* isolates" was misleading. For the transcriptome analysis, we used a single low-resistant isolate (YQ324) and collected samples with three biological replicates to analyze the temporal dynamic of the transcriptional response. The concentration of triadimefon applied was 10 μg/mL. We have revised the text to correct this point.

In all figure legends including supplementary figures, the authors should mention that YQ324 isolate is resistant and Gui1-2 isolate is sensitive add details needed in all figure legends including supplementary figures.

Response: Revised as suggested.

Figure 1b describes in detail the SNP index plot. What does each color mean? What does it show? What is the cutoff used to choose chromosome 6 and 11?

Response: We thank the reviewer for this helpful suggestion and for pointing out the need for greater clarity. We have revised the figure legend to include all the details as suggested. In the main panel of **Fig. 1b**, each colored line represents the ΔSNP-index values along individual chromosomes; colors are used only to distinguish chromosomes. The ΔSNP-index visualizes the difference in allele frequency between the resistant and sensitive bulks: values near 0 indicate little difference, while values approaching +1 or –1 indicate strong divergence, suggesting genomic regions associated with triadimefon resistance. For the significance cutoff, a permutation-based approach with 10,000 iterations was used to determine a genome-wide significance at the 99% confidence interval (gray line). Windows exceeding this threshold were considered significant.

Using this criterion, significant peaks were found on chromosomes 6 and 11, defining the candidate intervals: Chromosome 6: two contiguous regions (1.16-1.34 Mb and 2.62-2.80 Mb), merged into a ~0.36 Mb candidate interval; Chromosome 11: two contiguous regions (0.80-1.72 Mb and 1.96-2.14 Mb), merged into a ~1.1 Mb candidate interval.

In the revised figure, the zoomed-in panels for chromosomes 6 and 11, created with TBtools II, provide further detail: the chromosome bar itself displays a blue-to-red gradient representing gene density (low to high), the significant QTL intervals are

marked by red rectangles, and the CCC1 family candidate gene on chromosome 6 is highlighted in red text.

The revisions describe more clearly about the meaning of the colors, the ΔSNP-index plot, and the criteria for selecting candidate regions.

Supplementary Fig. 3c legend does not explain what the dot plot is showing. Figure does not show cluster 1 and 6 separate as described in the legend. This seems like an error. Additionally, expression trend analysis on transcriptome profiles "of jujube at" Pst at distinct temporal phases of triadimefon treatment. Jujube is not mentioned anywhere in the manuscript.

Response: Thanks for your reminder. The legends of **Supplementary Fig. 3b** and **3c** were mistakenly swapped and contained an incorrect reference to "jujube." We have now corrected the legends as follows:

Supplementary Fig. 3b: Expression trend analysis of Pst transcriptome profiles across distinct temporal phases of triadimefon treatment. Two expression modules were highlighted: cluster 1 (genes continuously upregulated) and cluster 6 (genes continuously downregulated) during fungicide exposure.

Supplementary Fig. 3c: Scatter plot showing gene ontology (GO) enrichment for genes in cluster 1 and cluster 6. Colors indicate the significance level of GO terms (blue = low, red = high), and the size of the dots represents the number of genes associated with each pathway.

The revised legends now accurately describe the plots, remove the erroneous "jujube" reference, and clarify the clustering and functional enrichment results.

Supplementary Fig. 6a legend, inoculated "on" the fourth leaves of Sumon 11.

Response: Revised as suggested.

Line 170: The acronyms HIGS and PDS should be spelled out at their first citations in the manuscript.

Response: Revised as suggested.

Lines 173-174: Modify this sentence as "Pst inoculation and triadimefon treatment experiments were conducted on the PDS silenced plants containing the gene silencing constructs for the three candidate genes".

Response: Revised as suggested.

Lines 174-175: Modify this sentence as "The transcription levels of PstCCC1.1, PstCCC1.2 and PstCCC1.3 decreased by 40% - 60% in the Pst-inoculated silenced plants"

Response: Revised as suggested.

Lines 176-177: "but significantly reduced resistance in the triadimefon treatment" not clear what this means. Do the authors mean to say that the plants had increased Pst virulence upon triadimefon treatment.

Response: We thank the reviewer for pointing out this ambiguity. The phrase "reduced resistance" was intended to describe the pathogen's decreased resistance to the fungicide, not the plant's resistance. We have revised the sentence for clarity: "Silencing these genes had no significant effect on *Pst* virulence in the absence of triadimefon, but significantly reduced the fungicide resistance of *Pst* to triadimefon treatment."

Lines 186-187: Modify this sentence as "To further confirm the results of the transient silencing of PstCCC genes, results, transgenic Fielder wheat variety was generated expressing RNAi directed against these genes".

Response: Revised as suggested.

Line 189: State whether T3 plants were homozygous.

Response: We thank the reviewer for raising this important point regarding the genetic stability of the transgenic plants. The T3 generation RNAi transgenic wheat plants (Fielder background) used in the functional validation experiments (**Fig. 2**) were homozygous. Now we have clarified this point in the "Methods" section of the revised manuscript.

Line 198-201: Modify this sentence as "To further determine whether the RNAi transgenic lines inoculated with Pst affected the growth and development of hyphae after treatment with triadimefon, the development of hyphae was observed and measured using a fluorescence microscope".

Response: Revised as suggested.

Line 198: "To" further determine

Response: Revised as suggested.

Fig 2b Statistics on without triadimefon treatment are missing.

Response: We apologize for this oversight. The statistical analysis for the groups without triadimefon treatment has now been added to **Fig. 2b** in the revised manuscript.

Fig 3a legend indicates wild type (WT), moderately resistant (MR), and low resistant (LR) in the legend as well.

Response: Revised as suggested.

Fig. 4b and 4c should be described and cited immediately after Fig. 4a. Please modify the text accordingly.

Response: We thank the reviewer for this helpful suggestion. The description and citation of Fig. 4b and 4c have now been moved to immediately follow the discussion of Fig. 4a, ensuring a more logical and coherent presentation of the results (**Line 341-368**).

Fig. 5c second graph add "T" on top for triadimefon treatment

Response: We thank the reviewer for pointing this out. The label "Tri" has now been added to the second graph of **Fig. 5c** to clearly indicate the triadimefon treatment.

Fig. 8. For the moderately resistant isolate part of the model, the authors state that "This reduces the destructive power of the pathogen cell membrane, and the level of ferroptosis caused by ferrous ion overload inside the cell is greatly reduced compared to the wild-type isolates, resulting in a higher level of triadimefon resistance". However, the model does not show decreased ferroptosis.

Response: We thank the reviewer for this astute observation. The Fig. 8 legend has been revised to more accurately describe the model, focusing on the enhanced integrity of the pathogen cell membrane due to unimpaired ergosterol biosynthesis, without implying a quantified change in ferroptosis, which is not directly shown in the figure.

Authors should provide both excitation and emission wavelength of the fluorescent dyes/channels used in the study in the figure legends including supplementary figures.

Response: We sincerely thank the reviewer for this constructive suggestion. We have now carefully reviewed all relevant figure legends and supplementary figure legends, and the excitation and emission wavelengths were added.

Supplementary Fig. 11a 20 mM $FeSO_4$ or 2 mM $FeSO_4$? In the Results section, authors

have mentioned 20 mM but in figure it appears 2 mM. Also, what does MM stand for?

Response: Thank you for pointing out this inconsistency. The correct $FeSO_4$ concentration used in the experiments is 2 mM, and the figure has been corrected accordingly. "MM" refers to minimal medium, a nutrient-limited medium used to assess fungal growth under controlled conditions. **In the revised Supplementary Fig. 13**, we focused on the results from the basal PDA medium and omitted the MM data, as it was not essential for addressing the main question.

The manuscript will greatly benefit from thorough editorial revision to enhance clarity and to ensure consistency in scientific expression.

Response: We thank the reviewer for this important suggestion. The manuscript has undergone a thorough editorial revision to improve clarity, readability, and consistency in scientific expression. We engaged professional scientific editors to polish grammar, syntax, and overall flow. Key terminology, gene/protein nomenclature, units, and figure citations have been standardized throughout the text. Complex sentences were restructured for clarity, and the overall presentation was refined to enhance coherence and professional quality.

Reviewer #4 (Remarks to the Author):

I co-reviewed this manuscript with one of the reviewers who provided the listed reports. This is part of the Nature Communications initiative to facilitate training in peer review and to provide appropriate recognition for Early Career Researchers who co-review manuscripts.

Response: We are grateful to you for your time and insightful comments, which have significantly helped us improve our manuscript.

POINT-BY-POINT RESPONSE TO REVIEWERS' COMMENTS

Reviewer #1 (Remarks to the Author):

The authors addressed most of my questions and suggestions satisfactorily, with exception of my comment 18.

18) Line 440 and following (original manuscript): The methodology used for expression of the PstCCC1 homologs is not suitable for membrane proteins. Therefore, the 3D structure of the protein is most likely wrong and not suitable for further analyses. I did not question the 3D structure predictions by AlphaFold 3. Rather, I criticised the methodology used to express the PstCCC1 proteins. The authors expressed the proteins in E. coli, followed by a purification protocol intended for hydrophilic proteins, which is not suitable for membrane proteins (Wwhy was PstCCC1 expressed as an MBP fusion protein when smaller affinity tags were used for the others?) As shown by SDS-PAGE analysis (see Supplementary Figure 15), the purification procedure yielded heterogeneous fractions. Therefore, the proteins of the correct size were excised from the SDS-PAGE gels. As SDS-PAGE is a denaturing method, the 3D structures of the produced proteins are most likely incorrect and not suitable for further analysis, such as microscale thermophoresis or isothermal titration calorimetry.

Response: We sincerely thank the reviewer for the comment on the methodology used for expression of the PstCCC1 homologs, which we recognize as essential for ensuring the highest standards in membrane protein research. We respectfully clarify that our actual workflow differs fundamentally from the inference drawn, and we deeply regret that the original Methods section was insufficiently detailed, leading to this misunderstanding. We have now thoroughly revised the section to accurately reflect our experimental approach, which is summarize as follows:

First, the methodology we used is supported by the literature on membrane protein production. All three PstCCC1 proteins and their mutants were produced using an **established *E. coli*-based continuous-exchange cell-free protein synthesis (CFPS) platform coupled with liposome-assisted refolding**. This is the method specifically developed for studying challenging membrane proteins including transporters, ion

channels, and GPCRs. The method has been extensively validated by structural and functional studies (PMID: 37087310; PMID: 27384110; PMID: 38810322; PMID: 37967042; PMID: 39139999; PMID: 41238795; PMID: 39053424). The proteins were synthesized as non-denatured precipitates, solubilized with SDS, and refolded by rapid dilution into pre-formed liposomes, followed by detergent solubilization, affinity purification, and size-exclusion chromatography. The SDS-PAGE gel excision step shown in Supplementary Fig. 15 was performed exclusively on a small analytical aliquot of these purified, refolded proteins for LC-MS/MS-based identity confirmation (>95% sequence coverage) and was not used to recover material for MST or ITC. All biophysical measurements were conducted using the independently purified, liposome-refolded, and buffer-exchanged proteins.

Second, the reviewer's question regarding the use of different affinity tags reflects a critical point, and we have now provided the full optimization procedure in the revised Methods. Prior to final construct selection, we performed in silico hydrophobicity and transmembrane domain predictions, codon-optimized all genes, and systematically tested multiple small fusion tags (His, GST, SUMO) as well as MBP under various expression and refolding conditions. For PstCCC1.2 and PstCCC1.3, small tags (SUMO and His, respectively) were sufficient to obtain soluble, functional protein after CFPS and liposome refolding. For PstCCC1.1, however, none of the small tags yielded detectable soluble protein; only the MBP fusion enabled sufficient recovery.

Third, we fully acknowledge that even liposome-refolded proteins may not perfectly recapitulate the native membrane-embedded conformation. However, several important approaches support the validity of our conclusion. Mutant PstCCC1.2 and PstCCC1.3 confer enhanced iron sequestration does not rest solely on MST and ITC measurements. Rather, this finding is supported by multiple independent, convergent lines of evidence: (i) yeast Δ*ScCCC1* complementation assays showing stronger iron and triadimefon tolerance in mutants, with direct ICP-MS quantification of reduced cytosolic and increased vacuolar iron; (ii) *Fusarium graminearum* Δ*FgCCC1* heterologous expression demonstrating the same phenotype; and (iii) ROS and lipid peroxidation assays revealing significantly lower oxidative damage in

mutant-expressing cells under triadimefon + Fe stress, an effect reversed by the iron chelator BPS. The MST and ITC data serve as biochemical corroboration, demonstrating that the affinity differences observed in living cells can be recapitulated at the molecular level using purified, refolded proteins. Importantly, the consistent positive results from both wild-type and mutant proteins bound iron ions, with mutants showing significantly higher affinity in the direction predicted by our in vivo functional data strongly supports the biological relevance of this affinity difference. Similar multi-evidence strategies have been widely employed in the ion transporter field, where refolded or detergent-purified membrane proteins were used to assess ligand binding affinities that correlated well with functional phenotypes (PMID: 2532670; PMID: 28470611). Thus, while the absolute conformational state may differ from the native environment, the relative binding enhancement of the mutants remains a valid and interpretable finding, fully consistent with all functional datasets.

Fourth, in light of this valuable comment, we have made substantial revisions to avoid the misleading and detail the information. **The Methods section has been completely rewritten** to explicitly describe the CFPS, liposome-assisted refolding, tag screening, and QC workflows, with clear separation between analytical gel excision and preparative protein purification (**Page 45-46, Line 1157-1204**). All relevant literature is now cited to validate the methodology (**Reference 67-68**). **In the Results and Discussion sections**, we have replaced overly definitive statements with more cautious language (e.g., “suggest,” “are consistent with,” “may contribute to”) and explicitly framed the MST/ITC data as supportive biochemical evidence rather than standalone proof of native structure. We are sincerely grateful to the reviewer for pushing us to provide the methodological details with greater precision, which we should have done in the original manuscript.

Minor:

The authors largely eliminated the use of the term ferroptosis but at Line 745 the term ”ferroptotic cascade” is till used.

Response: We sincerely thank the reviewer for noting the inappropriate use of

"ferroptotic." We have therefore removed all instances of this term from the revised manuscript. In the Discussion section (**lines 745–746**), the original phrase "thereby suppressing the ferroptotic cascade" has been changed to "thereby suppressing this iron-dependent oxidative cell death cascade."

Reviewer #3 (Remarks to the Author):

We thank the authors for carefully addressing the issues raised in our review of this manuscript. The authors have satisfactorily responded to all our concerns and comments and made changes in the manuscript accordingly. The editorial changes have also greatly improved the flow of the manuscript making the interpretation of the data easier to understand.

Response: We sincerely appreciate your positive feedback and the time and effort you have dedicated to reviewing our manuscript. Your insightful comments and suggestions have been invaluable in improving our work. Thank you again for your thoughtful and constructive contribution.

Reviewer #4 (Remarks to the Author):

I co-reviewed this manuscript with one of the reviewers who provided the listed reports. This is part of the Nature Communications initiative to facilitate training in peer review and to provide appropriate recognition for Early Career Researchers who co-review manuscripts.

Response: We appreciate your contribution to the peer review process and your willingness to co-review this manuscript. We are grateful for your time and the thoughtful comments provided. Thank you for your support and for participating in this valuable training initiative.

Point-by-point responses to Reviewers' comments (Third round of revision)

Reviewer #1 (Remarks to the Author):

I thank the authors for addressing the issues raised in my review. The authors have satisfactorily responded to all my comments and made changes accordingly.

Response: We thank the reviewer for their valuable contribution to the peer review process and for taking the time to provide thoughtful comments.
